# A High-Throughput Comparative Proteomics of Milk Fat Globule Membrane Reveals Breed and Lactation Stages Specific Variation in Protein Abundance and Functional Differences Between Milk of Saanen Dairy Goat and Holstein Bovine

**DOI:** 10.3389/fnut.2021.680683

**Published:** 2021-05-28

**Authors:** Wei Jia, Rong Zhang, Zhenbao Zhu, Lin Shi

**Affiliations:** School of Food and Biological Engineering, Shaanxi University of Science & Technology, Xi'an, China

**Keywords:** milk fat globule membrane protein, goat milk, bovine milk, Q-Orbitrap, colostrum, mature milk

## Abstract

Large variations in the bioactivities and composition of milk fat globule membrane (MFGM) proteins were observed between Saanen dairy goat and Holstein bovine at various lactation periods. In the present study, 331, 250, 182, and 248 MFGM proteins were characterized in colostrum and mature milk for the two species by Q-Orbitrap HRMS-based proteomics techniques. KEGG pathway analyses displayed that differentially expressed proteins in colostrum involved in galactose metabolism and an adipogenesis pathway, and the differentially expressed proteins in mature milk associated with lipid metabolism and a PPAR signaling pathway. These results indicated that the types and functions of MFGM proteins in goat and bovine milk were different, and goat milk had a better function of fatty acid metabolism and glucose homeostasis, which can enhance our understanding of MFGM proteins in these two species across different lactation periods, and they provide significant information for the study of lipid metabolism and glycometabolism of goat milk.

## Introduction

Milk fat is an important fraction of milk synthesized by the endoplasmic reticulum of mammary epithelial cells. It is a droplet composed of a neutral triglyceride core wrapped by a thin trilayered membrane. Milk fat globules containing cytoplasmic components are retained between the membrane layers as the fat droplets are released into milk. Therefore, milk fat is mainly composed of cholesterol, polar lipids, neutral lipids, and a protein group from the membrane and cytoplasmic crescents (1, 2). Protein, which accounts for 22–70% of the MFGM matter, not only provides protection to core milk fat but also has a series of biological functions, such as preventing infection of enteric pathogens, promoting immune and neurological functions, as well as the development of newborns (3, 4).

Due to the health benefits and nutritional values, MFGM proteins have attracted growing attention in dairy products. The major proteins in MFGM include lactadherin, mucin one, xanthine dehydrogenase/xanthine oxidase, fatty acid synthase, fatty acid-binding proteins lipophilin, and butyrophilin with physiological functions (5). For example, xanthine dehydrogenase/xanthine oxidase, one of the main MFGM enzyme proteins, has been reported to reveal antimicrobial properties and immuno-protective function. Xanthine dehydrogenase/xanthine oxidase in breast milk reacts with infant saliva to produce an effective combination of irritant and inhibitory metabolites that regulate the gut-microbiota (6). Lactadherin (milk fat globule-EGF factor 8) is a peripheral glycoprotein from human milk, which promotes the clearance and phagocytosis of apoptotic cells, and regulates the immune response (7).

With the accelerated development of proteomics technology in recent years, a large number of proteins have been identified and quantified in the milk of bovine (8), buffalo (9), donkey (10), and other mammals (11). Among them, because bovine milk is the major substitute for human milk, the comparative proteomics between bovine and human milk as well as bovine and human milk were extensively studied. Compared with bovine milk, goat milk-based dairy products may be less allergenic and more easily digested to the infants (12). In view of the unique economic significance, more and more studies have been absorbed in the nutritional and protective properties of proteins in goat milk. Chen et al. have studied the heat-dependent changes of goat milk protein, and found out that heat processing can improve protein digestibility, which was conducive to anti-atherosclerosis therapy (13). They also investigated the protein changes of goat milk during homogenization. The results showed that the homogenized goat milk proteome has changed significantly, which was mainly related to glycolysis/gluconeogenesis metabolism (14). These studies extend the understanding of protein composition in different processes. Major MFGM proteins of goat milk have been reported, which are significantly different from that of bovine. Sun et al. have characterized and compared the MFGM proteins of both Guanzhong goat and Holstein cow milk, using proteomic techniques (15). Furthermore, they analyzed and compared the MFGM proteomes of colostrum and mature milk of Xinong Saanen goat milk (16). However, the MFGM proteome is also affected by species.

Despite the poorly worldwide production of goat milk compared with the bovine, in the past years, there has been more and more interest in the in-depth characterization of its protein composition. This analysis was focused on MFGM proteins from two mammals (bovine and goat) and different lactation periods (colostrum and mature milk) to characterize the composition in conjunction with biological activity, localization, and molecular function of MFGM proteins differences related to lactation. The purpose was to reveal the differences of nutritional value and physiological states of these two species across different lactation periods to provide potential directions for infant formula and functional food development, as well as expand our current knowledge of MFGM proteome.

## Materials and Methods

### Sample Collection

The sample collection and preparation were shown in Supplementary Figure 1. The samples were collected, followed by the method reported by Sun et al. (16). The samples were collected at the Holstein bovine and Saanen dairy goat farm in Xi'an, Shaanxi province, China. Ten bovine colostrum (0–5 days postpartum), 10 mature-milk (1–6 months postpartum), 10 goat colostrum (0–5 days postpartum), and 10 mature-milk (1–6 months postpartum) samples were obtained from 20 healthy bovines and 20 healthy goats in the first lactation. All of the 40 animals were aged between 1 and 4 years old, and the animals of each species were under identical environmental conditions. Each sample of bovine and goat milk was collected twice a day and then mixed to dispel the effect of the sampling time of milk samples. These samples were transported to the laboratory on ice and stored at −80°C. Ten milk samples of each group were mixed to refrain from the influence of individual differences on MFGM protein in various lactation stages before the analysis. All handling practices involving animals carefully followed all the recommendations of the Directive 2010/63/EU of the European Parliament for the protection of animals for scientific purposes.

The extraction of MFGM proteins was conducted as described by Lu et al. (17) with minor modifications. Briefly, 50-ml milk samples were centrifuged at 12,000 × g for 40 min at 4°C. The supernatant (top layer) was transferred to another centrifuge tube and washed three times at 25°C for 10 min, with 0.1 mol L^−1^ PBS (pH 6.8) and centrifuged at 10,000 × g for 15 min at 4°C subsequently to remove residual whey proteins and caseins. Then we washed the cream twice, using ultrapure water to dislodge the residual salt ions. Finally, 0.4% SDS (1:1, *v/v*) was added to dilute the washed cream, sonicated for 1 min and centrifuged at 10,000 × g at 4°C for 40 min to separate the fat fraction. The MFGM proteins were collected in the aqueous phase (bottom layer), and their concentration was measured by BCA assay (Thermo Scientific Pierce BCA protein assay kit, USA).

### Protein Digestion

The MFGM protein was reduced, alkylated, and digested, and followed the method reported by Lu et al. (18). For each milk sample, three independent biological replicates were made. First, 10-μL MFGM protein was dissolved in 100-μL, 50-mmol L^−1^ NH_4_HCO_3_, and then 10-μL, 100-mmol L^−1^ dithiothreitol was added and incubated at 56°C for 30 min. Subsequently, the MFGM protein was alkylated with 15 μL of 55-mmol L^−1^ iodoacetamide in dark for 30 min at room temperature and then adding sequencing grade-modified trypsin to digest the MFGM protein at a ratio of 1:100 enzyme/protein for 16–18 h at 37°C and terminated the reaction by adding 1% formic acid. Finally, the peptides mixture was desalting by Oasis HLB cartridges (Waters Cooperation, Milford, MA, USA), dried by a vacuum centrifuge and then resuspended in 40 μL of 0.1% (*v/v*) formic acid.

### Liquid Chromatography Tandem Q-Orbitrap Mass Spectrometry

Peptide separation was performed by EASY-nLC 1000 system (Thermo Scientific, San Jose, CA), equipped with a C18-reverse phase column (75-μm inner diameter, 10-cm long, 3-μm resin; Thermo Scientific) at 200 nL/min and 35°C for a total run time of 100 min. Solution A (0.1% formic acid in water) and solution B (0.1% formic acid in 80% ACN) were used as eluents for the peptide separation according to the following elution gradients: 5–35% solution B for 50 min; 35–100% solution B for 25 min, followed by 15-min washing with 100% solution B, and return to 5% B in 0.1 min, re-equilibration during 9.9 min with 5% solution B.

The peptide eluted from the column was ionized by a Q-Exactive (Thermo Fisher Scientific, Waltham, USA) mass spectrometer in a positive mode. The spray voltage was operated at 3.8 kV. The *m*/*z* scan range of single MS scans of peptide precursors was *m*/*z* 300–1,700 at a resolution of 70,000 (at *m*/*z* 200). The AGC (automatic gain control) was 3 e^6^, and the maximum injection time was 200 ms. The top 20 most intense precursor ions with charge ≥2 determined by MS scan were used to obtain MS/MS data at a resolution of 17,500 by using a higher normalized collision energy of 27 eV. The AGC was 1 e^5^ and the maximum injection time was 50 ms. In order to avoid superfluous fragmentation, the dynamic exclusion time was set to 30 s.

### Data Analysis

The raw LC-MS/MS files with three replicates were obtained for MFGM proteins of each milk group. Two proteins identified from three biological replicates of each milk group were used for subsequent analysis. The data analysis was carried out using the MaxQuant software (Max Planck Institute of Biochemistry, Martinsried, Germany, version 1.6.7.0), with Andromeda as a peptide search engine (Matrix Science, version 2.4), and searched against the database of *Caprinae* (67,040 entries, 02/08/2019) and *Bos taurus* (64,796 entries, 02/08/2019) organism group with reverse sequences generated by MaxQuant.

Search parameters were a first search peptide mass tolerance of 20 ppm and main search peptide mass tolerance of 4.5 ppm. Methionine oxidation and protein N-terminal acetylation were defined as variable modification and carbamidomethyl of cysteine defined as a fixed modification for both identification and quantification. A trypsin/P was adjusted as a proteolytic enzyme with a maximum of two missed cleavages. A maximum of 0.01 false discovery rates (FDRs) and at least two unique peptides for each protein were demanded for reliable identification and quantification. Label-free quantification (LFQ) was enabled in MaxQuant.

The MFGM proteins identified in at least two of the three biological replicates were used for subsequent statistical analysis. The LFQ intensity of identified proteins was analyzed using one-way ANOVA test. MFGM proteins with *p* < 0.05 and fold change >2 in the relative abundance ratios were considered to be differentially expressed MFGM proteins (DEMPs) between milk groups. Principal component analysis (PCA) and partial least squares regression-discriminant analysis (PLS-DA) were used to construct the recognition model and prediction models, respectively, among milk groups by using SIMCA 15 (19).

### Bioinformatics Analyses

The molecular function, cellular component, and biological process of all identified MFGM proteins according to their gene ontology (GO) annotations and Kyoto Encyclopedia of Genes and Genomes (KEGG) pathway analysis were achieved, using DAVID Bioinformatics Resources 6.8 (https://david.ncifcrf.gov/). Conversion of the genes of MFGM proteins was using Retrieve/ID mapping (https://www.uniprot.org/uploadlists/). Protein–protein interaction (PPI) network construction was performed, using STRING (https://string-db.org/), with the DEMPs displayed by proteomic data used as input. Statistical differences were proclaimed significantly if *p* ≤ 0.05.

## Results and Discussion

### Component Analysis of MFGM Proteins From Different Milk Groups

**Figure 1:**
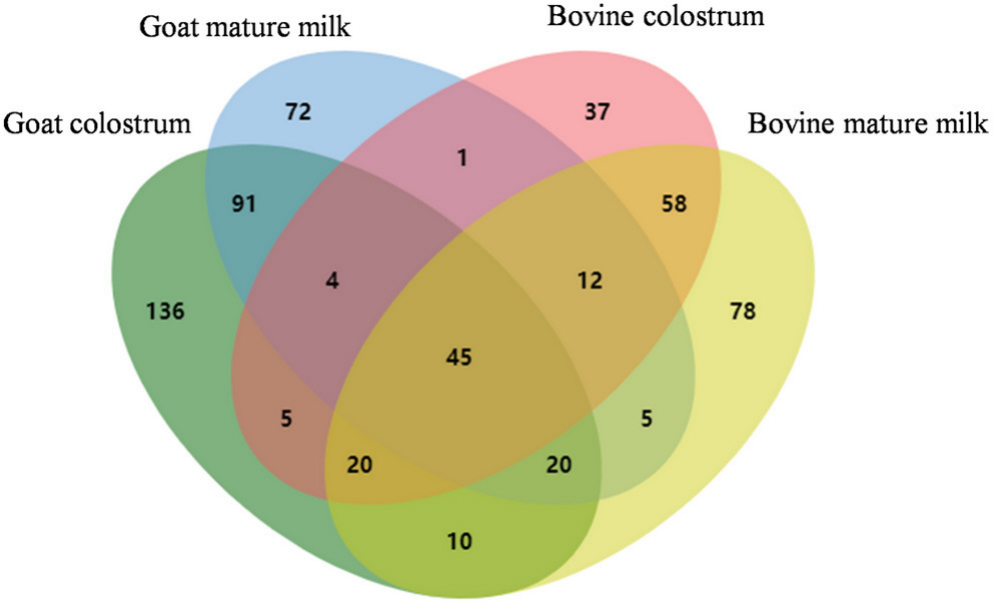
Venn diagram analysis of the identified MFGM protein components from bovine colostrum, bovine mature milk, goat colostrum, and goat mature milk.

In this study, 331 MFGM proteins in goat colostrum (GC), 250 in goat mature milk (GM), 182 in bovine colostrum (BC), and 248 in bovine mature milk (BM) were identified and quantified using LC-Q-Orbitrap mass spectrometer (Supplementary Table 1). These proteins spanned more than six orders of magnitude. As shown in Figure 1, there were 136, 72, 37, and 78 uniquely expressed MFGM proteins identified in goat colostrum, goat mature milk, bovine colostrum, and bovine mature milk, respectively. The uniquely expressed proteins in goat colostrum included calreticulin, cofilin-1, and methanethiol oxidase in goat mature milk included FA complementation group I, phosphatidylethanol-amine binding protein 4, and perilipin-1. Approximately, 45 MFGM proteins, coupled with cream fractions, were identified in four milk groups, including the complement component 3, fatty acid synthase, sodium-dependent phosphate transport protein 2B, and pericentrin, indicating that goat milk was a substitute for bovine milk to some extent in the research and development of infant milk powder and functional food, and also providing an orientation for the further functional development of goat milk.

In addition, proteins from caseins and whey, such as αs1-casein, αs2-casein, β-casein, κ-casein, lactoferrin, and β-lactoglobulin, were also identified in the MFGM fractions, which could be owing to the residual contamination of the proteins during MFGM extraction (20). In our study, polymeric immunoglobulin receptor (PIGR) was highly abundant in four milk groups. PIGR binds with polymerized immunoglobulin A and immunoglobulin M and transport them to perform immune functions across cell membranes (21). The other identified immunoglobulin proteins included IGL@ protein (IGL@), IGK protein (IGK), immunoglobulin heavy-constant mu (IGHM), and immunoglobulin J chain (JCHAIN). Previously observed major proteins, such as lactadherin, butyrophilin, and xanthine dehydrogenase/oxidase, were conserved in MFGM across four groups, which indicate the robustness of the methodology.

### Chemometrics of MFGM Protein in Various Lactation Periods

Before chemometric analysis of MFGM protein, preprocessing of LC-MS/MS data was implemented. The correct normalization of each milk group can eliminate the systematically differences between features. However, the total peptide ion signals necessary to perform LC-MS/MS were distributed over several adjacent runs. Therefore, it was necessary to know the normalization coefficient (N) of each fraction to sum the peptide ion signal. Based on the least overall proteome variation, the quantities of proteins can be determined via a global optimization procedure after the intensities were normalized to a normalization factor as free variables. Hence, in sample A, the total intensity of a peptide ion P was defined as

$$\begin{array}{l} Intensity_{P,A}\left(N\right)=\underset{k}{\sum }N_{run\left(k\right)}XIC_{K} \end{array}$$

where the index *k* covered all isotope patterns of peptide ion P in sample A.

A triangular matrix containing all paired protein ratios between any two samples was constructed. This matrix corresponds to the overdetermined system of equations for the protein abundance distributions in the sample. A subsequent least-squares analysis was performed to reconstruct the abundance profile based on the sum of squared differences in the matrix via the optimal satisfaction of individual protein ratios.

$$\begin{array}{l} \underset{\left(j,k\right)\in valid\;pairs}{\sum }{\left(\log r_{j,k}-\log I_{j}+\log I_{k}\right)}^{2} \end{array}$$

Then the whole profile was rescaled to the cumulative intensity of the samples, thereby retaining the total summed intensity of the protein over all samples, which was the “LFQ intensity” (22).

**Figure 2:**
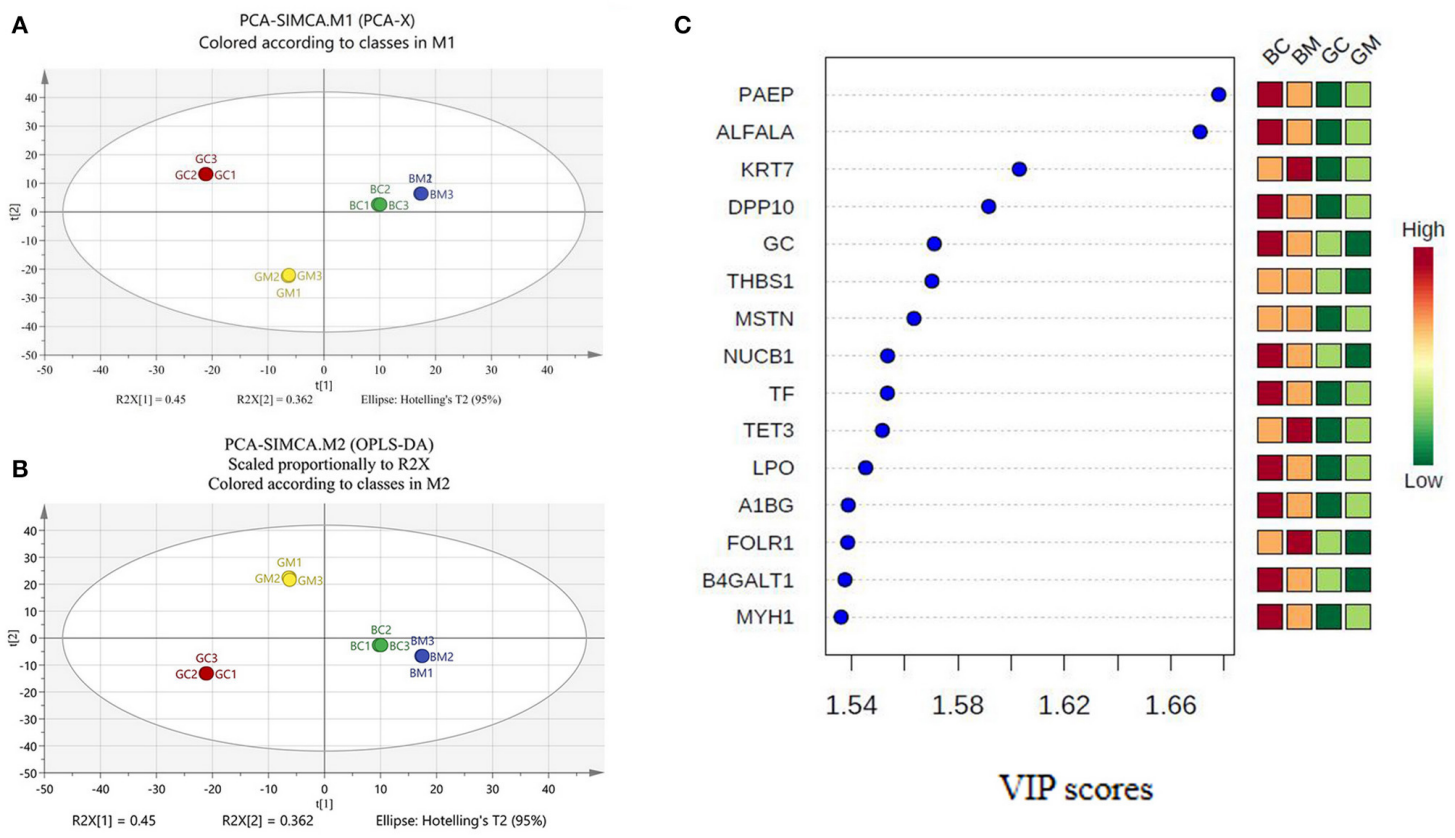
PCA and PLS-DA analysis of MFGM proteins in bovine and goat milk. **(A)** Principal components analysis (PCA) scores based on intensities of proteins. **(B)** Partial least squares discrimination analysis (PLS-DA) scores based on intensities of proteins. **(C)** VIP scores for differential protein.

Chemometric analysis is a technique involving statistical methods to comprehend chemical information generated by analytical instruments. To evaluate whether the data of proteomic analysis can be engaged to visually differentiate the four group milk samples, PCA analysis as an unsupervised data analysis was carried out by loading the LFQ intensities of all detected proteins as variables and the different MFGM matrices as observation points. In this case, the data from all the replicates were used. A clear separation of all MFGM matrices can be observed on the PCA score plot (Figure 2A) based on the first two principal components. Samples close to each other on the PCA score plot revealed similar properties, while the milk samples far from each other revealed dissimilar molecular weight in protein mass spectrographic analysis. The first two principal components accounted for 81.2% [*R*^2^*X* (1) + *R*^2^*X* (2)] of the total variation in the data. No outlier was observed by ellipse Hotelling's T2. The first principal component (45% of the total variation) clearly divided the samples into four well-separated clusters, corresponding to the four groups, showed that the distribution differences among the goat colostrum, goat mature milk, bovine colostrum, and bovine mature milk were mainly due to biological reasons. Meanwhile, *R*^2^*X* (cum) was cumulative *R*^2^*X* up to the specified component, where *R*^2^*X* was the fraction of X variation modeled in the component. *Q*^2^ (cum) was the cumulative *Q*^2^ up to the specified component, where *Q*^2^ was overall cross-validated *R*^2^*X*. *R*^2^ (cum) and *Q*^2^ (cum) were the critical parameters to evaluate the quality of the PCA model, which, respectively, reflect the degree of interpretation of the principal components to *X* variables and the predictive ability of the model. Figure 2A showed that *R*^2^*X* (cum) was 0.812 and *Q*^2^ (cum) was 0.966, illustrating that the PCA model was stable and predictable.

**Figure 3:**
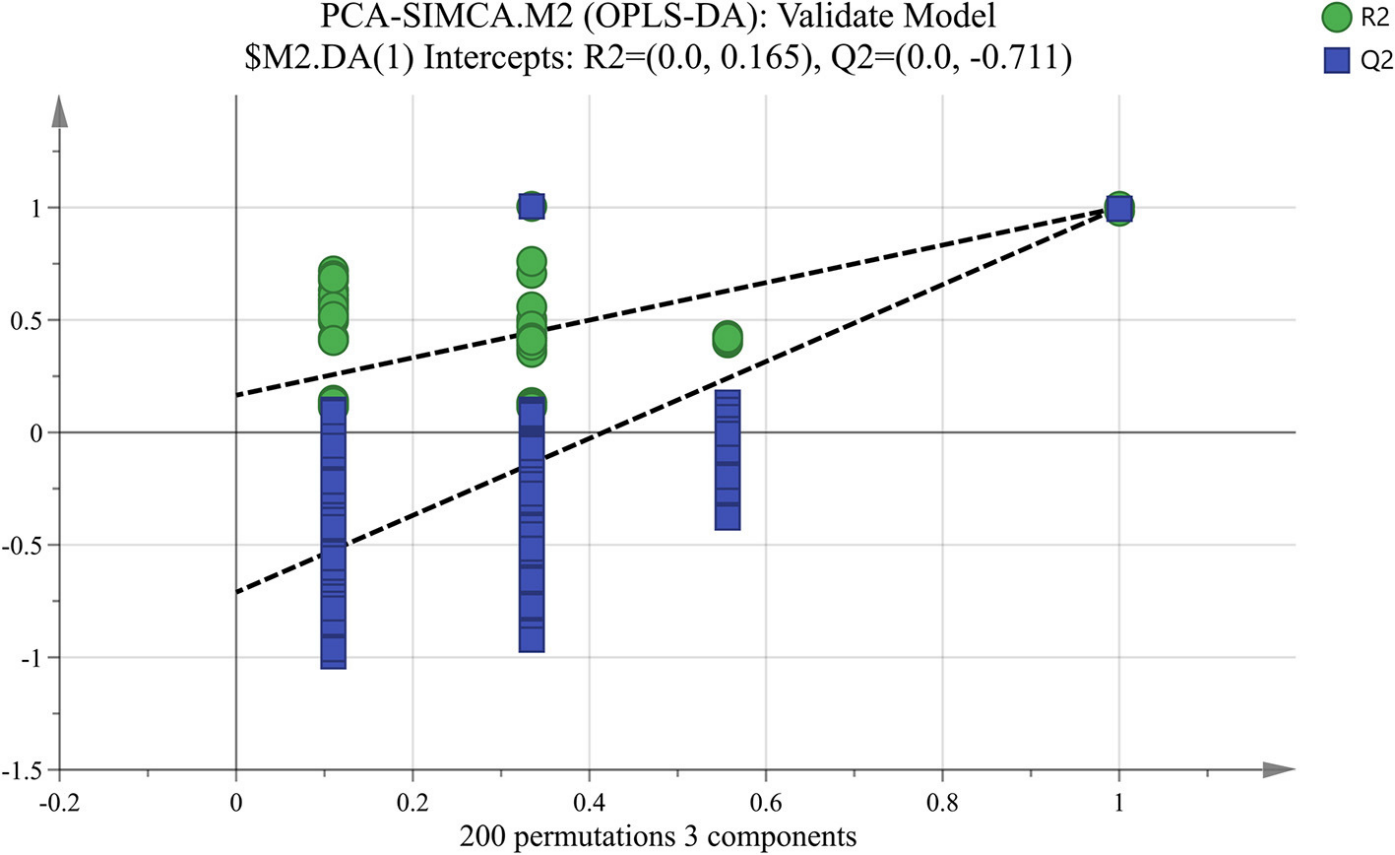
Permutation test of PLS-DA model.

PLS-DA is a chemical projection method that associates X and Y variable blocks via a linear multivariable model. The objective is to find the direction in X space that divides the classes according to the sample set with known class members (23, 24). In the built PLS-DA model, *R*^2^X (cum) was 0.812, *R*^2^Y (cum) was 1, and *Q*^2^ (cum) was 1, which denoted that the PLS-DA model has good fitting ability and prediction performance (Figure 2B). VIP scores were shown in Figure 2C, and variables with VIP scores higher than 1 were considered to have significantly contributed to the model. To evaluate the stability and reliability of PLS-DA model, cross-validation was adopted. The total correct classification rate of four MFGM proteins was 100%. The statistical significance of the predictive quality parameters in the built PLS-DA model was validated by 200 permutation tests (Figure 3). The Y-intercept of *R*^2^ and *Q*^2^ was 0.165 and −0.711, respectively, which ensured that the PLS-DA model was not overfitting.

### DEMPs, Respectively, in Colostrum and Mature Milk of Goat and Bovine

Three hundred and thirty-one MFGM proteins were identified in goat colostrum and 182 in bovine colostrum. *T*-test and fold change analysis were used to analyze the difference of the MFGM proteins in colostrum of goat and bovine, and the MFGM protein with *p* < 0.05 and at least two-fold was considered as the cutoff criteria of differential expression. As shown in Table 1, among 74 common proteins in colostrum of goat and bovine, 49 were differentially expressed. We found out that there were 22 upregulated and 27 downregulated MFGM proteins in goat colostrum. The levels of sodium/nucleoside cotransporter, lipoprotein lipase, sodium-dependent phosphate transport protein 2B, xanthine dehydrogenase/oxidase, and fatty acid synthase were higher in goat colostrum, while the levels of cathelicidin-1, lipopolysaccharide-binding protein, alpha-enolase, and vitamin D-binding protein were higher in bovine colostrum.

**Table 1:** DEMPs in colostrum of goat and bovine (*p* < 0.05, fold change >2).

**Protein names**	**Gene names**	**Length**	**Fold change**	***P*-value**	**FDR**	**Change**
Beta-lactoglobulin	LGB	180	646.62	5.61E-08	9.96E-07	↑
Ras-related protein Rab-18	RAB18	206	130.97	1.38E-05	4.66E-05	↑
Fatty acid synthase	FASN	2510	125.88	1.29E-07	1.83E-06	↑
Sodium-dependent phosphate transport protein 2B	SLC34A2	693	121.27	3.27E-08	7.74E-07	↑
Perilipin-3	PLIN3	427	73.318	1.78E-07	2.11E-06	↑
ATP-binding cassette sub-family G member 2	ABCG2	643	72.187	6.15E-07	4.37E-06	↑
Polyamine modulated factor 1 binding protein 1	PMFBP1	869	55.507	2.39E-07	2.43E-06	↑
Sodium/nucleoside cotransporter	SLC28A3	697	36.345	3.94E-06	1.65E-05	↑
Platelet glycoprotein 4	CD36	472	23.364	1.50E-06	8.21E-06	↑
Xanthine dehydrogenase/oxidase	XDH	1332	18.968	1.23E-05	4.38E-05	↑
Perilipin-2	PLIN2	378	18.244	6.52E-06	2.57E-05	↑
Lipoprotein lipase	LPL	460	7.0478	4.52E-05	0.00011896	↑
Actin, cytoplasmic 1	ACTB	375	5.9809	4.93E-05	0.00012497	↑
Elongation factor 1-alpha 1	EEF1A1	462	5.7187	0.00010212	0.00023388	↑
Beta-casein	CSN2	222	4.8173	0.0075796	0.010552	↑
Keratin, type I cytoskeletal 9	KRT9	623	3.992	0.00025127	0.00049556	↑
Ezrin	EZR	581	3.2147	7.35E-05	0.00017395	↑
Annexin A2	ANXA2	339	2.8804	0.00044768	0.000815	↑
Histone H4	HIS4	103	2.5118	0.0033904	0.0051217	↑
Keratin, type II cytoskeletal 79	KRT79	535	2.4297	0.0011796	0.0019477	↑
L-lactate dehydrogenase B chain	LDHB	334	2.3079	0.0013485	0.002176	↑
Fatty acid-binding protein, heart	FABP3	133	2.0861	0.001011	0.0017091	↑
Lipocalin 2	LCN2	200	0.49736	0.0026464	0.0040847	↓
Biglycan	BGN	369	0.38809	0.00010601	0.00023521	↓
Alpha-S2-casein	CSN1S2	223	0.377	0.00031978	0.00061363	↓
Nucleoside diphosphate kinase B	NME2	152	0.36189	0.00011133	0.00023952	↓
Fibronectin	FN1	2137	0.34769	6.17E-05	0.00015111	↓
Beta-1,4-galactosyltransferase 1	B4GALT1	402	0.28188	0.00044646	0.000815	↓
Alpha-lactalbumin	LALBA	142	0.28062	0.0072975	0.010483	↓
Alpha-S1-casein	CSN1S1	214	0.23612	2.50E-05	7.40E-05	↓
Butyrophilin subfamily 1 member A1	BTN1A1	526	0.18323	0.00022928	0.00046511	↓
Vitamin D-binding protein	GC	468	0.17975	0.00058113	0.0010315	↓
Alpha-1-antiproteinase	SERPINA1	416	0.14662	7.24E-06	2.70E-05	↓
Complement component 3	C3	1661	0.13906	3.93E-05	0.00010742	↓
Alpha-enolase	ENO1	416	0.12511	1.25E-06	8.05E-06	↓
Folate receptor alpha	FOLR1	251	0.083225	0.00096249	0.0016667	↓
Hemopexin	HPX	459	0.066022	2.21E-05	6.81E-05	↓
Lipopolysaccharide-binding protein	LBP	481	0.063899	0.00012828	0.00026787	↓
Alpha-2-HS-glycoprotein	AHSG	359	0.048166	3.47E-06	1.54E-05	↓
Nucleobindin-1	NUCB1	460	0.047585	1.55E-05	5.01E-05	↓
Polymeric immunoglobulin receptor	PIGR	758	0.036382	3.75E-05	0.00010642	↓
Thrombospondin-1	THBS1	1170	0.026118	1.45E-06	8.21E-06	↓
Keratin type II cytoskeletal 71	KRT71	368	0.021154	1.86E-06	9.45E-06	↓
HHIP like 2	HHIPL2	727	0.0096536	3.50E-07	3.10E-06	↓
Actin, alpha skeletal muscle	ACTA1	377	0.0051483	0.0020504	0.003235	↓
Keratin, type II cytoskeletal 7	KRT7	452	0.0025367	3.20E-08	7.74E-07	↓
Serotransferrin-like	LOC525947	622	0.0011206	2.85E-06	1.35E-05	↓
Myostatin	MSTN	124	0.00076419	4.74E-07	3.74E-06	↓
Dipeptidyl-peptidase 10	DPP10	789	4.22E-05	5.84E-09	4.14E-07	↓

*↑ means a higher protein abundance in goat colostrum than in bovine colostrum*.

Two hundred and fifty MFGM proteins were identified in goat mature milk and 248 in bovine mature milk. As shown in Table 2, among 90 common proteins in mature milk of goat and bovine, 63 were differentially expressed on the basis of *p* < 0.05 and at least two-fold, with 32 upregulated and 31 downregulated MFGM proteins. The levels of lipoprotein lipase, fatty acid synthase, sodium-dependent phosphate transport protein 2B, and apolipoprotein E were higher in goat mature milk, while the levels of lipocalin 2, sodium/nucleoside cotransporter, apolipoprotein A-I, and polymeric immunoglobulin receptor were lower in bovine mature milk than in goat. The abundance of lipoprotein lipase and fatty acid synthase was higher in goat mature milk, which was the same as that in colostrum. Lipopolysaccharide-binding protein was higher in bovine mature milk in contrary. Lipocalin-2 can bind and eliminate enterochelin, a high-affinity siderophore, to reduce the accessibility of bacteria to iron and inhibit its growth, and participate in the modeling of immune response. Apolipoprotein A-I is a kind of lipoprotein expressed by glial cells, which is strongly induced in aging, injury or neurodegeneration, involved in the peripheral metabolic regulation and lipid processing of chylomicron, the occurrence and development of Parkinson's disease and Alzheimer's disease, and may play a neuroprotective role in the brain (16).

**Table 2:** DEMPs in mature milk of goat and bovine (*p* < 0.05, fold change >2).

**Protein names**	**Gene names**	**Length**	**Fold change**	***p*-value**	**FDR**	**Change**
Lipoprotein lipase	LPL	460	423.15	2.46E-05	9.08E-05	↑
Peptidylprolyl isomerase	FKBP11	191	136.83	0.0048971	0.0069376	↑
Fructose-bisphosphate aldolase	ALDOC	364	48.478	0.0085618	0.011371	↑
G_PROTEIN_RECEP_F1_2 domain-containing protein	OR10G2	318	47.151	6.10E-06	3.49E-05	↑
ATP-binding cassette sub-family G member 2	ABCG2	643	44.222	5.24E-07	2.48E-05	↑
Olfactory receptor	OR6S1	331	42.46	2.61E-05	9.26E-05	↑
Fatty acid synthase	FASN	2510	42.394	3.32E-06	2.57E-05	↑
Myosin-7	MYH7	1937	30.577	6.98E-06	3.49E-05	↑
Keratin, type II cytoskeletal 2 epidermal	KRT2	639	21.21	6.32E-06	3.49E-05	↑
Ubiquitin-60S ribosomal protein L40	UBA52	128	18.063	0.025215	0.029768	↑
Alpha-S2-casein	CSN1S2	223	14.265	0.00038683	0.00080338	↑
Histone H2A	H2AFV	126	14.17	8.60E-06	4.06E-05	↑
Perilipin-3	PLIN3	427	11.71	0.01536	0.018922	↑
Sodium-dependent phosphate transport protein 2B	SLC34A2	693	11.603	3.13E-05	0.00010219	↑
AMP-binding domain-containing protein	ACSL1	660	11.577	3.01E-05	0.00010219	↑
Melanotransferrin	MELTF	739	11.557	3.94E-06	2.79E-05	↑
Cathelicidin-1	CATHL1A	155	9.9987	9.04E-05	0.00025603	↑
PHB domain-containing protein	STOM	279	5.0903	0.00029065	0.00063347	↑
Actin, aortic smooth muscle	ACTA2	375	4.9144	0.00068203	0.0012883	↑
Butyrophilin subfamily 1 member A1	BTN1A1	526	4.906	9.92E-05	0.00027192	↑
C-type lectin domain-containing protein	LOC101123029	315	4.8752	3.91E-05	0.00012322	↑
Actin, cytoplasmic 1	ACTB	375	4.8116	0.00010826	0.00027884	↑
Glycosylation-dependent cell adhesion molecule 1	GLYCAM1	152	3.9552	0.0043649	0.0062885	↑
Apolipoprotein E	APOE	316	3.5259	0.0010421	0.0018212	↑
Beta-casein	CSN2	222	3.284	0.03291	0.037802	↑
14-3-3 protein theta	YWHAQ	245	3.2822	0.00017024	0.00040486	↑
Fmp27_GFWDK domain-containing protein	KIAA0100	2213	3.0707	0.00046116	0.00093329	↑
Ras-related protein Rab-2A	RAB2A	212	3.0065	0.00096506	0.0017453	↑
Annexin A5	ANXA5	319	2.6088	0.0013013	0.0021271	↑
Ras-related protein Rab-11A	RAB11A	208	2.4342	0.00074376	0.0013743	↑
Keratin, type I cytoskeletal 14	KRT14	472	2.1997	0.0063351	0.0088276	↑
Keratin, type II cytoskeletal 5	KRT5	590	2.0897	0.021206	0.02575	↑
Terpene cyclase/mutase family member	LSS	733	0.4125	0.00052237	0.0010326	↓
Ras-related protein Rab-1B	RAB1B	147	0.40606	0.00064711	0.0012501	↓
Actin, cytoplasmic 2	ACTG1	344	0.38809	0.0025025	0.0037318	↓
Keratin, type II cytoskeletal 1	KRT1	644	0.3616	0.0002435	0.00054466	↓
Keratin, type II cytoskeletal 6C	KRT6C	564	0.28456	0.001155	0.0019635	↓
Lipocalin 2	LCN2	200	0.27997	0.00010254	0.00027238	↓
Fibrinogen C-terminal domain-containing protein	FGG	427	0.18828	0.00023213	0.00053328	↓
Peptidoglycan-recognition protein	PGLYRP1	190	0.18728	1.34E-05	5.68E-05	↓
Beta-lactoglobulin	LGB	180	0.18084	4.79E-05	0.0001454	↓
Keratin, type I cytoskeletal 18	Krt18	423	0.16661	0.00038751	0.00080338	↓
Creatine kinase M-type	CKM	381	0.16391	0.037579	0.04259	↓
Kappa casein	CSN3	141	0.14848	2.05E-05	7.91E-05	↓
Lipopolysaccharide-binding protein	LBP	481	0.14279	1.89E-05	7.66E-05	↓
Keratin, type II cytoskeletal 79	KRT79	535	0.13909	2.07E-06	2.57E-05	↓
Sodium/nucleoside cotransporter	SLC28A3	697	0.13764	0.02702	0.031461	↓
Apolipoprotein A-I	APOA1	259	0.12726	0.00012728	0.00031821	↓
Myoglobin	MB	154	0.11156	6.18E-06	3.49E-05	↓
Lactoferrin	LTF	690	0.10557	6.30E-05	0.00018474	↓
Keratin, type I cytoskeletal 10	KRT10	584	0.094047	3.11E-06	2.57E-05	↓
Serum albumin	ALB	607	0.072077	1.20E-05	5.38E-05	↓
Lactadherin	MFGE8	398	0.043848	2.80E-06	2.57E-05	↓
Antithrombin-III	SERPINC1	530	0.020072	6.58E-06	3.49E-05	↓
Actin, alpha skeletal muscle	ACTA1	377	0.016074	0.0027322	0.0040041	↓
Keratin type II cytoskeletal 71	KRT71	368	0.01374	0.00017147	0.00040486	↓
Alpha-S1-casein	CSN1S1	214	0.011364	3.22E-06	2.57E-05	↓
Serpin A3-1	SERPINA3-1	411	0.011092	2.67E-06	2.57E-05	↓
Tektin-4	TEKT4	446	0.0096822	1.58E-06	2.57E-05	↓
Clusterin	CLU	439	0.0082354	1.32E-06	2.57E-05	↓
Pericentrin	PCNT	3085	0.0049584	1.46E-06	2.57E-05	↓
Serum amyloid A-3 protein	SAA3	112	0.0046671	5.83E-07	2.48E-05	↓
Polymeric immunoglobulin receptor	PIGR	758	0.0020233	0.0010498	0.0018212	↓

*↑ means a higher protein abundance in goat mature milk than in bovine mature milk*.

### GO, KEGG Pathway, and PPI Analysis of the DEMPs

**Figure 4:**
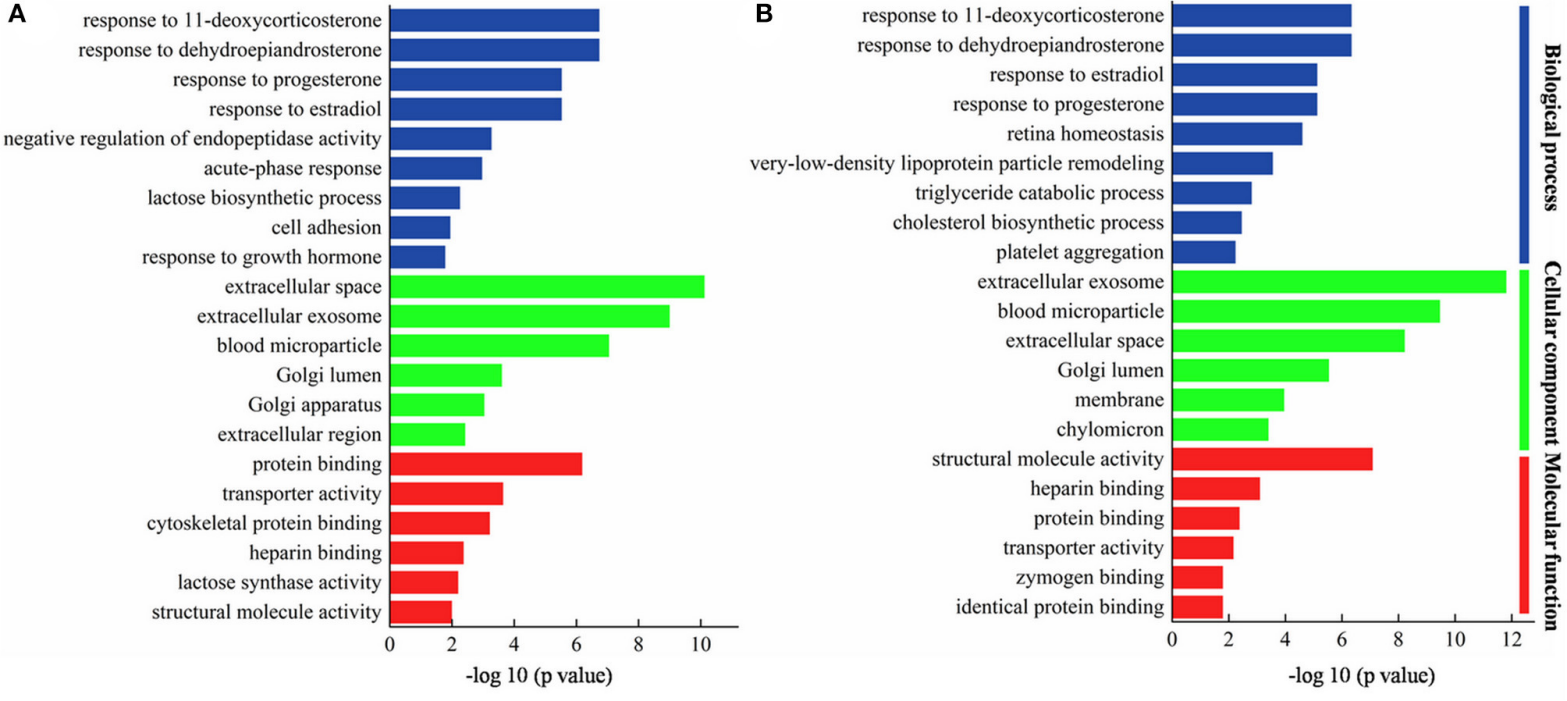
Gene ontology (GO) of DEMPs in colostrum of goat and bovine **(A)**, in mature milk of goat and bovine **(B)**.

For the purpose of comparing the biological functions of MFGM proteins in colostrum, 49 DEMPs in colostrum of goat and bovine were analyzed by gene ontology (GO) functional annotation, which was divided into three categories of molecular function (MF), cellular components (CC), and biological process (BP). This was useful to further understand the colostrum MFGM proteins functions in goat and bovine. The most significant enrichment annotation information (*p* < 0.05) in each branch was shown in Figure 4A, in which the prevalent biological processes were a response to dehydroepiandrosterone, response to 11-deoxycorticosterone, response to estradiol, and response to progesterone. Others were involved in negative regulation of endopeptidase activity, acute-phase response, lactose biosynthetic process, and cell adhesion. The MFGM proteins were highly enriched in extracellular space, extracellular exosome, and blood microparticle, which illustrated that the majority of these proteins present due to leakage of the protein from the blood serum into the milk at the tight junctions in the cells in the mammary gland. Other enriched origin categories were Golgi lumen, Golgi apparatus, and extracellular region. Three prominent molecular functions of DEMPs in colostrum of goat and bovine were protein binding, transporter activity, and cytoskeletal protein binding, which was consistent with the result of Cunsolo et al. (25). Lactose synthase activity and structural molecule activity were also significantly represented.

The most significant enrichment annotation information (*p* < 0.05) in each branch of mature milk was shown in Figure 4B, in which the prevalent biological processes were response to dehydroepiandrosterone, response to 11-deoxycorticosterone, response to progesterone, and response to estradiol. It was consistent with the biological processes involved in colostrum, indicating that these biological processes occurred in the whole lactation period. In stressful situations, the adrenal cortex reacted to ACTH and began to secrete dehydroepiandrosterone that had been proved to exert anti-inflammatory and antioxidant effects, and played a protective and regenerative role (26). Others were involved in very-low-density lipoprotein particle remodeling, triglyceride catabolic process, and cholesterol biosynthetic process. The MFGM proteins were highly enriched in extracellular exosome, blood microparticle, and extracellular space, which was similar to the DEMPs in colostrum of goat and bovine. Other enriched origin categories were Golgi lumen, membrane, and chylomicron. The prominent molecular functions were structural molecule activity, heparin binding, and protein binding.

**Figure 5:**
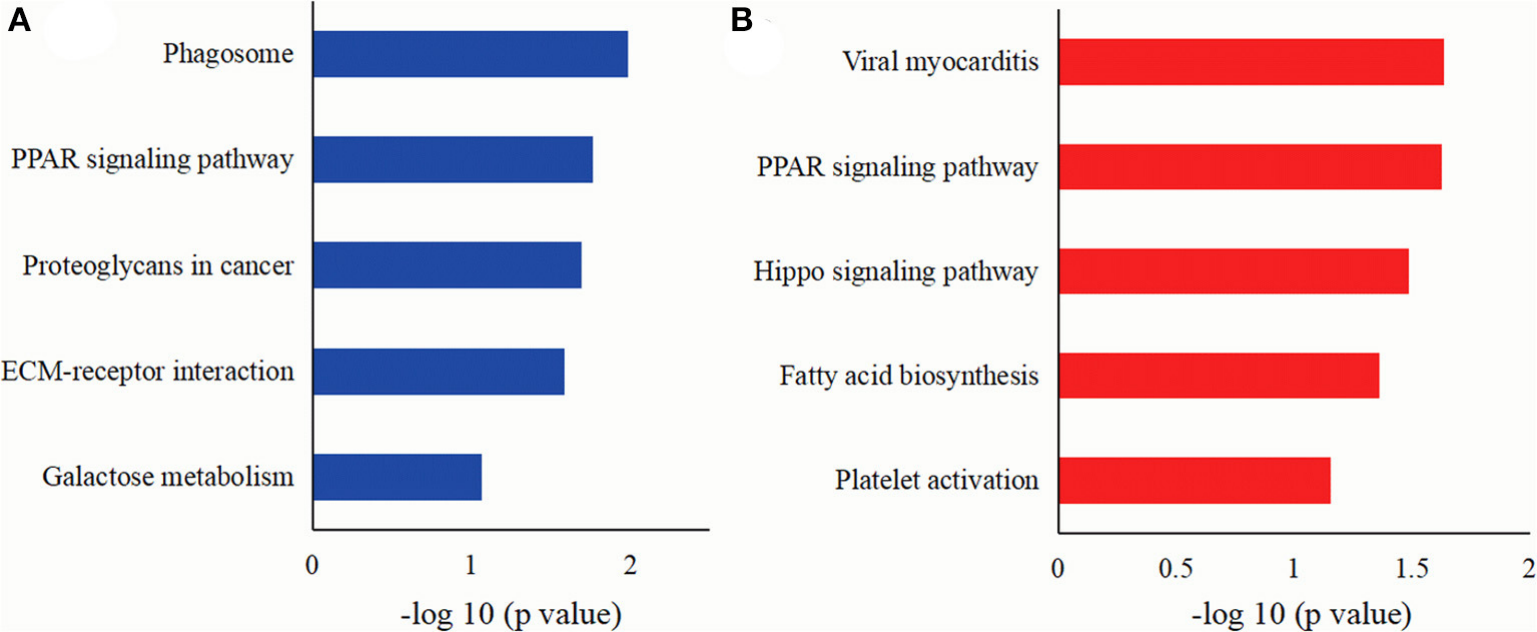
The KEGG pathways of DEMPs colostrum and mature milk of goat and bovine **(A)**, in mature milk of goat and bovine **(B)**.

A KEGG pathway was employed to analyze the main pathways of MFGM protein differentially expressed in colostrum of goat and bovine. As shown in Figure 5A, the DEMPs mainly involved in the pathways of the phagosome, a PPAR signaling pathway, proteoglycans in cancer, ECM-receptor interaction, and galactose metabolism. As shown in Figure 5B, the DEMPs in mature milk mainly involved in the pathways of viral myocarditis, a PPAR signaling pathway, salmonella infection, and fatty acid biosynthesis.

PPI analysis of DEMPs in the colostrum and mature milk of goat and bovine was implemented to obtain a color-coded network, revealing the correlation between DEMPs (Supplementary Figure 2). The final network of colostrum consists of 49 nodes (proteins) and 99 edges (interactions). The avg. local clustering coefficient was 0.533, and the PPI enrichment *p-*value was lower than 1.0 e^−16^, indicating that the 49 DEMPs were biologically connected. Most highly interacting protein nodes in colostrum were divided into five communities, including a cellular process, cell adhesion, immune response, lactose synthase activity, and a metabolic process. Similarly, the final network of mature milk consists of 63 nodes (proteins) and 148 edges (interactions). The avg. local clustering coefficient and *p-*value were 0.575 and lower than 1.0 e^−16^, respectively, indicating that the 63 DEMPs were biologically connected. Furthermore, these highly interacting protein nodes were divided into three communities, including a cellular process, cell adhesion, and response to extracellular stimulus. Consequently, in light of the bioinformatics analysis of proteomics, the MFGM proteins in colostrum might be led to the intervention of related health issues, while the bioactivities of MFGM proteins between goat and bovine might be different.

## Discussion

### Protein Profile Analysis Based on Chemometrics

A label-free proteomic approach was employed to identify the MFGM proteins in goat and bovine milk. Compared with the previous reports, 102 MFGM proteins, mainly calcium-binding protein, cytoskeletal protein, and intercellular signal molecule were added in bovine milk, 114 in goat milk, mainly a metabolite interconversion enzyme, translational protein, cytoskeletal protein, and nucleic acid-binding protein [Supplementary Table 2; (15–17, 27)]. The nucleotide-binding proteins in this study were mainly DNA helicase, followed by several members of Ras super family (Rhos and Rabs). All these proteins are likely involved in the secretion of secreted milk components by vesicle (17). Furthermore, the results of PCA, PLS-DA, and VIP analysis found out that nucleobindin-1 (NUCB1), folate receptor alpha (FOLR1), vitamin D-binding protein (GC), thrombospondin-1 (THBS1), and beta-1,4-galactosyltransferase 1 (B4GALT1), with the lower abundance in goat mature milk than in other three milk groups, can be used as markers to distinguish the four milk groups. NUCB1 is Golgi-localized soluble protein, which contains multiple putative functional domains. NUCB1 localized in extracellular was considered to be a regulator of matrix maturation in bone (28, 29). FOLR1 can internalize folates into the cells, which is crucial to DNA repair and synthesis, and mediate the activation of pro-oncogene STAT3, which contributes to angiogenesis, tumor proliferation, and metastasis (30). THBS1 has been demonstrated to participate in mechano-signal transduction and is specific at the level of apoptosis induction (31). GC has the metabolic effects of influencing bone metabolism, chemotaxis, actin scavenging, innate immunity, modulation of inflammatory processes, and binding of fatty acids (32). As mentioned above, the data from our study not only provide a comprehensive understanding of the MFGM protein compositions among the four milk samples but also reveal the differences of MFGM proteins among different species of mammals. The results exhibited a scientific basis for the development of functional products, using goat milk.

A number of MFGM proteins were obviously differentially expressed in the goat and bovine milk, whose functions may associate with the flavor and protection of the lamb or calf from infections. Milk fatty acids are extracted from the arterial blood or synthesized *de novo* in the mammary gland and involve many kinds of mammary enzymes, including lipoprotein lipase and fatty acid synthetase (33). The abundance of lipoprotein lipase was higher in colostrum of goat milk than that of bovine milk. Lipoprotein lipase was more closely bound with fat globules and has a better correlation with spontaneous lipolysis in goat milk. It can release fatty acids from lipoproteins and chyle particles, which may relate to the differences in the flavor of goat and bovine milk. Zhu et al. (34) have clarified that inhibition at the gene-expression level of fatty acid synthetase restrained the accumulation of TAG and the formation of lipid droplet by reducing esterification and lipogenesis and promoting lipolysis in goat milk. In addition, fatty acid synthetase also helped generate toll-like receptor 4 (TLR4), which is vital for lipid metabolism regulation. Lipopolysaccharide-binding protein, a 58–60 kDa protein, catalyzed the transfer of bacterial lipopolysaccharide to CD14, which exists in soluble form and facilitates lipopolysaccharide presentation to TLR4 as a cell surface receptor. This activates the intracellular signaling pathways and promotes the upregulation of adhesion molecules and proinflammatory cytokines, which are participated in the innate immune response (35). The low abundance of lipopolysaccharide-binding protein in goat colostrum may be related to its low sensitization. Cathelicidin-1 found in epithelial and neutrophils cells is an antimicrobial peptide and has profound impacts on wound healing, inflammation, and the regulation of adaptive immunity (36). The existence of antimicrobial proteins in the colostrum of goat and bovine milk explained that the protection from milk was necessary for newborn mammals and varies across species.

### DEMPs Analysis Based on Bioinformatics

Bioinformatics analysis showed that the response to dehydroepiandrosterone, 11-deoxycorticosterone, estradiol, and progesterone were the most abundant biological processes in colostrum and mature milk. Previous researches showed that the interaction between low-density lipoprotein and heparin results in irreversible structural changes in apolipoprotein B and affects low-density lipoprotein oxidation, phospholipolysis, and fusion (37). Dehydroepiandrosterone, 11-deoxycorticosterone, estradiol, and progesterone are endogenous steroid hormones, which are often conjugated with proteins, secreted by endocrine glands and endocrine cells, dispersed in other organs, and then transported to various organs through blood, provided with the function of coordinating and controlling tissue, organ metabolism, and physiological function (38). Progesterone is a good marker to determine the milk production function status, which may affect the milk production of goats and make it lower than that of bovines. Cholesterol-rich plaques accumulated in the arteries, preventing enough blood flow to the heart, causing cardiovascular disease, bring about many medical suggestions, and require a reduction of the cholesterol intake (39). However, throughout the lactation periods, the cholesterol levels in milk decreased sharply, and the cholesterol concentration in mature milk of goat (11.64 ± 1.09 mg/dL) was lower than that in bovine (20.58 ± 4.21 mg/dL), which is consistent with the results of the GO analysis of the DEMPs in mature milk of goat and bovine. The result of the most abundant biological processes in colostrum and mature milk indicated that goat milk is more comfortable for the human body. The extent of a triglyceride catabolic process, which occurs via acid lipolysis in the lysosome and neutral lipolysis in the cytoplasm, can regulate lipid droplet size (40). LPL and APOE as the critical enzymes that participate in the triglyceride catabolic process were higher in mature milk of goat than that in bovine goat milk, which confirmed that the regulating lipid metabolism in MFGM proteins of the goat was superior to that of bovine MFGM proteins.

The phagosome pathway is the important process of catabolism and transport, which has been reported in the Guanzhong goat and Holstein cow mature milk (16). Yang et al. (41) revealed that the phagosome pathway was a complicated process of organic uptake and elimination of apoptotic cells and pathogens, which contributes to inflammation, host defense, and homeostasis in tissues. Thus, the MFGM proteins may be crucial to the immune system of newborn mammals. The ECM-receptor interaction pathway has been found to be associated with depot-specific adipogenesis in cow and overrepresented in specific cattle breeds related to the adaptive immune response after virus inoculation in Holstein cattle (42). The structural functional diversity of proteoglycans makes them as key mediators of the interaction between tumor cells and host microenvironment, and directly participates in the tissue and dynamic remodeling of the milieu. As constituents of the ECM or extracellular milieu, proteoglycans may invariably participate in the control of a variety of oncogenic events in a multivalent manner (43). KEGG pathway analysis also displayed that the differential MFGM proteins in colostrum goat and bovine significantly regulated glycometabolism through the pathway of galactose metabolism and revealed that the MFGM proteins of goat and bovine milk possessed different effects on glucose metabolism. Peroxisome proliferator-activated receptors (PPARs) are the members of the nuclear hormone receptor superfamily, which has three member isotypes: PPARα, PPARβ/δ, and PPARγ, and is ligand-activated transcription factors. PPARs govern the expression of the crux molecules in fatty acid metabolic pathway, including the absorption, oxidation, and storage of fatty acids. Meanwhile, PPARγ maintains glucose homeostasis by activating glucose transporter 2 and glucokinase in the pancreas and liver (44). LPL, ACSL1, FABP3, and CD36 play key roles in the PPAR signaling pathway, and the abundance of these proteins in goat milk is higher than that in bovine milk, which indicated that goat milk has better function of fatty acid metabolism and glucose homeostasis. Due to the different functions of MFGM protein, the MFGM protein expressed in different lactation periods of bovine and goat provided significant information for functional food and infant formula. According to the nutritional needs of different lactating infants, functional proteins can be added to the corresponding formula, which is conducive to the health of infants.

In a previous study, the efficiency of preventing a series of enteric inflammatory and infectious diseases by milks has been documented. Viral myocarditis and salmonella infection are the subcategory of cardiovascular disease and infectious disease, respectively. The MFGM protein in mature milk significantly inhibited the internalization and binding of salmonella during the growth of mammals, and the inhibitory effect of goat milk was stronger than that of bovine milk, which was in line with our data (45). Sun et al. (15) reported that most of goat MFGM proteins were related to metabolism processes, including lipid metabolism and carbohydrate metabolism. According to the KEGG analysis results, the difference of fatty acid synthesis pathway between bovine and goat could mainly be a consequence of fatty acid synthetase activity differences between bovine and goat. Fatty acid synthases are located on the surface of endoplasmic reticulum and on the cytoplasmic lipid droplets in mammary epithelial cells. The endoplasmic reticulum possesses a series of membrane enzymes, such as palmitoyl-CoA and glycerol 3-phosphate, which can synthesize triglycerides from activated fatty acids. Palmitoyl-CoA, a major product of fatty acid synthases, and fatty acid synthases were directly connected in the fatty acid synthesis pathway (46). In addition, glucose and fructose promote the accumulation of triglycerides in milk to convert sugar to fat, the allosteric inhibition of fatty acid oxidation via increased availability of triose phosphate precursors and acetyl-CoA and metabolites, such as malonyl-CoA, for fatty acid formation via glycerol-3-phosphate biosynthesis and *de novo* lipogenesis (47). Regulation of fatty acid biosynthetic could inhibit hepatic steatosis and lipid accumulation.

## Conclusions

Our study described a more specific strategy to provide insights into proteome differences between GC, GM, BC, and BM. Bioinformatics analysis displayed that these DEMPs in colostrum significantly regulated glycometabolism through the pathway of galactose metabolism, and the DEMPs in mature milk regulated lipid metabolism through the pathway of fatty acid biosynthesis. These trials and results could reveal the differences of nutritional value and physiological states between bovine and goat at various lactation periods, and provide direction for the application of goat milk in infant formula food and functional food. Further study on the exact role of these significant difference proteins and the specific mechanism of activating the regulation pathway of galactose and lipid is necessary and could potentially help to determine the new biomarkers or establish the optimized formula of goat milk-based functional food.

## Data Availability Statement

The original contributions generated for the study are publicly available. This data can be found here: https://zenodo.org/record/4638812#.YHbYPOhKiUm.

## Author Contributions

WJ: conceptualization, methodology, software, writing- original draft preparation, and supervision. RZ: data curation and writing- original draft preparation. ZZ: reviewing and editing. LS: software. All authors contributed to the article and approved the submitted version.

## Conflict of Interest

The authors declare that the research was conducted in the absence of any commercial or financial relationships that could be construed as a potential conflict of interest.
