# Supplementary material for: A High-Throughput Comparative Proteomics of Milk Fat Globule Membrane Reveals Breed and Lactation Stages Specific Variation in Protein Abundance and Functional Differences Between Milk of Saanen Dairy Goat and Holstein Bovine
Source: Front Nutr. 2021 May 28;8:680683. doi: 10.3389/fnut.2021.680683 (PMC8193056; doi:10.3389/fnut.2021.680683)
Supplement: Supplementary file 1 [file Data_Sheet_1.docx]

**Supplementary Materials**

**A high-throughput comparative proteomics of milk fat globule membrane reveals breed and lactation stages specific variation in protein abundance and functional differences between milk of Saanen dairy goat and Holstein bovine**

Wei Jia ^a, *^, Rong Zhang ^a^, Zhenbao Zhu ^a^, Lin Shi ^a, *^ ^[[1]](#footnote-2)^

^a^*School of Food and Biological Engineering, Shaanxi University of Science & Technology, Xi'an 710021, China*

*** Corresponding author. Phone: +86-029-86168583, Fax: +86-029-86168583.**

***E-mail address:* jiawei@sust.edu.cn**


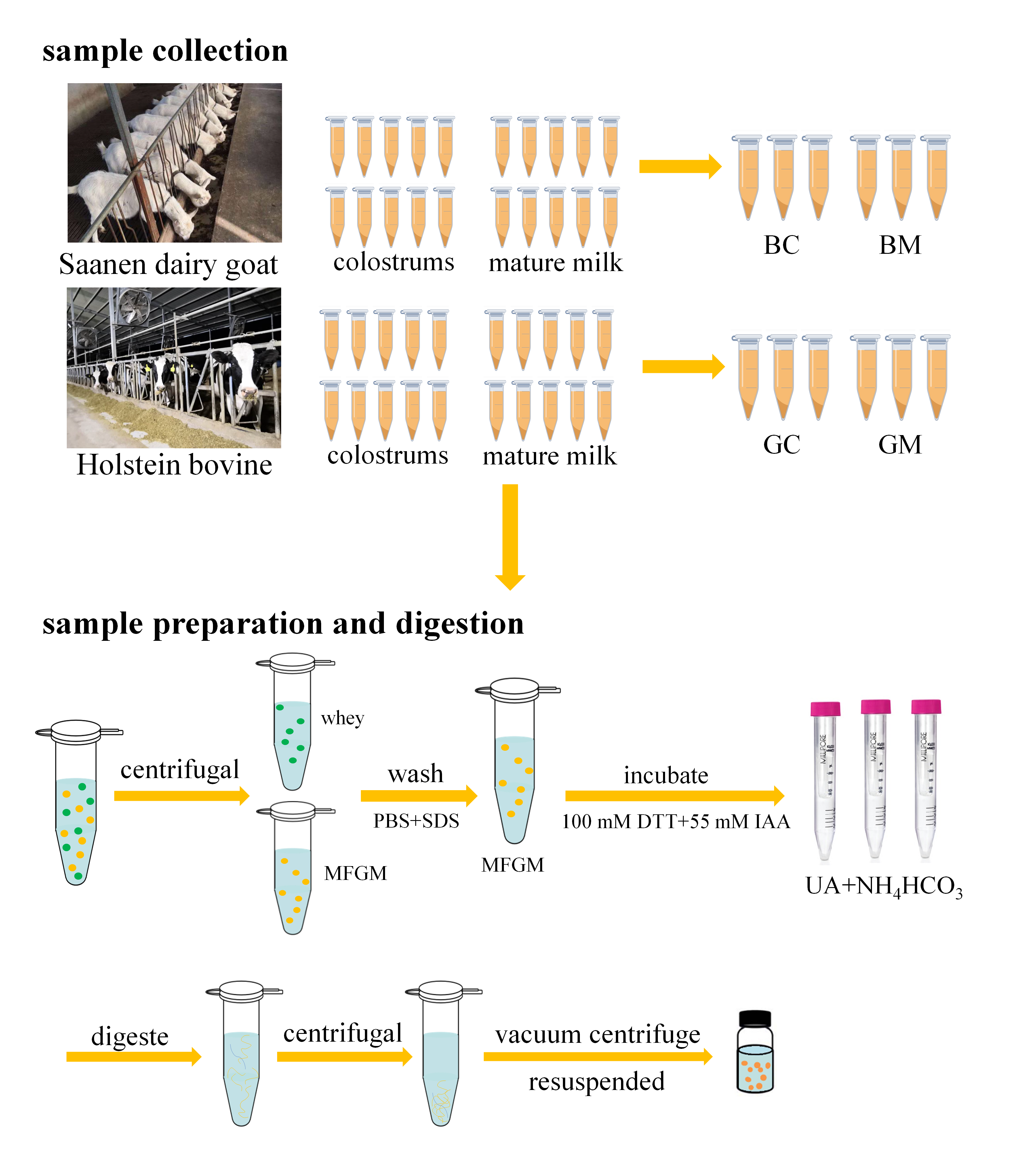


**Figure S1.** Diagram of sample collection and preparation.


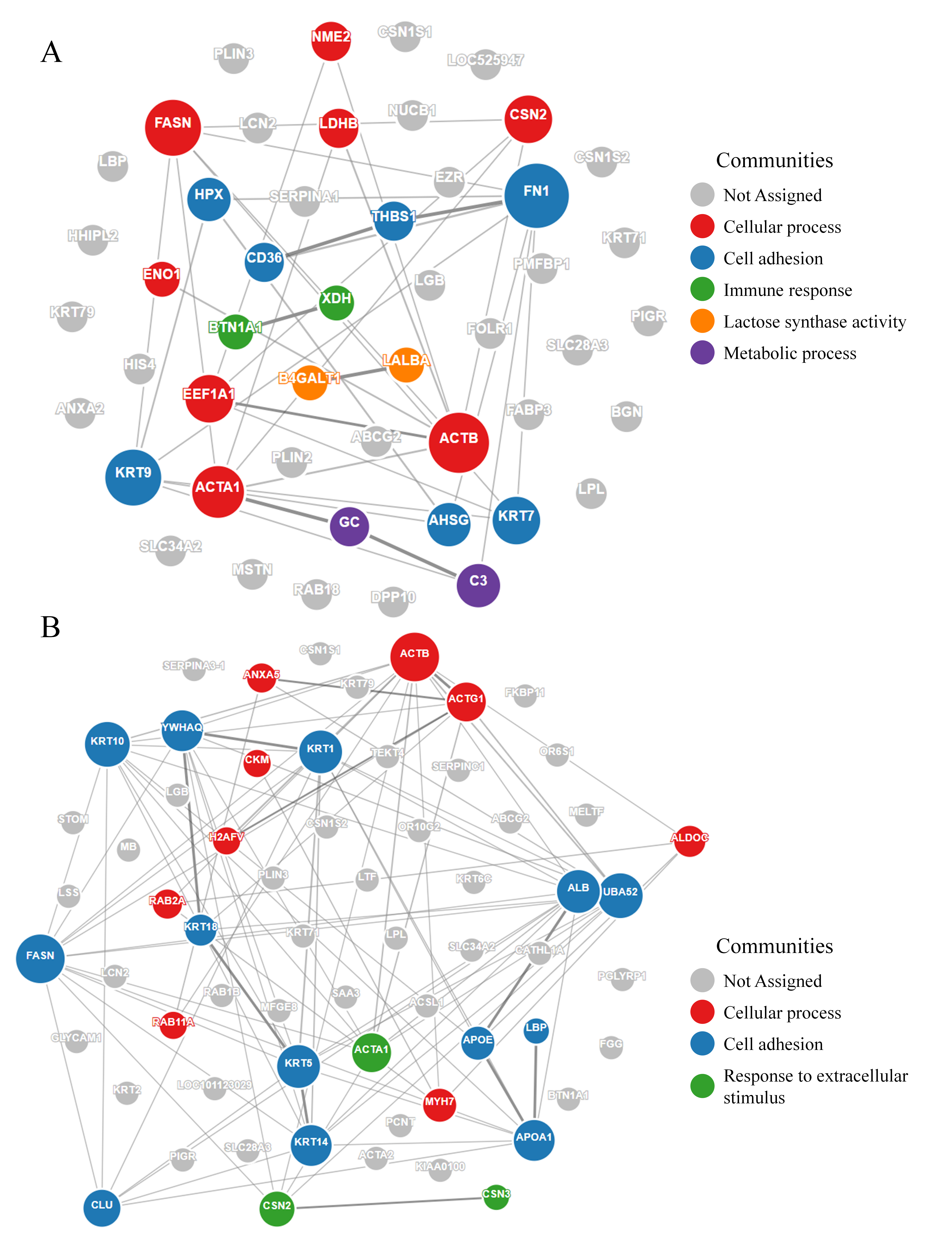


**Figure S2.** Protein-protein interaction analysis of DEMPs in colostrum of goat and bovine (A), in mature milk of goat and bovine (B).

**Table S1** Identified MFGM protein in goat colostrum, goat mature milk, bovine colostrum, bovine mature milk.

| Protein names | Gene names | Length | Peptides 1^1^ | Peptides 2^1^ | Peptides 3^1^ | Unique peptides1^2^ | Unique peptides2^2^ | Unique peptides3^2^ | LFQ intensity 1^3^ | LFQ intensity 2^3^ | LFQ intensity 3^3^ |
| --- | --- | --- | --- | --- | --- | --- | --- | --- | --- | --- | --- |
|  |  |  |  |  |  |  |  |  |  |  |  |
| GC |  |  |  |  |  |  |  |  |  |  |  |
| Cathelicidin | CATH2 | 176 | 2 | 2 | 2 | 2 | 2 | 2 | 145146 | 145450 | 141886 |
| ATP-binding cassette sub-family G member 2 | ABCG2 | 658 | 3 | 3 | 3 | 3 | 3 | 3 | 72309000 | 65683000 | 81543000 |
| Acetyl-CoA carboxylase 1 | ACACA | 2329 | 7 | 7 | 8 | 7 | 7 | 8 | 26357000 | 33961000 | 30739000 |
| Cytoplasmic aconitate hydratase | ACO1 | 889 | 5 | 5 | 5 | 5 | 5 | 5 | 22541000 | 26826000 | 20585000 |
| AMP-binding domain-containing protein | ACSL1 | 672 | 4 | 4 | 4 | 4 | 4 | 4 | 21752000 | 21671000 | 21692000 |
| Acyl-CoA synthetase long chain family member 3 | ACSL3 | 695 | 4 | 3 | 4 | 4 | 3 | 4 | 70966000 | 86680000 | 56856000 |
| Acyl-CoA synthetase short chain family member 2 | ACSS2 | 701 | 0 | 4 | 0 | 0 | 2 | 0 | 9764500 | 8140900 | 10202000 |
| Actin, alpha skeletal muscle | ACTA1 | 69 | 4 | 1 | 2 | 3 | 1 | 2 | 2808113 | 2049844 | 209980 |
| Actin, aortic smooth muscle | ACTA2 | 377 | 8 | 8 | 8 | 1 | 1 | 1 | 172960000 | 151640000 | 198230000 |
| Actin, cytoplasmic 1 | ACTB | 375 | 9 | 10 | 10 | 2 | 3 | 3 | 370830000 | 409790000 | 402670000 |
| Actin, alpha cardiac muscle 1 | ACTC1 | 374 | 3 | 3 | 3 | 3 | 3 | 3 | 33897000 | 50767000 | 43698000 |
| Alpha-actinin-4 | ACTN4 | 827 | 2 | 2 | 2 | 2 | 2 | 2 | 4813200 | 5097600 | 0 |
| Actin-related protein 2 | ACTR2 | 394 | 6 | 6 | 6 | 6 | 6 | 6 | 0 | 2410173 | 2647300 |
| ADAM metallopeptidase domain 18 | ADAM18 | 743 | 2 | 2 | 2 | 2 | 2 | 2 | 175851 | 188342 | 169502 |
| 1-acyl-sn-glycerol-3-phosphate acyltransferase | AGPAT1 | 287 | 5 | 6 | 5 | 5 | 6 | 5 | 5927215 | 5191433 | 5030700 |
| Alpha-2-HS-glycoprotein | AHSG | 359 | 4 | 4 | 0 | 2 | 2 | 0 | 3900253 | 3858574 | 4434900 |
| Serum albumin | ALB | 607 | 10 | 10 | 8 | 10 | 10 | 8 | 248240000 | 239790000 | 245540000 |
| Aldedh domain-containing protein | ALDH1A1 | 487 | 3 | 3 | 3 | 3 | 3 | 3 | 17898000 | 19343000 | 16546000 |
| Aldehyde dehydrogenase | ALDH3B1 | 468 | 5 | 4 | 5 | 5 | 4 | 5 | 11511000 | 5963200 | 7139600 |
| Fructose-bisphosphate aldolase | ALDOC | 364 | 21 | 22 | 22 | 21 | 22 | 22 | 0 | 4458456 | 4726800 |
| Annexin A1 | ANXA1 | 346 | 2 | 2 | 2 | 2 | 2 | 2 | 36892000 | 28947000 | 34209000 |
| Annexin A11 | ANXA11 | 503 | 6 | 6 | 5 | 6 | 6 | 5 | 183144 | 118731 | 121882 |
| Annexin A2 | ANXA2 | 339 | 2 | 2 | 2 | 2 | 2 | 2 | 26699000 | 18419000 | 20743000 |
| Annexin A5 | ANXA5 | 321 | 4 | 3 | 3 | 4 | 3 | 3 | 23712000 | 21518000 | 19568000 |
| Apolipoprotein A1 | APOA1 | 259 | 5 | 6 | 5 | 5 | 6 | 5 | 118440000 | 0 | 143530000 |
| Apolipoprotein E | APOE | 316 | 5 | 5 | 6 | 5 | 5 | 6 | 111500000 | 122550000 | 127740000 |
| Beta-2-glycoprotein 1 | APOH | 345 | 12 | 10 | 12 | 12 | 10 | 12 | 5287632 | 4903746 | 4347200 |
| Rho-GAP domain-containing protein | ARHGAP31 | 1440 | 2 | 2 | 2 | 2 | 2 | 2 | 20661000 | 20647414 | 0 |
| Rho GDP-dissociation inhibitor 1 | ARHGDIA | 204 | 1 | 4 | 1 | 1 | 2 | 1 | 10405547 | 11057380 | 11656000 |
| Adipose triglyceride lipase | ATGL | 486 | 2 | 2 | 2 | 2 | 2 | 2 | 9184500 | 7383900 | 7514200 |
| Cation-transporting ATPase | ATP13A4 | 1197 | 3 | 3 | 3 | 3 | 3 | 3 | 11643000 | 12630000 | 13307000 |
| ATP synthase subunit alpha, mitochondrial | ATP5F1A | 553 | 5 | 5 | 6 | 5 | 5 | 6 | 38307000 | 41703000 | 47309000 |
| ATP synthase subunit beta | ATP5F1B | 528 | 9 | 7 | 8 | 9 | 7 | 8 | 56465000 | 53879000 | 47925000 |
| CUE domain-containing protein | AUP1 | 410 | 7 | 2 | 1 | 5 | 2 | 1 | 0 | 7798845 | 7405100 |
| Beta-2-microglobulin | B2M | 118 | 3 | 3 | 3 | 3 | 3 | 3 | 30528000 | 29029000 | 47548000 |
| Beta-1,4-galactosyltransferase I | B4GALT1 | 402 | 4 | 4 | 4 | 4 | 4 | 4 | 41117000 | 36453000 | 27050000 |
| Biglycan | BGN | 369 | 4 | 4 | 4 | 4 | 4 | 4 | 0 | 3324405 | 3180500 |
| GLTSCR1 domain-containing protein | BICRA | 1467 | 7 | 7 | 7 | 7 | 7 | 7 | 146803 | 117096 | 133035 |
| Bactericidal permeability-increasing protein | BPI | 462 | 6 | 5 | 5 | 6 | 5 | 5 | 0 | 199477 | 144235 |
| BPI1 domain-containing protein | BPIFB1 | 514 | 14 | 14 | 13 | 14 | 14 | 13 | 27800000 | 10004000 | 4484000 |
| Butyrophilin subfamily 1 member A1 | BTN1A1 | 526 | 10 | 10 | 10 | 10 | 10 | 10 | 359250000 | 353210000 | 274050000 |
| Complement C3 | C3 | 1661 | 6 | 5 | 6 | 0 | 0 | 0 | 150460000 | 160200000 | 150150000 |
| C4b-binding protein alpha chain | C4BPA | 599 | 4 | 1 | 2 | 2 | 1 | 2 | 4217958 | 4755294 | 4480900 |
| Carbamoyl-phosphate synthetase 2, aspartate transcarbamylase, and dihydroorotase | CAD | 2162 | 2 | 2 | 2 | 2 | 2 | 2 | 18538000 | 15974000 | 15675000 |
| Calcium homeostasis modulator 1 | CALHM1 | 339 | 0 | 0 | 1 | 0 | 0 | 1 | 0 | 109444 | 119820 |
| Calreticulin | CALR | 417 | 3 | 3 | 3 | 3 | 3 | 3 | 12117000 | 13735000 | 13619000 |
| Adenylyl cyclase-associated protein | CAP1 | 454 | 18 | 20 | 19 | 18 | 20 | 19 | 2221623 | 3025947 | 2293700 |
| F-actin-capping protein subunit beta | CAPZB | 110 | 20 | 20 | 20 | 1 | 2 | 2 | 3491068 |  | 3030700 |
| Capping protein regulator and myosin 1 linker 1 | CARMIL1 | 1333 | 1 | 2 | 2 | 1 | 2 | 2 | 5707793 | 6111000 | 5213900 |
| Coiled-coil domain containing 18 | CCDC18 | 1453 | 6 | 6 | 6 | 0 | 0 | 0 | 13748591 | 14329516 | 155280000 |
| CCN family member 2 | CCN2 | 349 | 23 | 23 | 23 | 23 | 23 | 23 | 0 | 124420 | 126304 |
| T-complex protein 1 subunit gamma | CCT3 | 545 | 2 | 2 | 2 | 2 | 2 | 2 | 8371600 | 6127800 | 7700400 |
| T-complex protein 1 subunit delta | CCT4 | 502 | 7 | 7 | 7 | 7 | 7 | 7 | 0 | 4133931 | 4489400 |
| Chaperonin containing TCP1 subunit 5 | CCT5 | 541 | 2 | 2 | 2 | 2 | 2 | 2 | 2411900 | 2558400 | 1996300 |
| T-complex protein 1 subunit zeta | CCT6A | 490 | 1 | 2 | 2 | 1 | 2 | 2 | 3123885 | 3230349 | 3371800 |
| T-complex protein 1 subunit theta | CCT8 | 548 | 3 | 3 | 3 | 3 | 3 | 3 | 16587000 | 14134000 | 16852000 |
| Monocyte differentiation antigen CD14 | CD14 | 371 | 4 | 3 | 3 | 3 | 2 | 2 | 6323448 | 5607325 | 5727200 |
| CD36 | CD36 | 472 | 5 | 5 | 5 | 5 | 5 | 5 | 329750000 | 377670000 | 326180000 |
| Leukocyte surface antigen CD47 | CD47 | 267 | 4 | 5 | 4 | 4 | 5 | 4 | 0 | 107614 | 101562 |
| Cofilin-1 | CFL1 | 165 | 6 | 6 | 6 | 6 | 6 | 6 | 13438105 | 15441000 | 22569000 |
| Chromodomain helicase DNA binding protein 3 | CHD3 | 1972 | 6 | 6 | 6 | 6 | 6 | 6 | 8057337 | 7839243 | 7029000 |
| Chitinase-3-like protein 1 | CHI3L1 | 383 | 5 | 5 | 5 | 5 | 5 | 5 | 6387656 | 0 | 6217800 |
| Calcineurin B homologous protein 1 | CHP1 | 195 | 2 | 2 | 2 | 2 | 2 | 2 | 15770000 | 16336000 | 14825000 |
| Chordin like 2 | CHRDL2 | 425 | 10 | 11 | 10 | 10 | 11 | 10 | 101570 | 106172 | 101274 |
| Cell death-inducing DFFA-like effector a | CIDEA | 202 | 5 | 5 | 5 | 5 | 5 | 5 | 104570000 | 122040000 | 91170000 |
| Chloride intracellular channel protein | CLIC4 | 241 | 3 | 3 | 3 | 3 | 3 | 3 | 0 | 3342170 | 3453700 |
| Clathrin heavy chain | CLTC | 1644 | 2 | 2 | 2 | 2 | 2 | 2 | 8352000 | 8198600 | 8645500 |
| Clusterin | CLU | 439 | 5 | 5 | 5 | 5 | 5 | 5 | 66596000 | 79298000 | 81038000 |
| M20_dimer domain-containing protein | CNDP2 | 457 | 2 | 2 | 2 | 2 | 2 | 2 | 145431 | 0 | 143891 |
| Saposin B-type domain-containing protein | CNPY2 | 182 | 2 | 2 | 2 | 2 | 2 | 2 | 6044084 | 6884700 | 8021800 |
| Catechol O-methyltransferase | COMT | 272 | 2 | 2 | 2 | 2 | 2 | 2 | 3021032 | 2980000 | 3292700 |
| Coatomer subunit alpha | COPA | 1224 | 0 | 7 | 4 | 0 | 2 | 0 | 0 | 109000 | 111115 |
| Crooked neck pre-mRNA splicing factor 1 | CRNKL1 | 693 | 0 | 4 | 8 | 0 | 3 | 3 | 108081 | 0 | 109600 |
| Pentaxin | CRP | 221 | 6 | 6 | 6 | 6 | 6 | 6 | 2640146 | 2274514 | 2178000 |
| Zeta-crystallin | CRYZ | 330 | 3 | 3 | 3 | 3 | 3 | 3 | 0 | 13268721 | 12661000 |
| Alpha s1 casein | CSN1S1 | 214 | 11 | 12 | 12 | 5 | 5 | 5 | 4921900000 | 5774500000 | 5249700000 |
| Alpha-S2-casein | CSN1S2 | 223 | 14 | 15 | 14 | 14 | 15 | 14 | 1005200000 | 1014800000 | 1137500000 |
| Beta-casein | CSN2 | 222 | 6 | 6 | 6 | 5 | 5 | 5 | 2508800000 | 3303500000 | 2271700000 |
| Kappa casein | CSN3 | 141 | 4 | 4 | 4 | 2 | 2 | 2 | 319130000 | 259060000 | 240380000 |
| Cullin 7 | CUL7 | 1683 | 2 | 2 | 2 | 2 | 2 | 2 | 144120 | 0 | 147863 |
| Homeobox protein cut-like | CUX1 | 1534 | 4 | 4 | 4 | 4 | 4 | 4 | 9625880 | 9853593 | 9540700 |
| Dynactin subunit 2 | DCTN2 | 406 | 2 | 2 | 1 | 2 | 2 | 1 | 7089600 | 5419000 | 0 |
| Dehydrogenase/reductase 1 | DHRS1 | 313 | 4 | 4 | 4 | 4 | 4 | 4 | 46222000 | 58333000 | 68338000 |
| Dynein axonemal heavy chain 7 | DNAH7 | 4016 | 5 | 6 | 6 | 5 | 6 | 6 | 0 | 34038920 | 38918000 |
| Chaperone protein DnaJ | dnaJ | 376 | 2 | 1 | 2 | 2 | 1 | 2 | 114290748 | 118520687 | 119370000 |
| J domain-containing protein | DNAJC5 | 174 | 2 | 1 | 1 | 2 | 1 | 1 | 0 | 1468665 | 1411500 |
| Dipeptidyl-peptidase 10 | DPP10 | 789 | 8 | 8 | 7 | 8 | 8 | 7 | 0 | 19151 | 17816 |
| Dipeptidyl peptidase 4 | DPP4 | 762 | 2 | 2 | 1 | 2 | 2 | 1 | 8234700 | 8095200 | 8675932 |
| SH3 domain-containing protein | DSP | 2748 | 2 | 2 | 1 | 2 | 2 | 1 | 6676500 | 5922800 | 0 |
| ADF-H domain-containing protein | DSTN | 165 | 2 | 2 | 2 | 2 | 2 | 2 | 3137800 | 4159600 | 4341600 |
| Dynein cytoplasmic 1 heavy chain 1 | DYNC1H1 | 4619 | 1 | 2 | 1 | 1 | 2 | 1 | 0 | 2522145 | 2268900 |
| Elongation factor 1-alpha 1 | EEF1A1 | 462 | 6 | 7 | 7 | 3 | 4 | 4 | 178990000 | 227360000 | 189530000 |
| Elongation factor 1-delta | EEF1D | 75 | 3 | 3 | 3 | 3 | 3 | 3 | 1732390 | 1768941 | 1926500 |
| Tr-type G domain-containing protein | EEF2 | 858 | 4 | 4 | 3 | 4 | 4 | 3 | 31882000 | 35263000 | 24016000 |
| EH domain-containing protein 1 | EHD1 | 489 | 2 | 1 | 2 | 2 | 1 | 2 | 4206302 | 3775712 | 3867100 |
| EH domain containing 4 | EHD4 | 540 | 3 | 3 | 3 | 3 | 3 | 3 | 11610000 | 13664000 | 11132000 |
| S1 motif domain-containing protein | EIF2S1 | 315 | 20 | 20 | 20 | 2 | 2 | 2 | 84175 | 0 | 73762 |
| Eukaryotic translation initiation factor 2 subunit 3 structural gene Y-linked | Eif2s3y | 470 | 2 | 2 | 2 | 2 | 2 | 2 | 1809527 | 1848048 | 1702200 |
| Eukaryotic translation initiation factor 3 subunit A | EIF3A | 1332 | 1 | 2 | 1 | 1 | 2 | 1 | 2052004 | 3566389 | 2561000 |
| Eukaryotic translation initiation factor 3 subunit M | EIF3M | 374 | 3 | 2 | 3 | 3 | 2 | 3 | 0 | 2666930 | 2195500 |
| Eukaryotic initiation factor 4A-I | EIF4A1 | 406 | 4 | 4 | 4 | 2 | 2 | 2 | 23367000 | 23693000 | 29754000 |
| Eukaryotic translation initiation factor 5A | EIF5A2 | 142 | 2 | 3 | 3 | 2 | 3 | 3 | 8424462 | 7683389 | 8342800 |
| Alpha-enolase | ENO1 | 434 | 5 | 6 | 6 | 5 | 6 | 6 | 0 | 2561847 | 2854300 |
| Glutamyl-prolyl-tRNA synthetase | EPRS | 1511 | 3 | 3 | 3 | 3 | 3 | 3 | 10686000 | 11476000 | 10656000 |
| ERO1-like protein alpha | ERO1A | 471 | 10 | 11 | 11 | 2 | 5 | 2 | 129171 | 0 | 122952 |
| Endoplasmic reticulum protein 29 | ERP29 | 259 | 2 | 1 | 1 | 2 | 1 | 1 | 4163957 | 4486165 | 4643600 |
| Ezrin | EZR | 581 | 3 | 2 | 3 | 1 | 1 | 1 | 55977000 | 58114000 | 60563000 |
| Prothrombin | F2 | 612 | 3 | 2 | 3 | 3 | 2 | 3 | 117010 | 0 | 141260 |
| FABP domain-containing protein | FABP3 | 133 | 5 | 5 | 5 | 5 | 5 | 5 | 34446000 | 39060000 | 33831000 |
| Fatty acid synthase | FASN | 2510 | 32 | 33 | 34 | 32 | 33 | 34 | 1248000000 | 1204300000 | 1192300000 |
| Fasciculation and elongation protein zeta 2 | FEZ2 | 375 | 4 | 4 | 4 | 4 | 4 | 4 | 0 | 108495 | 10403 |
| Mitochondrial fission 1 protein | FIS1 | 152 | 1 | 2 | 1 | 1 | 2 | 1 | 3477293 | 3789442 | 3262200 |
| Peptidylprolyl isomerase | FKBP11 | 191 | 4 | 4 | 4 | 2 | 2 | 0 | 88187 | 0 | 95565 |
| Fibronectin | FN1 | 2137 | 4 | 4 | 3 | 4 | 4 | 3 | 5203892 | 4582222 | 4439000 |
| Folate receptor alpha | FOLR1 | 251 | 3 | 3 | 3 | 3 | 3 | 3 | 10213000 | 14021000 | 9073100 |
| Folate receptor gamma | FOLR3 | 258 | 4 | 4 | 4 | 4 | 4 | 4 | 32104000 | 32998000 | 42441000 |
| Acidic amino acid decarboxylase GADL1 | GADL1 | 449 | 1 | 2 | 1 | 1 | 2 | 1 | 0 | 6416704 | 6701200 |
| NAD | GALE | 348 | 2 | 2 | 2 | 2 | 2 | 0 | 4351295 | 3552999 | 3687500 |
| Gal_mutarotas_2 domain-containing protein | GANAB | 869 | 3 | 3 | 3 | 3 | 3 | 3 | 15460000 | 20124000 | 14410000 |
| AA_TRNA_LIGASE_II domain-containing protein | GARS | 616 | 1 | 2 | 2 | 1 | 2 | 2 | 127120 | 0 | 162522 |
| Vitamin D-binding protein | GC | 468 | 5 | 6 | 5 | 5 | 6 | 5 | 49657000 | 48627000 | 51774000 |
| Rab GDP dissociation inhibitor | GDI2 | 430 | 2 | 2 | 2 | 2 | 2 | 2 | 8738300 | 9667000 | 8561500 |
| Guanine nucleotide binding protein alpha inhibiting 2 | GI2 | 306 | 2 | 3 | 2 | 1 | 2 | 1 | 8081729 | 8098795 | 7908800 |
| Glutaredoxin-3 | GLRX3 | 335 | 5 | 5 | 5 | 5 | 5 | 5 | 7168834 | 0 | 7515500 |
| GLTP domain-containing protein | GLTP | 209 | 9 | 8 | 9 | 9 | 8 | 9 | 1712300 | 1429668 | 1619311 |
| Glycosylation-dependent cell adhesion molecule 1 | GLYCAM1 | 152 | 2 | 2 | 2 | 2 | 2 | 2 | 294870000 | 321020000 | 350680000 |
| Guanine nucleotide-binding protein subunit alpha-11 | GNA11 | 314 | 3 | 2 | 2 | 2 | 1 | 1 | 23411000 | 22312000 | 21062000 |
| Guanine nucleotide-binding protein subunit alpha-13 | GNA13 | 294 | 2 | 2 | 2 | 2 | 2 | 2 | 6990400 | 7132800 | 0 |
| Guanine nucleotide-binding protein G(o) subunit alpha | GNAO1 | 354 | 2 | 3 | 2 | 1 | 2 | 1 | 25424000 | 34066000 | 46191000 |
| Guanine nucleotide-binding protein G(I)/G(S)/G(T) subunit beta-1 | GNB1 | 325 | 3 | 3 | 3 | 2 | 2 | 2 | 31613000 | 25149000 | 20213000 |
| Guanine nucleotide-binding protein G(I)/G(S)/G(T) subunit beta-2 | GNB2 | 340 | 3 | 3 | 3 | 2 | 2 | 2 | 21206000 | 18840000 | 15744000 |
| Guanine nucleotide-binding protein beta polypeptide 2-like 1 | GNB2L1 | 317 | 2 | 1 | 2 | 2 | 1 | 2 | 7831634 | 7333900 | 0 |
| Guanine nucleotide-binding protein subunit gamma | GNG12 | 72 | 3 | 3 | 3 | 3 | 3 | 3 | 5477959 | 6267341 | 6133200 |
| Gse1 coiled-coil protein | GSE1 | 1191 | 5 | 5 | 5 | 5 | 5 | 5 | 136930 | 0 | 177920 |
| Gelsolin | GSN | 780 | 7 | 8 | 6 | 7 | 8 | 6 | 91509000 | 67875000 | 71193000 |
| Histone H2A.J | H2AFJ | 129 | 3 | 3 | 3 | 3 | 3 | 3 | 53680000 | 54753000 | 57689000 |
| Hemoglobin subunit beta-C | HBBC | 141 | 3 | 4 | 3 | 0 | 2 | 0 | 4152172 | 4794811 | 4204300 |
| Haloacid dehalogenase like hydrolase domain containing 5 | HDHD5 | 390 | 10 | 9 | 8 | 10 | 9 | 8 | 8001690 | 0 | 8453400 |
| Heme-binding protein 1 | HEBP1 | 191 | 4 | 4 | 4 | 4 | 4 | 4 | 5024204 | 5429447 | 5447300 |
| HHIP like 2 | HHIPL2 | 727 | 4 | 3 | 3 | 4 | 3 | 3 | 0 | 122210 | 115090 |
| Hemopexin | HPX | 459 | 2 | 2 | 2 | 2 | 2 | 2 | 3069448 | 2273025 | 2746700 |
| HSP27 protein | HSP27 | 183 | 2 | 2 | 2 | 2 | 2 | 2 | 14355000 | 18138000 | 13161000 |
| Heat shock protein HSP 90-beta | HSP90AB1 | 682 | 7 | 7 | 7 | 5 | 5 | 5 | 170460000 | 161540000 | 125460000 |
| Endoplasmin | HSP90B1 | 804 | 5 | 5 | 5 | 5 | 5 | 5 | 56849000 | 65089000 | 61454000 |
| Heat shock 70kDa protein 5 | HSPA5 | 655 | 6 | 7 | 5 | 6 | 7 | 5 | 100870000 | 113940000 | 81359000 |
| Heat shock cognate 71 kDa protein | HSPA8 | 590 | 3 | 4 | 4 | 3 | 4 | 4 | 73009000 | 66988000 | 60849000 |
| Stress-70 protein, mitochondrial | HSPA9 | 679 | 3 | 2 | 2 | 3 | 2 | 2 | 0 | 2842096 | 2214200 |
| Heat shock protein alpha | HSPCA | 733 | 5 | 5 | 5 | 3 | 3 | 3 | 32009000 | 36418000 | 40527000 |
| Hypoxia up-regulated 1 | HYOU1 | 1001 | 2 | 3 | 3 | 2 | 3 | 3 | 0 | 7774300 | 8369000 |
| Isocitrate dehydrogenase | IDH1 | 414 | 2 | 2 | 2 | 2 | 2 | 2 | 4643100 | 5215500 | 6061900 |
| Immunoglobulin heavy constant mu | IGHM | 456 | 4 | 4 | 3 | 2 | 2 | 2 | 123500000 | 150140000 | 121900000 |
| IQ motif containing GTPase activating protein 1 | IQGAP1 | 1657 | 5 | 4 | 5 | 5 | 4 | 5 | 9976700 | 10659000 | 9454100 |
| VWFA domain-containing protein | ITGAM | 1152 | 3 | 3 | 3 | 3 | 3 | 3 | 126530 | 0 | 111610 |
| Immunoglobulin J chain | JCHAIN | 158 | 2 | 2 | 2 | 2 | 2 | 2 | 4042367 | 4898600 | 4644229 |
| Lysine--tRNA ligase | KARS | 570 | 2 | 2 | 2 | 2 | 2 | 2 | 2389000 | 2348400 | 2880900 |
| BTB domain-containing protein | KCNS1 | 524 | 21 | 22 | 23 | 21 | 22 | 23 | 121820 | 131710 | 0 |
| Protein KIAA0100 | KIAA0100 | 2213 | 3 | 3 | 4 | 3 | 3 | 4 | 102519 | 58420 | 70018 |
| Keratin, type II cytoskeletal 1 | KRT1 | 644 | 16 | 16 | 16 | 12 | 12 | 12 | 1311800000 | 1048900000 | 1365700000 |
| Keratin, type I cytoskeletal 10 | KRT10 | 584 | 12 | 13 | 12 | 10 | 11 | 10 | 392970000 | 439410000 | 398890000 |
| Keratin, type I cytoskeletal 14 | KRT14 | 472 | 2 | 2 | 1 | 2 | 2 | 1 | 1280768 | 1331732 | 1382500 |
| Keratin, type I cytoskeletal 16 | KRT16 | 473 | 5 | 5 | 4 | 2 | 2 | 1 | 16085000 | 12243000 | 12627000 |
| Keratin, type I cytoskeletal 17 | KRT17 | 441 | 2 | 2 | 1 | 2 | 2 | 1 | 17997000 | 15654323 | 0 |
| Keratin, type II cytoskeletal 5 | KRT5 | 590 | 6 | 5 | 5 | 4 | 4 | 2 | 22150000 | 20082000 | 21626000 |
| Keratin, type II cytoskeletal 6A | KRT6A | 564 | 6 | 6 | 6 | 3 | 3 | 3 | 14647000 | 13424000 | 13603000 |
| Keratin, type II cytoskeletal 6C | KRT6C | 564 | 1 | 2 | 2 | 1 | 2 | 2 | 3239112 | 3457294 | 2957100 |
| Keratin, type II cytoskeletal 7 | KRT7 | 452 | 3 | 2 | 2 | 2 | 1 | 1 | 1543000 | 1414998 | 0 |
| Keratin type II cytoskeletal 71 | KRT71 | 368 | 4 | 3 | 3 | 1 | 2 | 1 | 8591594 | 8042800 | 7977400 |
| Keratin, type II cytoskeletal 75 | KRT75 | 495 | 4 | 4 | 4 | 3 | 3 | 3 | 73344000 | 74222000 | 70163000 |
| Keratin, type II cytoskeletal 79 | KRT79 | 535 | 16 | 16 | 15 | 10 | 10 | 9 | 309030000 | 376880000 | 367800000 |
| Keratin, type I cytoskeletal 9 | KRT9 | 623 | 16 | 16 | 17 | 15 | 15 | 16 | 743610000 | 885900000 | 985470000 |
| Alpha-lactalbumin | LALBA | 142 | 8 | 8 | 8 | 5 | 5 | 5 | 1380200000 | 1431400000 | 1212400000 |
| LIM and SH3 domain protein 1 | LASP1 | 260 | 8 | 8 | 7 | 2 | 2 | 1 | 3761528 | 0 | 3424600 |
| Lipopolysaccharide-binding protein | LBP | 481 | 13 | 14 | 14 | 13 | 14 | 14 | 0 | 1808604 | 1780800 |
| Lipocln_cytosolic_FA-bd_dom domain-containing protein | LCN2 | 200 | 20 | 20 | 20 | 20 | 20 | 20 | 6545400 | 8646900 | 7263500 |
| L-lactate dehydrogenase | LDHB | 334 | 2 | 2 | 2 | 2 | 2 | 2 | 5039000 | 3994100 | 4178400 |
| Beta-lactoglobulin | LGB | 180 | 8 | 9 | 7 | 3 | 3 | 3 | 15282000000 | 13451000000 | 12045000000 |
| LIM domain kinase 2 | LIMK2 | 612 | 5 | 5 | 5 | 1 | 2 | 2 | 5729787 | 0 | 5920500 |
| PLAT domain-containing protein | LIPG | 500 | 15 | 14 | 13 | 15 | 14 | 13 | 2855870 | 2635865 | 2867200 |
| Histone H4 | HIS4 | 103 | 3 | 3 | 3 | 3 | 3 | 3 | 78600000 | 86855000 | 63826000 |
| Ribosomal protein L15 | LOC101109701 | 204 | 2 | 2 | 2 | 2 | 2 | 2 | 5475800 | 7509200 | 8228400 |
| Peptidyl-prolyl cis-trans isomerase NIMA-interacting 4 | PIN4 | 131 | 2 | 2 | 2 | 2 | 2 | 2 | 24408000 | 23559000 | 31166000 |
| Cystatin domain-containing protein | LOC101123265 | 98 | 10 | 8 | 10 | 10 | 8 | 10 | 0 | 110544 | 112400 |
| Methyltransf_11 domain-containing protein | LOC102168424 | 244 | 2 | 2 | 2 | 2 | 2 | 2 | 14367000 | 15482000 | 11993000 |
| NADH-cytochrome b5 reductase | LOC102173645 | 295 | 3 | 3 | 2 | 3 | 3 | 2 | 53523000 | 73243000 | 65233000 |
| Tubulin alpha chain | LOC102178426 | 433 | 2 | 2 | 2 | 1 | 1 | 1 | 29424000 | 28795000 | 32009000 |
| Ig-like domain-containing protein | SCN2B | 265 | 1 | 1 | 2 | 1 | 1 | 2 | 3824406 | 0 | 3872800 |
| S5 DRBM domain-containing protein | LOC108634081 | 293 | 2 | 2 | 2 | 2 | 2 | 2 | 7088400 | 6957100 | 0 |
| Serotransferrin-like | LOC525947 | 622 | 6 | 7 | 7 | 6 | 7 | 7 | 0 | 83825 | 54541 |
| Lipoprotein lipase | LPL | 460 | 2 | 2 | 2 | 2 | 2 | 2 | 40989000 | 32354000 | 38640000 |
| LRAT domain-containing protein | LRATD2 | 288 | 8 | 7 | 7 | 8 | 7 | 7 | 2206837 | 2078292 | 1997500 |
| Leucine rich repeat containing 24 | LRRC24 | 440 | 10 | 10 | 10 | 8 | 8 | 8 | 0 | 118718530 | 115190000 |
| LRRC57 protein | LRRC57 | 239 | 0 | 1 | 2 | 0 | 1 | 2 | 4201670 | 4095972 | 4283600 |
| U6 snRNA-associated Sm-like protein LSm4 | LSM4 | 139 | 1 | 2 | 1 | 1 | 2 | 1 | 0 | 163809100 | 186550000 |
| Lanosterol synthase | LSS | 732 | 3 | 2 | 3 | 3 | 2 | 3 | 36004000 | 29622000 | 33835000 |
| Lactotransferrin | LTF | 708 | 5 | 5 | 5 | 5 | 5 | 5 | 31042000 | 31402000 | 24592000 |
| T-lymphocyte surface antigen Ly-9 | LY9 | 338 | 4 | 0 | 4 | 3 | 0 | 4 | 118452 | 0 | 119200 |
| MAP28 protein | map28 | 158 | 4 | 4 | 3 | 4 | 4 | 3 | 0 | 317170 | 248197 |
| MAP34-A protein | map34-A | 165 | 13 | 13 | 13 | 13 | 13 | 13 | 54413460 | 53639100 | 59611000 |
| Methionine--tRNA ligase, cytoplasmic | MARS1 | 893 | 2 | 2 | 2 | 2 | 2 | 2 | 544576 | 0 | 418921 |
| Melanotransferrin | MELTF | 739 | 5 | 5 | 3 | 5 | 5 | 3 | 62696000 | 49524000 | 57796000 |
| Lactadherin | MFGE8 | 398 | 11 | 11 | 11 | 11 | 11 | 11 | 2915500000 | 2692800000 | 3040100000 |
| Macrophage migration inhibitory factor | MIF | 115 | 2 | 1 | 2 | 2 | 1 | 2 | 2955335 | 2424172 | 2750300 |
| Myeloperoxidase | MPO | 719 | 7 | 7 | 7 | 7 | 7 | 7 | 0 | 5055556 | 5668200 |
| Moesin | MSN | 562 | 3 | 2 | 3 | 1 | 1 | 1 | 7581900 | 5835100 | 7021600 |
| Myostatin | MSTN | 124 | 4 | 1 | 0 | 2 | 1 | 0 | 0 | 137481 | 190620 |
| Major vault protein | MVP | 850 | 3 | 3 | 3 | 3 | 3 | 3 | 13025000 | 12916000 | 10438000 |
| Myosin heavy chain 9 | MYH9 | 1965 | 6 | 5 | 6 | 6 | 5 | 6 | 25450000 | 21845000 | 21802000 |
| Myosin regulatory light chain 12B | MYL12B | 171 | 5 | 6 | 6 | 2 | 2 | 2 | 0 | 3601423 | 3297900 |
| Probable nicotinate-nucleotide adenylyltransferase | nadD | 367 | 5 | 5 | 4 | 1 | 1 | 0 | 7583400 | 6912770 | 7644082 |
| Protein NDRG1 | NDRG1 | 384 | 2 | 2 | 2 | 2 | 2 | 2 | 0 | 3612008 | 2839300 |
| Eukaryotic translation initiation factor 3 subunit C | NIP1 | 899 | 3 | 4 | 4 | 3 | 4 | 4 | 85249 | 98319 | 70984 |
| Nucleoside diphosphate kinase | NME2 | 152 | 2 | 1 | 1 | 2 | 1 | 1 | 5286240 | 5045903 | 5354200 |
| TPR_REGION domain-containing protein | NOXA1 | 238 | 4 | 5 | 5 | 4 | 5 | 5 | 4983030 | 0 | 50463000 |
| 3Beta_HSD domain-containing protein | NSDHL | 347 | 2 | 1 | 1 | 2 | 1 | 1 | 14323000 | 13642291 | 14857286 |
| Nucleobindin-1 | NUCB1 | 460 | 2 | 2 | 2 | 2 | 2 | 2 | 8917600 | 9611600 | 7325800 |
| Olfactomedin-like domain-containing protein | OLFML3 | 406 | 4 | 3 | 4 | 4 | 3 | 4 | 0 | 3962530 | 4667300 |
| G_PROTEIN_RECEP_F1_2 domain-containing protein | OR10G2 | 318 | 4 | 3 | 4 | 4 | 3 | 4 | 68650560 | 66980890 | 62441000 |
| P2X purinoceptor | P2RX3 | 397 | 13 | 11 | 13 | 13 | 11 | 13 | 57643820 | 0 | 59501000 |
| Protein disulfide-isomerase | P4HB | 507 | 7 | 6 | 7 | 7 | 6 | 7 | 54012000 | 51364000 | 51322000 |
| PACT_coil_coil domain-containing protein | PCNT | 3289 | 11 | 10 | 11 | 11 | 10 | 11 | 0 | 371604900 | 349420000 |
| BRO1 domain-containing protein | PDCD6IP | 875 | 0 | 2 | 0 | 0 | 0 | 2 | 1846074 | 0 | 1884000 |
| Protein disulfide-isomerase A3 | PDIA3 | 449 | 4 | 3 | 3 | 4 | 3 | 3 | 25219000 | 30582000 | 25040000 |
| Protein disulfide-isomerase A4 | PDIA4 | 644 | 5 | 5 | 5 | 5 | 5 | 5 | 20892000 | 22710000 | 22432000 |
| Protein disulfide isomerase family A member 6 | PDIA6 | 440 | 5 | 4 | 5 | 5 | 4 | 5 | 24237000 | 22593000 | 21651000 |
| 6-phosphofructokinase | PFKL | 750 | 1 | 1 | 2 | 1 | 1 | 2 | 0 | 1432278 | 1597500 |
| Profilin-1 | PFN1 | 140 | 2 | 2 | 2 | 2 | 2 | 2 | 13812000 | 14641000 | 13592000 |
| 6-phosphogluconate dehydrogenase, decarboxylating | PGD | 473 | 2 | 1 | 2 | 2 | 1 | 2 | 2809100 | 2947380 | 0 |
| Phosphoglycerate kinase | PGK1 | 415 | 2 | 2 | 2 | 2 | 2 | 2 | 5262700 | 4138600 | 4853700 |
| Peptidoglycan-recognition protein | PGLYRP1 | 190 | 1 | 4 | 1 | 1 | 2 | 1 | 7961356 | 7070935 | 7965700 |
| Polymeric immunoglobulin receptor | PIGR | 758 | 3 | 3 | 2 | 3 | 3 | 2 | 9139700 | 10288000 | 13617000 |
| Pyruvate kinase | PKM | 531 | 2 | 1 | 2 | 2 | 1 | 2 | 4737500 | 0 | 4234700 |
| Plasminogen | PLG | 812 | 3 | 4 | 4 | 3 | 4 | 4 | 0 | 113438 | 177580 |
| Perilipin-2 | PLIN2 | 378 | 10 | 11 | 9 | 10 | 11 | 9 | 1631300000 | 1361500000 | 1339300000 |
| Perilipin-3 | PLIN3 | 427 | 9 | 9 | 9 | 9 | 9 | 9 | 376840000 | 406400000 | 357120000 |
| Polyamine modulated factor 1 binding protein 1 | PMFBP1 | 869 | 3 | 3 | 3 | 3 | 3 | 3 | 728482500 | 0 | 780540000 |
| Thioredoxin domain-containing protein | PRDX1 | 199 | 2 | 2 | 2 | 2 | 2 | 2 | 9924200 | 11783000 | 7794300 |
| Peroxiredoxin 6 | Prdx6 | 23 | 2 | 2 | 2 | 2 | 2 | 2 | 3832150 | 3115701 | 3896400 |
| Peroxiredoxin-like 2A | PRXL2A | 229 | 3 | 0 | 2 | 2 | 0 | 1 | 4318609 | 0 | 4579200 |
| AAA domain-containing protein | PSMC3 | 442 | 5 | 5 | 4 | 5 | 5 | 4 | 0 | 121850 | 129929 |
| PCI domain-containing protein | PSMD6 | 389 | 2 | 2 | 2 | 2 | 1 | 1 | 2155656 | 1944626 | 2049300 |
| Proteasome activator complex subunit 1 | PSME1 | 249 | 1 | 1 | 2 | 1 | 1 | 2 | 0 | 2061968 | 1995200 |
| Ras-related protein Rab-10 | RAB10 | 188 | 2 | 2 | 2 | 2 | 2 | 2 | 82790000 | 70847000 | 65994000 |
| Ras-related protein Rab-11A | RAB11A | 216 | 2 | 2 | 2 | 2 | 2 | 2 | 11175000 | 10636000 | 11327000 |
| Ras-related protein Rab-18 | RAB18 | 206 | 7 | 7 | 7 | 7 | 7 | 7 | 482850000 | 614480000 | 572760000 |
| Ras-related protein Rab-1B | RAB1B | 147 | 11 | 11 | 12 | 2 | 2 | 2 | 1320301 | 0 | 1348800 |
| Ras-related protein Rab-2A | RAB2A | 212 | 3 | 3 | 3 | 3 | 3 | 3 | 31391000 | 39787000 | 28153000 |
| Ras-related protein Rab-5A | RAB5A | 215 | 2 | 2 | 2 | 2 | 2 | 2 | 24935000 | 36488000 | 30088000 |
| Ras-related protein Rab-7A | RAB7A | 207 | 2 | 2 | 2 | 2 | 2 | 2 | 6678100 | 8583400 | 7880400 |
| Ras-related C3 botulinum toxin substrate 1 | RAC1 | 180 | 2 | 1 | 2 | 2 | 1 | 2 | 25565000 | 0 | 28066155 |
| RAS like proto-oncogene A | RALA | 206 | 15 | 15 | 15 | 0 | 0 | 0 | 0 | 101919 | 110030 |
| RAS like proto-oncogene B | RALB | 206 | 2 | 2 | 2 | 2 | 2 | 2 | 2511831 | 0 | 2963600 |
| RAP2B protein | RAP2B | 183 | 3 | 3 | 3 | 3 | 3 | 3 | 356720 | 203644 | 332801 |
| Retinol binding protein 1 | RBP1 | 135 | 9 | 9 | 9 | 8 | 8 | 8 | 0 | 744802 | 603200 |
| Ras-like protein family member A | RHOA | 193 | 3 | 4 | 3 | 3 | 4 | 3 | 23828000 | 25741000 | 22951000 |
| Ribonuclease/angiogenin inhibitor 1 | RNH1 | 456 | 2 | 2 | 2 | 2 | 2 | 2 | 6368800 | 6359900 | 7074000 |
| Protein XRP2 | RP2 | 319 | 3 | 3 | 3 | 2 | 2 | 2 | 0 | 362802 | 350360 |
| Ribosomal_L16 domain-containing protein | RPL10L | 214 | 1 | 2 | 1 | 1 | 2 | 1 | 3077390 | 3533607 | 2530100 |
| 60S ribosomal protein L11 | RPL11 | 178 | 2 | 2 | 2 | 2 | 2 | 2 | 0 | 23720000 | 28194000 |
| 60S ribosomal protein L18 | RPL18 | 187 | 2 | 1 | 2 | 2 | 1 | 2 | 3923153 | 3957695 | 3399200 |
| 60S ribosomal protein L22 | RPL22 | 128 | 2 | 2 | 1 | 2 | 2 | 1 | 10459000 | 10420952 | 0 |
| TRASH domain-containing protein | RPL24 | 120 | 3 | 3 | 3 | 3 | 3 | 3 | 0 | 106294 | 96601 |
| 60S ribosomal protein L7a | RPL7A | 246 | 2 | 2 | 2 | 2 | 2 | 2 | 19425000 | 20551000 | 20937000 |
| 60S acidic ribosomal protein P0 | RPLP0 | 313 | 3 | 3 | 3 | 3 | 3 | 3 | 28071000 | 26870000 | 33432000 |
| 60S acidic ribosomal protein P1 | RPLP1 | 114 | 2 | 2 | 2 | 2 | 2 | 2 | 4914571 | 0 | 5337100 |
| 60S acidic ribosomal protein P2 | RPLP2 | 115 | 4 | 4 | 4 | 4 | 4 | 4 | 38378000 | 35379000 | 62206000 |
| Dolichyl-diphosphooligosaccharide--protein glycosyltransferase subunit 1 | RPN1 | 585 | 2 | 1 | 1 | 2 | 1 | 1 | 3365800 | 2286144 | 0 |
| Dolichyl-diphosphooligosaccharide--protein glycosyltransferase subunit 2 | RPN2 | 509 | 2 | 2 | 2 | 2 | 2 | 2 | 4462279 | 4479209 | 4126700 |
| Ribosomal_S17_N domain-containing protein | RPS11 | 158 | 2 | 2 | 2 | 2 | 2 | 2 | 0 | 6829099 | 6465300 |
| 40S ribosomal protein S12 | RPS12 | 126 | 7 | 8 | 8 | 7 | 8 | 8 | 9346698 | 9131561 | 9136200 |
| Ribosomal_S13_N domain-containing protein | RPS13 | 151 | 2 | 2 | 2 | 2 | 2 | 2 | 10717000 | 13726000 | 8762400 |
| Ribosomal protein S17 | RPS17 | 135 | 6 | 6 | 6 | 3 | 3 | 3 | 0 | 5480895 | 5466900 |
| 40S ribosomal protein S24 | RPS24 | 131 | 3 | 3 | 3 | 3 | 3 | 3 | 7806249 | 0 | 7624600 |
| KH type-2 domain-containing protein | RPS3 | 243 | 7 | 7 | 7 | 7 | 7 | 7 | 56414000 | 50403000 | 68163000 |
| Ribosomal_S7 domain-containing protein | RPS5 | 204 | 11 | 8 | 10 | 2 | 2 | 2 | 19394839 | 0 | 18741000 |
| 40S ribosomal protein S7 | RPS7 | 174 | 2 | 2 | 2 | 2 | 2 | 2 | 28378000 | 23271000 | 21792000 |
| 40S ribosomal protein S8 | RPS8 | 192 | 25 | 25 | 25 | 19 | 19 | 19 | 10945784 | 11287821 | 10896000 |
| S4 RNA-binding domain-containing protein | RPS9 | 194 | 3 | 3 | 3 | 3 | 3 | 3 | 14575000 | 15953000 | 13842000 |
| Rib_recp_KP_reg domain-containing protein | RRBP1 | 1518 | 4 | 4 | 4 | 4 | 4 | 4 | 7616600 | 9254000 | 7990900 |
| Reticulon | RTN4 | 198 | 19 | 19 | 19 | 14 | 14 | 14 | 4359892 | 4337919 | 4330900 |
| Protein S100-A12 | S100A12 | 92 | 8 | 6 | 8 | 4 | 4 | 4 | 175965 | 0 | 180482 |
| Protein S100-A8 | S100A8 | 89 | 2 | 2 | 2 | 2 | 2 | 2 | 14910000 | 15937000 | 13454000 |
| Protein S100-A9 | S100A9 | 151 | 2 | 2 | 2 | 2 | 2 | 2 | 11009000 | 11129000 | 11862000 |
| Serum amyloid A protein | SAA1 | 112 | 5 | 5 | 5 | 4 | 4 | 4 | 141980000 | 135450000 | 96685000 |
| GTP-binding protein SAR1b | SAR1B | 198 | 18 | 16 | 17 | 13 | 12 | 12 | 0 | 6455442 | 6604200 |
| Sacchrp_dh_NADP domain-containing protein | SCCPDH | 429 | 2 | 2 | 2 | 2 | 2 | 2 | 25889000 | 19596000 | 22952000 |
| SEC22 homolog B, vesicle trafficking protein | SEC22B | 215 | 3 | 3 | 3 | 3 | 3 | 3 | 117591 | 0 | 117530 |
| Methanethiol oxidase | SELENBP1 | 472 | 6 | 6 | 7 | 6 | 6 | 7 | 65918000 | 72633000 | 68061000 |
| Selenoprotein F | SELENOF | 124 | 4 | 4 | 4 | 4 | 4 | 4 | 4423500 | 4071135 | 4721316 |
| Alpha-1-antitrypsin transcript variant 2 | SERPINA1 | 230 | 2 | 2 | 1 | 1 | 1 | 0 | 2940048 | 0 | 2839100 |
| Nucleotide exchange factor SIL1 | SIL1 | 459 | 2 | 2 | 2 | 2 | 2 | 2 | 0 | 17864300 | 1757200 |
| Excitatory amino acid transporter 3 | SLC1A1 | 517 | 2 | 2 | 2 | 2 | 2 | 2 | 12187000 | 11084000 | 9991100 |
| Sodium/nucleoside cotransporter | SLC28A3 | 697 | 5 | 5 | 4 | 5 | 5 | 4 | 308100000 | 302960000 | 380750000 |
| Sodium-dependent phosphate transport protein 2B | SLC34A2 | 693 | 6 | 6 | 6 | 6 | 6 | 6 | 262900000 | 260700000 | 241620000 |
| Synaptosomal-associated protein | SNAP23 | 211 | 4 | 4 | 4 | 4 | 4 | 4 | 25966000 | 33755000 | 25065000 |
| PX domain-containing protein | SNX30 | 437 | 17 | 18 | 17 | 4 | 4 | 3 | 0 | 3526006 | 3961800 |
| Signal sequence receptor subunit 1 | SSR1 | 280 | 2 | 2 | 2 | 2 | 2 | 2 | 2803873 | 3154300 | 0 |
| STEAP4 metalloreductase | STEAP4 | 470 | 2 | 2 | 2 | 2 | 2 | 2 | 5842075 | 5371514 | 5394100 |
| PHB domain-containing protein | STOM | 284 | 8 | 8 | 8 | 8 | 8 | 8 | 107520000 | 96043000 | 84721000 |
| Stereocilin | STRC | 1730 | 5 | 5 | 5 | 2 | 2 | 1 | 0 | 14406 | 13465 |
| Syntaxin binding protein 2 | STXBP2 | 577 | 2 | 2 | 2 | 2 | 2 | 2 | 15462000 | 9687400 | 10159000 |
| Taste receptor type 2 | TAS2R | 309 | 17 | 18 | 17 | 4 | 4 | 3 | 873608 | 992658 | 897860 |
| T-complex protein 1 subunit alpha | TCP1 | 556 | 4 | 3 | 3 | 4 | 3 | 3 | 7299600 | 8322200 | 7304100 |
| Transcription factor AP-2 delta | TFAP2D | 452 | 2 | 2 | 2 | 2 | 2 | 2 | 0 | 719485 | 719510 |
| Thrombospondin-1 | THBS1 | 1126 | 2 | 2 | 2 | 2 | 2 | 2 | 7615600 | 7050400 | 6235500 |
| SMB domain-containing protein | TINAGL1 | 465 | 2 | 1 | 1 | 2 | 0 | 0 | 3848045 | 4025100 | 0 |
| TRANSKETOLASE_1 domain-containing protein | TKT | 623 | 4 | 4 | 4 | 4 | 4 | 4 | 20089000 | 19666000 | 24713000 |
| Toll like receptor 2 | TLR2 | 784 | 3 | 3 | 3 | 1 | 1 | 1 | 25481000 | 27699000 | 27563000 |
| Transmembrane protein 263 | TMEM263 | 115 | 2 | 2 | 1 | 2 | 2 | 1 | 4828064 | 4357142 | 4681500 |
| Tropomyosin alpha-3 chain | TPM3 | 248 | 7 | 5 | 5 | 7 | 5 | 5 | 0 | 328441 | 20507 |
| Translationally-controlled tumor protein | TPT1 | 156 | 4 | 4 | 4 | 4 | 4 | 4 | 7692553 | 7574423 | 7521000 |
| Tetraspanin-1 | TSPAN1 | 236 | 3 | 1 | 2 | 2 | 2 | 3 | 5547876 | 5057873 | 5725000 |
| Tubulin beta chain | TUBB | 167 | 2 | 2 | 2 | 2 | 2 | 2 | 15680000 | 18489000 | 15630000 |
| Thioredoxin domain containing 5 | TXNDC5 | 344 | 2 | 2 | 2 | 2 | 2 | 2 | 2121505 | 2344400 | 2262350 |
| Ubiquitin domain containing 1 | UBTD1 | 204 | 6 | 6 | 6 | 6 | 6 | 6 | 0 | 100657 | 101166 |
| UTP--glucose-1-phosphate uridylyltransferase | UGP2 | 504 | 2 | 2 | 2 | 2 | 2 | 2 | 7273600 | 6517500 | 4957200 |
| MHC_I-like_Ag-recog domain-containing protein | ULBP17 | 229 | 3 | 2 | 2 | 2 | 2 | 2 | 140871 | 0 | 132050 |
| Cytochrome b-c1 complex subunit 1, mitochondrial | UQCRC1 | 476 | 5 | 5 | 5 | 5 | 5 | 5 | 0 | 108840 | 111400 |
| Ubiquitin carboxyl-terminal hydrolase | USP10 | 782 | 1 | 2 | 1 | 1 | 2 | 1 | 12385430 | 11040817 | 12113000 |
| Vesicle amine transport 1 | VAT1 | 402 | 2 | 3 | 3 | 2 | 3 | 3 | 14017000 | 13070034 | 13396425 |
| Transitional endoplasmic reticulum ATPase | VCP | 806 | 4 | 4 | 5 | 4 | 4 | 5 | 22060000 | 18643000 | 22564000 |
| Vimentin | VIM | 388 | 2 | 2 | 2 | 2 | 2 | 2 | 12256000 | 10988000 | 12410000 |
| UBA domain-containing protein | VPS13D | 4364 | 4 | 1 | 1 | 2 | 1 | 1 | 101966 | 106218 | 0 |
| Vps53_N domain-containing protein | VPS53 | 832 | 7 | 7 | 8 | 4 | 4 | 4 | 138840 | 132806 | 131731 |
| WRN RecQ like helicase | WRN | 1382 | 1 | 2 | 1 | 1 | 2 | 1 | 13745962 | 1549917 | 15615000 |
| Xanthine dehydrogenase/oxidase | XDH | 1332 | 15 | 14 | 15 | 15 | 14 | 15 | 8597600000 | 6683500000 | 7669700000 |
| Synaptobrevin homolog YKT6 | YKT6 | 198 | 2 | 2 | 2 | 2 | 2 | 2 | 15626000 | 13889000 | 16455000 |
| 14-3-3 protein beta/alpha | YWHAB | 246 | 3 | 2 | 3 | 2 | 2 | 2 | 23098000 | 19992000 | 20130000 |
| 14-3-3 protein epsilon | YWHAE | 235 | 5 | 4 | 5 | 4 | 4 | 4 | 56801000 | 58466000 | 44319000 |
| 14-3-3 protein gamma | YWHAG | 225 | 2 | 1 | 2 | 2 | 1 | 2 | 0 | 7365660 | 7012200 |
| 14-3-3 protein theta | YWHAQ | 245 | 3 | 2 | 3 | 2 | 2 | 2 | 21306000 | 23599000 | 24073000 |
| Zinc finger and BTB domain containing 10 | ZBTB10 | 819 | 2 | 2 | 1 | 1 | 1 | 2 | 120042 | 108027 | 100864 |
| GM |  |  |  |  |  |  |  |  |  |  |  |
| ABC transporter domain-containing protein | ABCG2 | 643 | 6 | 6 | 6 | 6 | 6 | 6 | 323280000 | 273860000 | 275010000 |
| Acetyl-CoA carboxylase 1 | ACACA | 2329 | 6 | 6 | 6 | 6 | 6 | 6 | 30141000 | 27916000 | 28723000 |
| Cytoplasmic aconitate hydratase, Aconitase | ACO1 | 889 | 3 | 3 | 3 | 3 | 3 | 3 | 5185200 | 0 | 5162900 |
| AMP-binding domain-containing protein | ACSL1 | 672 | 11 | 10 | 11 | 11 | 10 | 10 | 86284000 | 108820000 | 88789000 |
| Long-chain-fatty-acid--CoA ligase 3 | ACSL3 | 695 | 4 | 3 | 4 | 4 | 3 | 3 | 27866000 | 41676000 | 31766000 |
| Acyl-CoA synthetase short-chain family member 3 | ACSS3 | 678 | 2 | 2 | 2 | 2 | 2 | 2 | 12937000 | 0 | 12882000 |
| Actin, alpha skeletal muscle | ACTA1 | 69 | 6 | 6 | 6 | 6 | 6 | 6 | 8679920 | 0 | 882860 |
| Actin, aortic smooth muscle | ACTA2 | 377 | 7 | 7 | 1 | 1 | 1 | 1 | 95780000 | 74922000 | 95853000 |
| Actin, cytoplasmic 1 | ACTB | 375 | 8 | 8 | 8 | 2 | 2 | 8 | 204940000 | 193000000 | 206950000 |
| Actin, alpha cardiac muscle 1 | ACTC1 | 374 | 8 | 8 | 8 | 2 | 2 | 2 | 63493000 | 79414000 | 76448000 |
| Actin, cytoplasmic 2 | ACTG1 | 344 | 7 | 7 | 7 | 1 | 1 | 1 | 2787708 | 0 | 2499900 |
| 1-acyl-sn-glycerol-3-phosphate acyltransferase | AGPAT1 | 287 | 2 | 2 | 2 | 2 | 2 | 2 | 1154204346 | 0 | 117480000 |
| Serum albumin | ALB | 607 | 7 | 7 | 7 | 7 | 7 | 7 | 140300000 | 142300000 | 131970000 |
| Aldedh domain-containing protein | ALDH1A1 | 487 | 2 | 2 | 2 | 2 | 2 | 2 | 10456000 | 12307000 | 10817000 |
| Aldehyde dehydrogenase family 3 member B1 | ALDH3B1 | 468 | 4 | 3 | 5 | 5 | 4 | 4 | 15298000 | 9774200 | 15226000 |
| Aldehyde dehydrogenase family 3 member B2 | ALDH3B2 | 466 | 2 | 1 | 2 | 2 | 1 | 1 | 2313700 | 0 | 2303700 |
| Fructose-bisphosphate aldolase | ALDOC | 364 | 1 | 2 | 1 | 1 | 2 | 1 | 458674965 | 0 | 42172000 |
| Annexin A1 | ANXA1 | 346 | 2 | 2 | 1 | 2 | 1 | 1 | 5724600 | 0 | 5699900 |
| Annexin A2 | ANXA2 | 339 | 2 | 2 | 2 | 2 | 2 | 2 | 14668000 | 10293000 | 14649000 |
| Annexin A3 | ANXA3 | 319 | 1 | 4 | 4 | 1 | 4 | 4 | 0 | 1728100 | 2287700 |
| Annexin A5 | ANXA5 | 321 | 2 | 2 | 2 | 2 | 2 | 2 | 11532000 | 13070000 | 11449000 |
| AP complex subunit sigma | AP1S2 | 157 | 9 | 7 | 9 | 9 | 8 | 8 | 14759973 | 1223703 | 1408400 |
| Apolipoprotein A-I | APOA1 | 265 | 2 | 2 | 2 | 2 | 2 | 2 | 18591000 | 21639000 | 18448000 |
| Apolipoprotein E | APOE | 316 | 3 | 3 | 3 | 2 | 2 | 2 | 4994100 | 0 | 4972600 |
| Rho-GAP domain-containing protein | ARHGAP31 | 1440 | 8 | 8 | 8 | 8 | 8 | 7 | 82577457 | 86799720 | 8860300 |
| Rho GDP-dissociation inhibitor 1 | ARHGDIA | 204 | 4 | 4 | 4 | 4 | 4 | 4 | 18801000 | 0 | 18720000 |
| Actin-related protein 2/3 complex subunit 5 | ARPC5 | 151 | 2 | 2 | 2 | 2 | 2 | 2 | 0 | 4586965 | 462080 |
| Adipose triglyceride lipase | ATGL | 486 | 3 | 3 | 3 | 3 | 3 | 3 | 11553000 | 16081000 | 10505000 |
| Cation-transporting ATPase | ATP13A4 | 1180 | 3 | 3 | 3 | 3 | 3 | 3 | 5051500 | 0 | 5029700 |
| ATP synthase subunit alpha, mitochondrial | ATP5F1A | 553 | 2 | 1 | 2 | 2 | 1 | 2 | 3606500 | 0 | 3591000 |
| V-type proton ATPase subunit B | ATP6V1B1 | 402 | 5 | 4 | 5 | 5 | 4 | 4 | 50449000 | 60166000 | 49823000 |
| CUE domain-containing protein | AUP1 | 410 | 2 | 1 | 1 | 2 | 1 | 1 | 912750000 | 9441006616 | 908810000 |
| Bac7.5 protein | bac7.5 | 190 | 4 | 4 | 4 | 3 | 3 | 3 | 68132000 | 80827000 | 82830000 |
| Butyrophilin subfamily 1 member A1 | BTN1A1 | 526 | 12 | 12 | 12 | 3 | 3 | 3 | 1990100000 | 1837800000 | 1820500000 |
| Complement component C3 | C3 | 349 | 3 | 4 | 3 | 0 | 0 | 4 | 858790000 | 693110000 | 865430000 |
| C4b-binding protein alpha chain | C4BPA | 599 | 2 | 1 | 2 | 2 | 1 | 2 | 7657657 | 7691547 | 7897978 |
| Cathelicidin-1 | CATHL1A | 155 | 3 | 3 | 3 | 2 | 2 | 2 | 12594000 | 13789000 | 15571000 |
| Cathelicidin-2 | CATHL2 | 176 | 2 | 1 | 1 | 2 | 1 | 1 | 5429436 | 0 | 5234300 |
| SAP domain-containing protein | CCAR1 | 1138 | 9 | 10 | 10 | 9 | 10 | 10 | 0 | 314184663 | 31177000 |
| Coiled-coil domain containing 18 | CCDC18 | 1453 | 2 | 2 | 2 | 2 | 2 | 2 | 7599300 | 9120900 | 7723400 |
| DUF4200 domain-containing protein | CCDC42 | 316 | 2 | 2 | 2 | 1 | 2 | 1 | 0 | 3423502 | 3693600 |
| CD36 antigen | CD36 | 472 | 2 | 1 | 1 | 2 | 1 | 1 | 13256000 | 131786094 | 124909859 |
| CD81 antigen | CD81 | 236 | 2 | 2 | 2 | 2 | 2 | 2 | 31023000 | 0 | 30889000 |
| CD9 antigen | CD9 | 219 | 3 | 3 | 3 | 3 | 3 | 3 | 10409000 | 9190400 | 10594000 |
| Cathelicidin-3.4 | ChBac3.4 | 26 | 2 | 2 | 2 | 2 | 1 | 1 | 21190000 | 17968000 | 17128000 |
| Chromodomain helicase DNA binding protein 3 | CHD3 | 1972 | 3 | 3 | 3 | 3 | 3 | 3 | 0 | 9573056 | 901590 |
| Calcineurin B homologous protein 1 | CHP1 | 195 | 5 | 5 | 6 | 5 | 5 | 6 | 0 | 13524185 | 1650100 |
| Calcium and integrin-binding protein 1 | CIB1 | 191 | 4 | 3 | 4 | 3 | 3 | 3 | 9005000 | 0 | 8966200 |
| Cell death-inducing DFFA-like effector a | CIDEA | 202 | 2 | 2 | 2 | 2 | 2 | 2 | 13611000 | 10105000 | 10085000 |
| Creatine kinase M-type | CKM | 381 | 8 | 8 | 7 | 8 | 8 | 7 | 0 | 6974948 | 686160 |
| Zeta-crystallin | CRYZ | 330 | 3 | 3 | 3 | 3 | 3 | 3 | 2660400 | 2674500 | 0 |
| Alpha-S1-casein | CSN1S1 | 214 | 6 | 6 | 6 | 5 | 5 | 5 | 146690000 | 155380000 | 165700000 |
| Alpha-s2-casein | CSN1S2 | 223 | 9 | 9 | 9 | 3 | 2 | 9 | 2324500000 | 2244600000 | 2916100000 |
| Beta-casein | CSN2 | 222 | 5 | 4 | 5 | 4 | 3 | 4 | 2907700000 | 4262900000 | 2681600000 |
| Kappa casein | CSN3 | 141 | 2 | 2 | 2 | 2 | 2 | 2 | 28892000 | 31987000 | 28412000 |
| Cleavage stimulation factor subunit 2 | CSTF2 | 190 | 5 | 6 | 6 | 5 | 6 | 6 | 29819000 | 0 | 29690000 |
| CCCTC-binding factor | CTCF | 730 | 15 | 15 | 15 | 15 | 15 | 15 | 1025988 | 1057352 | 1023255 |
| Homeobox protein cut-like | CUX1 | 1534 | 2 | 1 | 3 | 0 | 0 | 2 | 7912100 | 0 | 7878000 |
| Cytochrome b-245 light chain | CYBA | 67 | 3 | 2 | 4 | 1 | 1 | 2 | 38407298 | 3340700 | 34235002 |
| Dehydrogenase/reductase 1 | DHRS1 | 313 | 2 | 2 | 2 | 2 | 2 | 2 | 18691000 | 24984000 | 17981000 |
| Dynein heavy chain 7, axonemal | DNAH7 | 4016 | 8 | 7 | 8 | 8 | 7 | 8 | 0 | 40004432 | 44929000 |
| Chaperone protein DnaJ | dnaJ | 376 | 4 | 3 | 4 | 2 | 1 | 2 | 0 | 1039140 | 1022900 |
| Dipeptidyl peptidase 4 | DPP4 | 762 | 3 | 3 | 3 | 3 | 3 | 3 | 21115000 | 18332000 | 22219000 |
| Elongation factor 1-alpha 1 | EEF1A1 | 462 | 14 | 14 | 14 | 4 | 2 | 2 | 3201900 | 0 | 3188100 |
| Tr-type G domain-containing protein | EEF2 | 858 | 2 | 2 | 2 | 2 | 2 | 2 | 9122900 | 10073000 | 9117300 |
| EH domain-containing protein 1 | EHD1 | 489 | 1 | 2 | 1 | 1 | 2 | 1 | 219926603 | 21638000 | 217000561 |
| EH domain containing 4 | EHD4 | 540 | 3 | 3 | 3 | 3 | 3 | 3 | 448250000 | 468770000 | 411800000 |
| Eukaryotic initiation factor 4A-II | EIF4A2 | 363 | 11 | 12 | 12 | 11 | 12 | 12 | 3175500 | 35110010 | 3161800 |
| Neutrophil elastase | ELANE | 267 | 11 | 10 | 12 | 9 | 8 | 10 | 12979116 | 13831376 | 1745600 |
| GTPase Der | engA | 436 | 2 | 2 | 2 | 2 | 2 | 2 | 3377535 | 3511010 | 3091103 |
| FERM domain-containing protein | EZR | 551 | 3 | 3 | 3 | 3 | 3 | 3 | 6812500 | 0 | 6783100 |
| FABP domain-containing protein | FABP3 | 133 | 6 | 7 | 6 | 6 | 7 | 6 | 65789461 | 6422000 | 68476011 |
| UBX domain-containing protein | FAF2 | 445 | 5 | 4 | 5 | 5 | 4 | 5 | 2760291 | 2463620 | 243820 |
| Family with sequence similarity 186 member B | FAM186B | 905 | 1 | 2 | 2 | 1 | 2 | 2 | 103830000 | 0 | 103380000 |
| FA complementation group I | FANCI | 1086 | 5 | 5 | 5 | 5 | 5 | 5 | 599464800 | 576029000 | 56311000 |
| Fatty acid synthase | FASN | 2510 | 26 | 26 | 26 | 26 | 26 | 26 | 673930000 | 628740000 | 660950000 |
| Fibrinogen C-terminal domain-containing protein | FGG | 427 | 2 | 1 | 1 | 2 | 1 | 1 | 0 | 5994648 | 5760291 |
| Peptidylprolyl isomerase | FKBP11 | 191 | 6 | 6 | 6 | 6 | 6 | 6 | 0 | 21318726 | 2113000 |
| Folate_rec domain-containing protein | FOLR3 | 258 | 2 | 2 | 2 | 2 | 1 | 1 | 12477000 | 0 | 12423000 |
| Acidic amino acid decarboxylase | GADL1 | 449 | 26 | 26 | 26 | 26 | 26 | 26 | 0 | 2327359 | 2250600 |
| Gal_mutarotas_2 domain-containing protein | GANAB | 869 | 3 | 3 | 3 | 3 | 3 | 3 | 21372000 | 23420000 | 20425000 |
| Rab GDP dissociation inhibitor | GDI2 | 430 | 2 | 2 | 2 | 2 | 2 | 2 | 10114000 | 0 | 10070000 |
| Guanine nucleotide binding protein alpha inhibiting 2 | Gi2 | 306 | 2 | 2 | 2 | 2 | 2 | 2 | 21852000 | 23920000 | 20755000 |
| SCP domain-containing protein | GLIPR1L1 | 284 | 19 | 18 | 19 | 19 | 18 | 19 | 4153028 | 0 | 43208000 |
| Glomulin | GLMN | 574 | 3 | 3 | 3 | 3 | 3 | 3 | 144741 | 103558 | 106769 |
| GLTP domain-containing protein | GLTP | 209 | 2 | 2 | 2 | 2 | 2 | 2 | 6760200 | 6986800 | 6802800 |
| Glycosylation-dependent cell adhesion molecule 1 | GLYCAM1 | 152 | 3 | 3 | 3 | 3 | 3 | 3 | 28366000 | 33188000 | 40680000 |
| Guanine nucleotide-binding protein subunit alpha-11 | GNA11 | 314 | 3 | 4 | 4 | 2 | 2 | 2 | 0 | 1723200 | 17683438 |
| Guanine nucleotide binding proteim alpha inhibiting activity polypeptide 3 | GNAI3 | 70 | 4 | 3 | 3 | 4 | 3 | 3 | 4206178 | 0 | 4174600 |
| Guanine nucleotide binding protein alpha activating activity polypeptide O | GNAO1 | 134 | 2 | 2 | 2 | 2 | 2 | 2 | 7395700 | 7530168 | 7363800 |
| G protein subunit alpha q | GNAQ | 301 | 2 | 2 | 1 | 2 | 2 | 1 | 2865400 | 0 | 2853100 |
| Guanine nucleotide-binding protein G(I)/G(S)/G(T) subunit beta-1 | GNB1 | 279 | 2 | 2 | 1 | 1 | 2 | 1 | 5437800 | 5433214 | 5414400 |
| Guanine nucleotide-binding protein G(I)/G(S)/G(T) subunit beta-2 | GNB2 | 340 | 2 | 2 | 2 | 1 | 2 | 2 | 40480000 | 28334000 | 39269000 |
| Guanine nucleotide-binding protein subunit gamma | GNG12 | 72 | 2 | 0 | 1 | 2 | 0 | 1 | 6860500 | 0 | 6830900 |
| G_PROTEIN_RECEP_F1_2 domain-containing protein | OR10G2 | 318 | 2 | 2 | 2 | 2 | 2 | 2 | 7417941 | 7972246 | 7788400 |
| Gelsolin isoform b | GSN | 731 | 2 | 2 | 2 | 2 | 2 | 2 | 14191000 | 11689000 | 14270000 |
| Hemoglobin subunit beta-C | HBBC | 141 | 3 | 3 | 3 | 3 | 3 | 2 | 0 | 918606 | 963000 |
| Haloacid dehalogenase like hydrolase domain containing 5 | HDHD5 | 390 | 2 | 2 | 2 | 1 | 2 | 1 | 1497246 | 0 | 6712100 |
| Heme-binding protein 1 | HEBP1 | 191 | 3 | 3 | 3 | 3 | 3 | 3 | 50180000 | 44298000 | 48633000 |
| Histone cluster 1 H1 family member b | HIST1H1B | 226 | 2 | 2 | 2 | 2 | 2 | 2 | 13873000 | 14712000 | 16679000 |
| Histone cluster 1 H1 family member e | HIST1H1E | 219 | 2 | 2 | 2 | 2 | 2 | 2 | 55405000 | 52448000 | 49001000 |
| Heat shock protein HSP 90-beta | HSP90AB1 | 471 | 4 | 4 | 4 | 3 | 3 | 4 | 19446000 | 19511000 | 14967000 |
| Endoplasmin | HSP90B1 | 804 | 2 | 1 | 2 | 2 | 1 | 2 | 0 | 1211208 | 1292600 |
| Heat shock 70kDa protein 5 | HSPA5 | 655 | 4 | 4 | 3 | 3 | 3 | 3 | 15071000 | 18132000 | 13770000 |
| Heat shock cognate 71 kDa protein | HSPA8 | 590 | 4 | 3 | 4 | 3 | 2 | 3 | 30010000 | 36035000 | 29638000 |
| Heat shock protein alpha | HSPCA | 733 | 2 | 2 | 2 | 2 | 2 | 2 | 1517900 | 1857500 | 1738700 |
| INT_SG_DDX_CT_C domain-containing protein | INTS6 | 828 | 4 | 4 | 4 | 3 | 3 | 4 | 90320000 | 106950000 | 91742000 |
| Inos-1-P_synth domain-containing protein | ISYNA1 | 547 | 2 | 2 | 2 | 2 | 2 | 2 | 0 | 13243226 | 13372000 |
| VWFA domain-containing protein | ITGAM | 1152 | 2 | 2 | 2 | 2 | 2 | 2 | 28183000 | 25311000 | 23650000 |
| Integrin beta | ITGB2 | 750 | 12 | 10 | 11 | 1 | 1 | 2 | 3918300 | 3916407 | 3901400 |
| Immunoglobulin J chain | JCHAIN | 158 | 16 | 15 | 16 | 16 | 15 | 16 | 13454000 | 13604131 | 13396000 |
| KH-type splicing regulatory protein | KHSRP | 708 | 2 | 1 | 2 | 2 | 1 | 1 | 4475200 | 0 | 4455900 |
| Fmp27_GFWDK domain-containing protein | KIAA0100 | 2213 | 7 | 5 | 7 | 7 | 5 | 5 | 65787000 | 69190000 | 71510000 |
| Kinesin-like protein | KIF3A | 858 | 3 | 3 | 3 | 3 | 3 | 3 | 0 | 3384904 | 3837400 |
| Keratin, type II cytoskeletal 1 | KRT1 | 644 | 17 | 16 | 17 | 11 | 11 | 11 | 716590000 | 718580000 | 696390000 |
| Keratin, type I cytoskeletal 10 | KRT10 | 584 | 5 | 5 | 5 | 3 | 3 | 3 | 34741000 | 36366000 | 32017000 |
| Keratin, type I cytoskeletal 14 | KRT14 | 472 | 7 | 7 | 7 | 3 | 3 | 3 | 10383000 | 10814000 | 12717000 |
| Keratin, type I cytoskeletal 18 | Krt18 | 423 | 8 | 9 | 9 | 7 | 8 | 8 | 125460000 | 132240000 | 143090000 |
| Keratin, type I cytoskeletal 19 | KRT19 | 400 | 5 | 4 | 4 | 5 | 4 | 4 | 32697000 | 34224000 | 29148000 |
| Keratin, type II cytoskeletal 2 epidermal | KRT2 | 639 | 17 | 15 | 15 | 13 | 11 | 11 | 235550000 | 241690000 | 254010000 |
| Keratin, type II cytoskeletal 5 | KRT5 | 164 | 4 | 3 | 3 | 3 | 3 | 3 | 131280000 | 108050000 | 156360000 |
| Keratin, type II cytoskeletal 6C | KRT6C | 564 | 6 | 6 | 6 | 2 | 2 | 2 | 7138400 | 7744600 | 7680400 |
| Keratin, type II cytoskeletal 71 | KRT71 | 524 | 3 | 3 | 3 | 3 | 3 | 1 | 0 | 2040700 | 4844500 |
| Keratin, type II cytoskeletal 72 | KRT72 | 511 | 2 | 2 | 2 | 1 | 2 | 1 | 12584173 | 13356678 | 1356000 |
| Keratin, type II cytoskeletal 1b | Krt77 | 572 | 3 | 3 | 3 | 2 | 2 | 1 | 0 | 526077 | 578290 |
| Keratin, type II cytoskeletal 79 | KRT79 | 531 | 2 | 1 | 1 | 2 | 0 | 0 | 24162000 | 23521000 | 19878000 |
| Keratin, type I cytoskeletal 9 | KRT9 | 623 | 20 | 21 | 21 | 19 | 20 | 20 | 501010000 | 470690000 | 517120000 |
| Alpha-lactalbumin | LALBA | 142 | 3 | 4 | 4 | 1 | 1 | 2 | 0 | 6134500 | 2095600 |
| Laminin subunit beta 1 | LAMB1 | 1786 | 8 | 9 | 9 | 8 | 9 | 9 | 10439365 | 10328059 | 10581000 |
| Lipopolysaccharide-binding protein | LBP | 481 | 2 | 2 | 2 | 2 | 2 | 2 | 2064700 | 0 | 2055800 |
| Lipocln_cytosolic_FA-bd_dom domain-containing protein | LCN2 | 200 | 2 | 2 | 2 | 2 | 2 | 2 | 8655200 | 7835300 | 8210700 |
| Beta-lactoglobulin | LGB | 180 | 6 | 6 | 6 | 3 | 3 | 6 | 4787100000 | 5586200000 | 4795100000 |
| LIM domain kinase 2 | LIMK2 | 612 | 2 | 2 | 2 | 2 | 2 | 2 | 4105310 | 4678021 | 4857900 |
| Histone H2A | H2AFV | 126 | 2 | 2 | 2 | 2 | 2 | 2 | 1637800000 | 1463300000 | 1627000000 |
| IF rod domain-containing protein | LOC101111440 | 531 | 2 | 2 | 2 | 2 | 2 | 2 | 0 | 4748400 | 4732074 |
| Clusterin | CLU | 439 | 2 | 3 | 2 | 2 | 3 | 2 | 0 | 2124538 | 2131700 |
| C-type lectin domain-containing protein | LOC101123029 | 315 | 2 | 2 | 2 | 2 | 2 | 2 | 24598000 | 27402000 | 23686000 |
| BPI1 domain-containing protein | BPIFB1 | 161 | 3 | 4 | 3 | 3 | 4 | 3 | 5210400 | 5135730 | 5187900 |
| Thioredoxin domain-containing protein | PRDX1 | 278 | 4 | 5 | 6 | 4 | 5 | 6 | 0 | 6998182 | 6359000 |
| Olfactory receptor | OR6S1 | 331 | 4 | 4 | 4 | 4 | 4 | 4 | 14014802 | 16432000 | 16794969 |
| NADH-cytochrome b5 reductase | LOC102173645 | 295 | 9 | 10 | 10 | 9 | 10 | 10 | 0 | 1947631 | 1971700 |
| ATP synthase subunit alpha | LOC102183240 | 547 | 2 | 1 | 2 | 2 | 1 | 2 | 400312 | 401468 | 413210 |
| Lipoprotein lipase | LPL | 459 | 2 | 2 | 2 | 2 | 2 | 2 | 85514000 | 64358000 | 84858000 |
| LRAT domain-containing protein | LRATD2 | 288 | 3 | 3 | 2 | 3 | 3 | 2 | 1996559 | 1999269 | 1987000 |
| Leucine rich repeat containing 24 | LRRC24 | 440 | 4 | 5 | 5 | 4 | 5 | 5 | 62064000 | 57852000 | 54166000 |
| LRRC57 protein | LRRC57 | 239 | 2 | 2 | 2 | 2 | 2 | 2 | 5186100 | 6806700 | 0 |
| Lanosterol synthase | LSS | 732 | 2 | 2 | 2 | 2 | 2 | 2 | 6055400 | 0 | 6029300 |
| Lactotransferrin | LTF | 708 | 2 | 2 | 2 | 2 | 2 | 2 | 23905000 | 27237000 | 23178000 |
| MAP28 protein | map28 | 158 | 3 | 3 | 3 | 2 | 2 | 2 | 7372500 | 7461200 | 6523200 |
| MAP34-A protein | map34-A | 165 | 3 | 3 | 2 | 3 | 3 | 2 | 40514428 | 43554450 | 42687000 |
| Myoglobin | MB | 154 | 7 | 7 | 6 | 7 | 7 | 6 | 1288211 | 1355445 | 1222300 |
| Mediator of RNA polymerase II transcription subunit 4 | MED4 | 197 | 7 | 7 | 6 | 7 | 7 | 6 | 2303426 | 2290597 | 2010500 |
| Melanotransferrin | MELTF | 739 | 3 | 3 | 3 | 3 | 3 | 3 | 157700000 | 149240000 | 145030000 |
| Methyltransferase like 9 | METTL9 | 256 | 10 | 9 | 9 | 8 | 7 | 8 | 124560000 | 151640000 | 129210000 |
| Lactadherin | MFGE8 | 398 | 5 | 5 | 5 | 5 | 5 | 5 | 111100000 | 82685000 | 85262000 |
| MutL homolog 3 | MLH3 | 1485 | 5 | 6 | 6 | 5 | 6 | 6 | 16552 | 13116 | 17964 |
| Myeloperoxidase | MPO | 719 | 2 | 2 | 2 | 2 | 2 | 2 | 9492900 | 0 | 9451900 |
| SEA domain-containing protein | MUC1 | 578 | 2 | 2 | 2 | 2 | 2 | 2 | 155740000 | 132700000 | 188210000 |
| Mucin-15 | MUC15 | 330 | 4 | 4 | 4 | 4 | 4 | 4 | 31262000 | 0 | 31127000 |
| UPF0122 protein MYEA_6500 | MYEA_6500 | 107 | 5 | 5 | 4 | 2 | 2 | 0 | 47655000 | 0 | 47450000 |
| Myosin-2 | MYH2 | 1928 | 12 | 12 | 14 | 10 | 9 | 11 | 48664000 | 44070000 | 42822000 |
| Myosin-7 | MYH7 | 1937 | 3 | 3 | 3 | 3 | 3 | 3 | 48561000 | 47492000 | 53096000 |
| Myosin-9 | MYH9 | 1965 | 2 | 2 | 2 | 2 | 2 | 2 | 3105100 | 3119800 | 2488700 |
| Myosin light chain 1 transcript variant 2 | MYL1b | 192 | 3 | 3 | 2 | 3 | 3 | 2 | 3633500 | 3406300 | 3367600 |
| NADH-ubiquinone oxidoreductase 75 kDa subunit | NDUFS1 | 701 | 8 | 8 | 8 | 8 | 8 | 8 | 0 | 7741222 | 71832000 |
| NLR family pyrin domain containing 1 | NLRP1 | 1089 | 3 | 3 | 3 | 3 | 3 | 3 | 18850000 | 14845000 | 18863000 |
| Putative heparan sulfate proteoglycan | novocan | 834 | 4 | 3 | 4 | 4 | 3 | 4 | 3754144 | 3966154 | 3693600 |
| TPR_REGION domain-containing protein | NOXA1 | 238 | 2 | 2 | 1 | 2 | 2 | 1 | 34651965 | 3382156 | 34563000 |
| 3Beta_HSD domain-containing protein | NSDHL | 347 | 2 | 1 | 2 | 2 | 1 | 1 | 15362000 | 27426000 | 0 |
| Protein disulfide-isomerase | P4HB | 507 | 1 | 2 | 1 | 1 | 2 | 2 | 14124000 | 14093305 | 14063000 |
| Protocadherin 19 | PCDH19 | 1100 | 3 | 3 | 3 | 3 | 3 | 3 | 6718200 | 0 | 6000600 |
| PACT_coil_coil domain-containing protein | PCNT | 3289 | 4 | 3 | 4 | 4 | 3 | 4 | 0 | 581451 | 638590 |
| Protein disulfide-isomerase A3 | PDIA3 | 449 | 11 | 11 | 11 | 11 | 11 | 11 | 10026000 | 0 | 9982300 |
| Phosphatidylethanolamine binding protein 4 | PEBP4 | 223 | 15 | 14 | 12 | 4 | 4 | 0 | 65690969 | 60075898 | 57331000 |
| PAS domain-containing protein | PER1 | 1266 | 3 | 3 | 3 | 3 | 3 | 3 | 0 | 831430 | 796702 |
| 6-phosphogluconate dehydrogenase, decarboxylating | PGD | 473 | 2 | 3 | 2 | 2 | 3 | 2 | 2422000 | 7208000 | 0 |
| Phosphoglycerate kinase | PGK1 | 415 | 2 | 2 | 2 | 2 | 2 | 2 | 3074200 | 0 | 3061000 |
| Peptidoglycan-recognition protein | PGLYRP1 | 190 | 2 | 2 | 2 | 2 | 2 | 2 | 26097000 | 20159000 | 18348000 |
| PHD finger protein 3 | PHF3 | 2028 | 2 | 2 | 3 | 2 | 2 | 3 | 7929100 | 0 | 7894900 |
| Polymeric immunoglobulin receptor | PIGR | 747 | 7 | 7 | 5 | 7 | 7 | 5 | 106357 | 119091 | 1010949 |
| Perilipin-1 | PLIN1 | 450 | 13 | 14 | 13 | 13 | 14 | 14 | 3044800000 | 3632300000 | 3313600000 |
| Perilipin-2 | PLIN2 | 450 | 7 | 7 | 7 | 7 | 7 | 7 | 75956000 | 64197000 | 62037000 |
| Perilipin-3 | PLIN3 | 427 | 8 | 8 | 8 | 8 | 8 | 8 | 248170000 | 299310000 | 246780000 |
| Polyamine modulated factor 1 binding protein 1 | PMFBP1 | 869 | 8 | 9 | 9 | 4 | 4 | 4 | 0 | 61700517 | 61878000 |
| WRNPLPNID domain-containing protein | PPP4R1 | 960 | 3 | 3 | 3 | 3 | 3 | 3 | 20109000 | 0 | 20022000 |
| Prominin 2 | PROM2 | 687 | 3 | 2 | 4 | 2 | 1 | 3 | 12356578 | 12202021 | 12230000 |
| Myeloblastin | PRTN3 | 253 | 2 | 1 | 1 | 2 | 1 | 1 | 15671156 | 15736450 | 15961000 |
| Ras-related protein Rab-11A | RAB11A | 216 | 2 | 2 | 2 | 2 | 2 | 2 | 16371000 | 16805000 | 16707000 |
| Ras-related protein Rab-18 | RAB18 | 206 | 2 | 2 | 2 | 2 | 2 | 2 | 12066000 | 12349000 | 11612000 |
| Ras-related protein Rab-1B | RAB1B | 147 | 10 | 10 | 9 | 10 | 10 | 9 | 0 | 4476977 | 4492600 |
| Ras-related protein Rab-25 | RAB25 | 213 | 2 | 2 | 2 | 2 | 2 | 2 | 4857100 | 4692000 | 4008200 |
| Ras-related protein Rab-2A | RAB2A | 212 | 3 | 2 | 3 | 3 | 2 | 2 | 30503000 | 41411000 | 30770000 |
| Ras-related protein Rab-5C | RAB5C | 216 | 8 | 8 | 6 | 8 | 8 | 6 | 9140600 | 11642000 | 9065900 |
| Ras-related protein Rab-6B | RAB6B | 160 | 2 | 2 | 2 | 2 | 2 | 2 | 13002000 | 9346000 | 8437100 |
| Ras-related protein Rab-7A | RAB7A | 207 | 2 | 2 | 2 | 2 | 2 | 2 | 13517000 | 17434000 | 13496000 |
| Ras-related C3 botulinum toxin substrate 1 | RAC1 | 180 | 16 | 16 | 14 | 16 | 16 | 14 | 0 | 0 | 27832000 |
| Ras related v-ral simian leukemia viral oncogene homolog A | RALA | 206 | 4 | 4 | 4 | 2 | 2 | 2 | 9955400 | 9017100 | 9666000 |
| Ras-related protein Rap-1A | RAP1A | 184 | 4 | 4 | 4 | 4 | 4 | 4 | 55438000 | 44540000 | 55751000 |
| Ras association domain family member 1 | RASSF1 | 333 | 4 | 5 | 4 | 1 | 4 | 1 | 5023143 | 4880492 | 5037000 |
| Resistin | RETN | 109 | 12 | 15 | 14 | 12 | 14 | 13 | 3253680 | 3561271 | 3199400 |
| Ras-like protein family member A | RHOA | 193 | 3 | 3 | 3 | 3 | 3 | 3 | 61152000 | 61615000 | 53698000 |
| Ribosomal_L18e/L15P domain-containing protein | RPL18 | 187 | 4 | 4 | 4 | 3 | 3 | 3 | 0 | 2125919 | 2021500 |
| 60S acidic ribosomal protein P0 | RPLP0 | 313 | 8 | 7 | 7 | 8 | 7 | 7 | 4870500 | 0 | 4849500 |
| Dolichyl-diphosphooligosaccharide--protein glycosyltransferase subunit 2 | RPN2 | 509 | 9 | 9 | 9 | 9 | 9 | 9 | 17944465 | 17952059 | 1795600 |
| KH type-2 domain-containing protein | RPS3 | 243 | 2 | 2 | 2 | 2 | 2 | 2 | 8856500 | 6967700 | 8908800 |
| Ribosomal_S7 domain-containing protein | RPS5 | 204 | 3 | 3 | 3 | 3 | 3 | 3 | 3733200 | 3146900 | 3015800 |
| Reticulon | RTN4 | 198 | 3 | 3 | 3 | 3 | 3 | 3 | 5094900 | 0 | 5073000 |
| Protein S100 | S100A12 | 92 | 18 | 18 | 17 | 18 | 18 | 17 | 14596475 | 12420497 | 12082000 |
| Protein S100-A8 | S100A8 | 89 | 12 | 10 | 12 | 10 | 8 | 10 | 35040000 | 0 | 20104000 |
| Protein S100-A9 | S100A9 | 151 | 7 | 7 | 7 | 7 | 7 | 7 | 8624325 | 8758604 | 8365800 |
| Serum amyloid A protein | SAA1 | 112 | 4 | 4 | 2 | 2 | 2 | 2 | 65385000 | 72640000 | 66909000 |
| Serum amyloid A-3 protein | SAA3 | 128 | 12 | 11 | 12 | 12 | 11 | 12 | 585132 | 519093 | 573350 |
| GTP-binding protein SAR1a | SAR1A | 198 | 3 | 2 | 2 | 1 | 2 | 2 | 0 | 1496475 | 1369200 |
| GTP-binding protein SAR1b | SAR1B | 198 | 2 | 2 | 2 | 2 | 2 | 2 | 5174800 | 5498149 | 5152500 |
| Sacchrp_dh_NADP domain-containing protein | SCCPDH | 429 | 2 | 2 | 2 | 2 | 2 | 2 | 0 | 3493743 | 3233300 |
| Ig-like domain-containing protein | SCN2B | 215 | 3 | 3 | 3 | 3 | 3 | 3 | 1730632 | 1657490 | 17596475 |
| Septin-type G domain-containing protein | SEPT3 | 358 | 10 | 10 | 10 | 10 | 10 | 10 | 106475 | 104790 | 102050 |
| Serpin A3-1 | SERPINA3-1 | 411 | 7 | 6 | 8 | 1 | 6 | 6 | 2245600 | 2242959 | 2235900 |
| SERPIN domain-containing protein | SERPINC1 | 462 | 13 | 11 | 12 | 13 | 11 | 12 | 130632 | 139053 | 137490 |
| Excitatory amino acid transporter 3 | SLC1A1 | 517 | 2 | 2 | 2 | 2 | 2 | 2 | 50036000 | 51403000 | 48757000 |
| Sodium/nucleoside cotransporter | SLC28A3 | 675 | 20 | 21 | 19 | 20 | 21 | 19 | 2384800 | 23219053 | 2374500 |
| Solute carrier family 2, facilitated glucose transporter member 3 | SLC2A3 | 494 | 4 | 3 | 3 | 4 | 3 | 3 | 0 | 4136875 | 4173600 |
| Solute carrier family 31 member 1 | SLC31A1 | 189 | 7 | 7 | 8 | 2 | 3 | 3 | 10045000 | 10001051 | 10002000 |
| Sodium-dependent phosphate transport protein 2B | SLC34A2 | 693 | 5 | 5 | 5 | 5 | 5 | 5 | 280680000 | 316280000 | 306400000 |
| Synaptosomal-associated protein | SNAP23 | 211 | 2 | 3 | 2 | 2 | 3 | 3 | 34342000 | 32036000 | 32038000 |
| PX domain-containing protein | SNX30 | 437 | 3 | 3 | 3 | 3 | 3 | 3 | 0 | 3501865 | 3965200 |
| STEAP4 metalloreductase | STEAP4 | 470 | 6 | 6 | 6 | 6 | 6 | 6 | 11872000 | 0 | 11821000 |
| PHB domain-containing protein | STOM | 284 | 7 | 7 | 7 | 7 | 7 | 7 | 84746000 | 93720000 | 94041000 |
| Stereocilin | STRC | 1730 | 2 | 2 | 2 | 2 | 1 | 1 | 0 | 1913269 | 1960300 |
| Syntaxin binding protein 2 | STXBP2 | 577 | 3 | 3 | 3 | 0 | 2 | 2 | 12501865 | 123796542 | 12251000 |
| Taste receptor type 2 | TAS2R | 309 | 1 | 2 | 1 | 1 | 2 | 1 | 0 | 12802363 | 1254600 |
| Tektin-4 | TEKT4 | 447 | 2 | 2 | 2 | 2 | 2 | 2 | 7245200 | 8716000 | 7216900 |
| Transcription factor AP-2 delta | TFAP2D | 452 | 6 | 6 | 6 | 2 | 2 | 2 | 13444803 | 0 | 13636000 |
| Transketolase | TKT | 472 | 3 | 3 | 3 | 3 | 3 | 3 | 12040000 | 11456992 | 11988000 |
| Toll like receptor-2 protein | TLR-2 | 784 | 3 | 3 | 3 | 3 | 3 | 3 | 0 | 5361763 | 569980 |
| Transmembrane protein 263 | TMEM263 | 115 | 3 | 3 | 3 | 3 | 3 | 3 | 63369000 | 46412000 | 41969000 |
| Translationally-controlled tumor protein | TPT1 | 156 | 1 | 2 | 1 | 1 | 2 | 1 | 4999000 | 0 | 4977500 |
| Ubiquitin-60S ribosomal protein L40 | UBA52 | 128 | 3 | 2 | 3 | 3 | 2 | 3 | 313444803 | 33444803 | 34482000 |
| Ubiquitin domain containing 1 | UBTD1 | 204 | 3 | 3 | 3 | 3 | 3 | 3 | 22439669 | 0 | 2779800 |
| Urocortin-3 | UCN3 | 29 | 3 | 2 | 2 | 1 | 2 | 1 | 0 | 5366295 | 5586600 |
| UTP--glucose-1-phosphate uridylyltransferase | UGP2 | 504 | 5 | 4 | 4 | 5 | 4 | 4 | 102678 | 107069 | 109042 |
| Transitional endoplasmic reticulum ATPase | VCP | 806 | 2 | 2 | 2 | 2 | 2 | 2 | 5404700 | 4443000 | 5272200 |
| Vanin 2 | VNN2 | 467 | 2 | 2 | 2 | 2 | 2 | 2 | 2312300 | 2150000 | 2543500 |
| Xanthine dehydrogenase | XDH | 262 | 15 | 15 | 15 | 2 | 2 | 2 | 201280000 | 209520000 | 167320000 |
| Synaptobrevin homolog YKT6 | YKT6 | 198 | 1 | 2 | 1 | 1 | 2 | 1 | 0 | 4002800 | 4214000 |
| 14-3-3 protein beta/alpha | YWHAB | 246 | 3 | 3 | 3 | 2 | 2 | 3 | 15441000 | 13979000 | 15283000 |
| 14-3-3 protein epsilon | YWHAE | 235 | 2 | 2 | 1 | 2 | 1 | 1 | 7071690 | 0 | 7852100 |
| 14-3-3 protein gamma | YWHAG | 225 | 3 | 4 | 3 | 3 | 4 | 4 | 24430000 | 25879000 | 23299000 |
| 14-3-3 protein theta | YWHAQ | 245 | 2 | 2 | 1 | 1 | 2 | 1 | 7957000 | 0 | 7922700 |
| Zinc finger protein 613 | ZNF613 | 644 | 3 | 3 | 3 | 1 | 3 | 3 | 103935 | 102852 | 100889 |
| BC |  |  |  |  |  |  |  |  |  |  |  |
| Alpha-1B-glycoprotein | A1BG | 586 | 4 | 4 | 4 | 4 | 4 | 4 | 66365000 | 90802000 | 83220000 |
| Alpha-2-macroglobulin | A2M | 1510 | 3 | 3 | 2 | 3 | 3 | 2 | 28887000 | 36841000 | 37184000 |
| ATP binding cassette subfamily B member 9 | ABCB9 | 770 | 6 | 6 | 6 | 4 | 4 | 4 | 14476879 | 1074490 | 0 |
| ATP binding cassette subfamily C member 12 | ABCC12 | 1355 | 5 | 4 | 4 | 5 | 4 | 4 | 0 | 6821931 | 7510979 |
| ATP-binding cassette sub-family G member 2 | ABCG2 | 655 | 4 | 4 | 4 | 2 | 2 | 2 | 0 | 656639 | 601950 |
| Actin, alpha 1, skeletal muscle | ACTA1 | 377 | 8 | 8 | 8 | 4 | 4 | 4 | 204130000 | 187870000 | 212090000 |
| Actin, cytoplasmic 1 | ACTB | 375 | 6 | 6 | 6 | 2 | 2 | 2 | 36583000 | 45895000 | 41752000 |
| Actin, cytoplasmic 2 | ACTG1 | 375 | 1 | 6 | 0 | 0 | 2 | 0 | 8044800 | 10581000 | 9953400 |
| Alpha-2-HS-glycoprotein | AHSG | 359 | 3 | 3 | 3 | 3 | 3 | 3 | 49545000 | 58069000 | 51649000 |
| Serum albumin | ALB | 607 | 6 | 19 | 2 | 6 | 19 | 2 | 24787000 | 332120000 | 31692000 |
| Fructose-bisphosphate aldolase | ALDOC | 364 | 2 | 2 | 2 | 2 | 2 | 2 | 0 | 1921016 | 1191300 |
| Alpha lactalbumin | alfaLA | 123 | 6 | 6 | 6 | 6 | 6 | 6 | 2211900000 | 2228000000 | 2416200000 |
| Annexin A2 | ANXA2 | 339 | 2 | 1 | 2 | 2 | 1 | 2 | 0 | 4798141 | 4698659 |
| APC regulator of WNT signaling pathway 2 | APC2 | 2253 | 6 | 6 | 6 | 6 | 6 | 6 | 6720800 | 0 | 7124800 |
| Apolipoprotein A-I | APOA1 | 265 | 7 | 6 | 7 | 7 | 6 | 7 | 124950000 | 152080000 | 139690000 |
| Apolipoprotein A-II | APOA2 | 100 | 2 | 1 | 1 | 2 | 1 | 1 | 0 | 5972228 | 5276094 |
| Apolipoprotein A-IV | APOA4 | 378 | 0 | 2 | 0 | 0 | 2 | 0 | 9543200 | 10075000 | 10333000 |
| Apolipoprotein E | APOE | 316 | 3 | 4 | 3 | 3 | 4 | 3 | 72674000 | 81129000 | 81734000 |
| Rho GTPase activating protein 31 | ARHGAP31 | 1312 | 2 | 3 | 3 | 2 | 3 | 3 | 12570000 | 0 | 13326000 |
| Calcium-transporting ATPase | ATP2B3 | 1173 | 4 | 4 | 3 | 4 | 4 | 3 | 0 | 5023212 | 7082967 |
| Zinc-alpha-2-glycoprotein | AZGP1 | 299 | 6 | 6 | 6 | 6 | 6 | 6 | 63210000 | 74496000 | 64892000 |
| Beta-2-microglobulin | B2M | 118 | 1 | 1 | 2 | 1 | 1 | 2 | 0 | 13773000 | 12148920 |
| Beta-1,4-galactosyltransferase 1 | B4GALT1 | 402 | 4 | 4 | 4 | 4 | 4 | 4 | 72031000 | 80967000 | 78481000 |
| BRCA1 associated RING domain 1 | BARD1 | 773 | 3 | 4 | 3 | 3 | 4 | 3 | 3165978 | 4429522 | 0 |
| Biglycan | BGN | 369 | 5 | 5 | 5 | 4 | 4 | 4 | 5007938 | 5428033 | 0 |
| MHC class I antigen | BoLA | 360 | 2 | 3 | 2 | 1 | 1 | 1 |  | 10424052 | 10096114 |
| BPI fold containing family B member 6 | BPIFB6 | 453 | 8 | 8 | 9 | 8 | 8 | 9 | 6463965 | 6333835 | 0 |
| Butyrophilin subfamily 1 member A1 | BTN1A1 | 526 | 10 | 10 | 10 | 10 | 10 | 10 | 1081200000 | 989510000 | 1276000000 |
| Complement 3 | C3 | 1661 | 26 | 26 | 26 | 2 | 2 | 2 | 622540000 | 691600000 | 756600000 |
| Complement 4 | C4 | 920 | 2 | 3 | 2 | 2 | 3 | 2 | 0 | 6745500 | 5991800 |
| Coiled-coil domain containing 112 | CCDC112 | 523 | 2 | 3 | 3 | 2 | 1 | 1 | 13048437 | 14106522 | 13551827 |
| Coiled-coil domain containing 18 | CCDC18 | 1456 | 3 | 3 | 3 | 3 | 3 | 3 | 0 | 5571394 | 6415858 |
| Monocyte differentiation antigen CD14 | CD14 | 373 | 3 | 3 | 3 | 3 | 3 | 3 | 2617800 | 2991700 | 2746700 |
| Platelet glycoprotein 4 | CD36 | 472 | 2 | 2 | 2 | 2 | 2 | 2 | 9133503 | 9309321 | 0 |
| CD5 molecule like | CD5L | 451 | 2 | 2 | 3 | 2 | 2 | 3 | 0 | 3266898 | 2334200 |
| Complement factor B | CFB | 725 | 0 | 2 | 0 | 0 | 2 | 0 | 2361800 | 3131600 | 2501900 |
| Creatine kinase M-type | CKM | 388 | 4 | 5 | 4 | 4 | 5 | 4 | 27681000 | 38144000 | 35005000 |
| Cleft lip and palate transmembrane protein 1 homolog | CLPTM1 | 670 | 5 | 5 | 5 | 4 | 4 | 4 | 0 | 1258853 | 178080 |
| Clusterin | CLU | 439 | 2 | 9 | 0 | 2 | 9 | 0 | 50406000 | 50383000 | 58009000 |
| Clustered mitochondria protein homolog | CLUH | 1349 | 1 | 2 | 2 | 1 | 2 | 2 | 4163700 | 0 | 4414000 |
| Carboxypeptidase Q | CPQ | 472 | 2 | 2 | 1 | 2 | 2 | 1 | 9836800 | 12509000 | 12066000 |
| CUB and Sushi multiple domains 2 | CSMD2 | 3299 | 7 | 7 | 7 | 3 | 3 | 3 | 14513541 | 14791099 | 0 |
| Alpha-S1-casein | CSN1S1 | 200 | 10 | 10 | 10 | 10 | 10 | 10 | 15274000000 | 14183000000 | 13097000000 |
| Alpha-S2-casein | CSN1S2 | 222 | 6 | 6 | 5 | 6 | 6 | 5 | 1644300000 | 2016800000 | 1619600000 |
| Beta-casein | CSN2 | 259 | 2 | 3 | 3 | 2 | 3 | 3 | 216060000 | 610200000 | 240730000 |
| Kappa-casein | CSN3 | 136 | 2 | 2 | 1 | 2 | 2 | 1 | 185950000 | 168880000 | 31473000 |
| Cystatin-C | CST3 | 148 | 2 | 3 | 3 | 2 | 3 | 3 | 11978368 | 11091688 | 0 |
| Cystatin E/M | CST6 | 149 | 2 | 2 | 2 | 2 | 2 | 2 | 0 | 301976 | 466150 |
| Dipeptidyl peptidase like 10 | DPP10 | 675 | 2 | 2 | 2 | 2 | 2 | 2 | 266940000 | 0 | 282980000 |
| Elongation factor 1-alpha 1 | EEF1A1 | 462 | 2 | 2 | 1 | 2 | 2 | 1 | 21049000 | 20951000 | 23257000 |
| EF-hand calcium binding domain 11 | EFCAB11 | 205 | 3 | 3 | 3 | 3 | 3 | 3 | 82076641 | 78756420 | 0 |
| Receptor protein-tyrosine kinase | EGFR | 1203 | 2 | 2 | 2 | 2 | 2 | 2 | 6694900 | 6817100 | 6758400 |
| Alpha-enolase | ENO1 | 434 | 2 | 2 | 2 | 2 | 2 | 2 | 13901297 | 14549903 | 13107864 |
| Beta-enolase | ENO3 | 412 | 2 | 2 | 2 | 2 | 2 | 2 | 6438160 | 6150652 | 0 |
| EPH receptor A3 | EPHA3 | 984 | 3 | 3 | 3 | 3 | 3 | 3 | 14029807 | 14290268 | 0 |
| Ezrin | EZR | 670 | 5 | 5 | 5 | 5 | 5 | 5 | 10776000 | 11950000 | 11393000 |
| Fatty acid-binding protein, heart | FABP3 | 133 | 2 | 3 | 0 | 2 | 3 | 0 | 10517000 | 12829000 | 9150800 |
| Fatty acid synthase | FASN | 2474 | 2 | 2 | 2 | 2 | 2 | 2 | 6673300 | 0 | 4952700 |
| Fibrinogen alpha chain | FGA | 615 | 7 | 7 | 7 | 7 | 7 | 7 | 13943000 | 0 | 14781000 |
| Fibrinogen beta chain | FGB | 468 | 4 | 4 | 3 | 4 | 4 | 3 | 43508000 | 49217000 | 48298000 |
| Fibrinogen gamma-B chain | FGG | 426 | 2 | 2 | 2 | 2 | 2 | 2 | 22384000 | 28632000 | 22007000 |
| Fibronectin | FN1 | 320 | 3 | 3 | 3 | 3 | 3 | 3 | 8201400 | 0 | 8694400 |
| Folate receptor alpha | FOLR1 | 241 | 3 | 3 | 3 | 3 | 3 | 3 | 50109000 | 103790000 | 95478000 |
| Uncharacterized protein | FOLR3 | 287 | 0 | 3 | 0 | 0 | 2 | 0 | 22306000 | 22314000 | 23375000 |
| Glyceraldehyde-3-phosphate dehydrogenase | GAPDH | 227 | 3 | 2 | 2 | 3 | 2 | 2 | 11005000 | 12371000 | 11868000 |
| Vitamin D-binding protein | GC | 474 | 3 | 3 | 4 | 3 | 3 | 4 | 182970000 | 131200000 | 205700000 |
| Vitamin K-dependent gamma-carboxylase | GGCX | 758 | 4 | 4 | 3 | 4 | 4 | 3 | 0 | 1575300 | 1754285 |
| Myoglobin | MB | 154 | 3 | 3 | 3 | 3 | 3 | 3 | 10514000 | 10106000 | 11040000 |
| Glycosylation-dependent cell adhesion molecule 1 | GLYCAM1 | 153 | 3 | 2 | 3 | 3 | 2 | 3 | 168140000 | 268750000 | 97929000 |
| Glycoprotein 2 | GP2 | 534 | 14 | 14 | 15 | 14 | 14 | 15 | 1723822 | 0 | 1487700 |
| G protein-coupled receptor class C group 5 member B | GPRC5B | 387 | 3 | 3 | 3 | 3 | 3 | 3 | 125239124 | 132450684 | 130970741 |
| Gelsolin | GSN | 731 | 5 | 5 | 4 | 5 | 5 | 4 | 32866000 | 33781000 | 36890000 |
| Histone H2B | H2B | 126 | 1 | 2 | 0 | 1 | 2 | 0 | 2775200 | 3197200 | 3434600 |
| Hemoglobin subunit alpha | HBA | 142 | 2 | 2 | 2 | 2 | 2 | 2 | 5839011 | 6145129 | 0 |
| GLOBIN domain-containing protein | HBA1 | 136 | 2 | 2 | 2 | 2 | 2 | 2 | 11191686 | 10080221 | 14627258 |
| Tyrosine-protein kinase | HCK | 558 | 7 | 8 | 8 | 7 | 8 | 8 | 0 | 3915072 | 3178471 |
| Heart fatty acid-binding protein | H-FABP | 133 | 3 | 3 | 3 | 3 | 3 | 3 | 45364000 | 55024000 | 43581000 |
| HHIP like 2 | HHIPL2 | 766 | 1 | 1 | 2 | 1 | 1 | 2 | 7140629 | 7866680 | 0 |
| Histone H4 | His4 | 136 | 3 | 3 | 3 | 3 | 3 | 3 | 15251000 | 21806000 | 19969000 |
| Heterogeneous nuclear ribonucleoprotein K | HNRNPK | 437 | 5 | 5 | 4 | 5 | 5 | 4 | 0 | 9641202 | 975500 |
| Hemopexin | HPX | 459 | 2 | 1 | 1 | 2 | 1 | 1 | 22991000 | 25847000 | 27602000 |
| IGFBP7 protein | IGFBP7 | 282 | 2 | 2 | 2 | 2 | 2 | 2 | 2724600 | 2730400 | 3852800 |
| IGK protein | IGK | 240 | 0 | 4 | 0 | 0 | 4 | 0 | 22261000 | 15257000 | 17499000 |
| IGL@ protein | IGL@ | 235 | 2 | 2 | 0 | 0 | 2 | 0 | 4624746 | 0 | 4022500 |
| Inter-alpha-trypsin inhibitor heavy chain H2 | ITIH2 | 864 | 0 | 2 | 0 | 0 | 2 | 0 | 0 | 1417200 | 1335045 |
| Inter-alpha-trypsin inhibitor heavy chain H4 | ITIH4 | 916 | 2 | 2 | 1 | 2 | 2 | 1 | 13645013 | 11777902 | 13427100 |
| Immunoglobulin J chain | JCHAIN | 157 | 3 | 3 | 3 | 3 | 3 | 3 | 219210000 | 120680000 | 219310000 |
| KAT8 regulatory NSL complex subunit 1 | KANSL1 | 1103 | 3 | 3 | 3 | 3 | 3 | 3 | 7198158 | 9458207 | 9279536 |
| Kit ligand | KITLG | 274 | 14 | 13 | 14 | 14 | 13 | 14 | 2235621 | 1645411 | 2144100 |
| Keratin 1 | KRT1 | 606 | 13 | 14 | 14 | 9 | 10 | 10 | 1356500000 | 952960000 | 1393600000 |
| Keratin, type I cytoskeletal 10 | KRT10 | 526 | 14 | 13 | 13 | 11 | 10 | 10 | 343020000 | 375850000 | 391890000 |
| Keratin, type I cytoskeletal 14 | KRT14 | 458 | 3 | 4 | 0 | 3 | 3 | 0 | 3573600 | 2269400 | 0 |
| Keratin, type I cytoskeletal 16 | KRT16 | 473 | 4 | 4 | 4 | 1 | 2 | 3 | 0 | 10746656 | 12060177 |
| Keratin, type I cytoskeletal 18 | Krt18 | 423 | 16 | 17 | 15 | 15 | 16 | 14 | 630440000 | 763310000 | 698430000 |
| Keratin, type II cytoskeletal 2 epidermal | KRT2 | 639 | 11 | 11 | 12 | 6 | 6 | 7 | 217480000 | 263030000 | 254370000 |
| Keratin 24 | KRT24 | 525 | 1 | 2 | 1 | 1 | 2 | 1 | 0 | 4238941 | 3032800 |
| Keratin, type II cytoskeletal 5 | KRT5 | 590 | 4 | 4 | 4 | 1 | 3 | 3 | 18568000 | 18576000 | 25778000 |
| Keratin, type II cytoskeletal 6C | KRT6C | 564 | 3 | 4 | 0 | 2 | 3 | 0 | 3418200 | 3330266 | 0 |
| Keratin, type II cytoskeletal 7 | KRT7 | 466 | 18 | 15 | 4 | 15 | 11 | 3 | 416960000 | 368900000 | 332290000 |
| Keratin, type II cytoskeletal 71 | Krt71 | 524 | 11 | 10 | 9 | 6 | 5 | 8 | 243510000 | 219320000 | 263620000 |
| Keratin, type II cytoskeletal 79 | KRT79 | 535 | 8 | 13 | 0 | 6 | 8 | 0 | 82931000 | 98268000 | 91476000 |
| Keratin, type I cytoskeletal 9 | KRT9 | 623 | 5 | 17 | 0 | 5 | 16 | 0 | 133150000 | 150270000 | 130070000 |
| Kinase suppressor of ras 2 | KSR2 | 950 | 0 | 1 | 0 | 0 | 1 | 0 | 6063118 | 6737292 | 7339666 |
| Alpha-lactalbumin | LALBA | 142 | 6 | 6 | 4 | 6 | 6 | 4 | 3191500000 | 4292400000 | 1675000000 |
| Lipopolysaccharide-binding protein | LBP | 481 | 2 | 2 | 2 | 2 | 2 | 2 | 15087000 | 14392000 | 22584000 |
| Lipocalin 2 | LCN2 | 200 | 12 | 13 | 12 | 12 | 13 | 12 | 9540693 | 9284256 | 0 |
| L-lactate dehydrogenase A chain | LDHA | 332 | 3 | 3 | 3 | 1 | 1 | 1 | 2597408 | 2689078 | 0 |
| L-lactate dehydrogenase B chain | LDHB | 334 | 4 | 4 | 4 | 4 | 4 | 4 | 0 | 1044833 | 1263500 |
| Beta-lactoglobulin | LGB | 178 | 6 | 7 | 7 | 3 | 4 | 4 | 0 | 13015613 | 13208000 |
| Ubiquitin-like domain-containing protein | LOC101902760 | 77 | 1 | 2 | 0 | 1 | 2 | 0 | 4413800 | 5020000 | 3423000 |
| Histone H2A | H2AFV | 86 | 5 | 6 | 6 | 1 | 2 | 2 | 0 | 14989995 | 13015613 |
| Serotransferrin-like | LOC525947 | 622 | 2 | 2 | 2 | 2 | 2 | 2 | 32040000 | 35875000 | 39707000 |
| IGc1 domain-containing protein | LOC782529 | 334 | 6 | 6 | 6 | 6 | 6 | 6 | 12298208 | 12688106 | 13445269 |
| B30.2/SPRY domain-containing protein | LOC786706 | 316 | 2 | 3 | 3 | 1 | 1 | 1 | 0 | 10366777 | 10829000 |
| Lysyl oxidase like 3 | LOXL3 | 752 | 7 | 7 | 7 | 2 | 2 | 2 | 0 | 4938874 | 4028730 |
| Lipoprotein lipase | LPL | 478 | 17 | 17 | 17 | 17 | 17 | 17 | 3770608 | 3684055 | 2597408 |
| Lactoperoxidase | LPO | 731 | 6 | 6 | 5 | 6 | 6 | 5 | 88321000 | 92757000 | 96586000 |
| Leucine rich alpha-2-glycoprotein 1 | LRG1 | 353 | 2 | 2 | 2 | 2 | 2 | 2 | 15883000 | 16739000 | 18293000 |
| U6 snRNA-associated Sm-like protein LSm4 | LSM4 | 139 | 22 | 22 | 21 | 22 | 22 | 21 | 0 | 312400000 | 36840055 |
| Lactoferrin | LTF | 690 | 9 | 13 | 0 | 9 | 13 | 0 | 27359000 | 22296000 | 17981000 |
| Melanotransferrin | MELTF | 739 | 4 | 3 | 3 | 4 | 3 | 3 | 39221000 | 41344000 | 38885000 |
| Lactadherin | MFGE8 | 431 | 19 | 20 | 20 | 19 | 20 | 20 | 2048600000 | 1702700000 | 2049800000 |
| Myostatin | MSTN | 124 | 4 | 3 | 4 | 4 | 3 | 3 | 134830000 | 147350000 | 143230000 |
| Mitochondrial poly(A) polymerase | MTPAP | 583 | 8 | 8 | 8 | 6 | 6 | 6 | 0 | 312183 | 366860 |
| Myosin-1 | MYH1 | 1922 | 12 | 13 | 13 | 2 | 2 | 2 | 88598000 | 66785000 | 88656000 |
| Myosin-2 | MYH2 | 1940 | 12 | 10 | 12 | 2 | 2 | 10 | 55537000 | 72817000 | 57610000 |
| Myosin heavy chain 6 | MYH6 | 1938 | 7 | 8 | 6 | 6 | 6 | 6 | 9150300 | 7033700 | 11494000 |
| Myosin-7 | MYH7 | 1935 | 6 | 8 | 6 | 0 | 6 | 1 | 5032520 | 5802904 | 0 |
| Myosin heavy chain 7B | MYH7B | 1928 | 1 | 8 | 0 | 0 | 5 | 0 | 2507746 | 2442900 | 0 |
| Myosin heavy chain 8 | MYH8 | 1937 | 11 | 12 | 12 | 9 | 9 | 9 | 9274200 | 12095000 | 14589000 |
| MYL1 protein | MYL1 | 176 | 3 | 2 | 2 | 3 | 2 | 2 | 3106900 | 3537800 | 5606700 |
| Cytosolic Fe-S cluster assembly factor NARFL | NARFL | 476 | 21 | 21 | 20 | 20 | 20 | 19 | 6653773 | 6629816 | 0 |
| NLR family pyrin domain containing 12 | NLRP12 | 1054 | 16 | 16 | 16 | 14 | 14 | 14 | 11952606 | 14106536 | 11316021 |
| Nucleoside diphosphate kinase B | NME2 | 114 | 2 | 2 | 2 | 1 | 2 | 1 | 0 | 8574264 | 9295648 |
| Nucleobindin-1 | NUCB1 | 474 | 7 | 7 | 7 | 7 | 7 | 7 | 96480000 | 124150000 | 118830000 |
| Nucleobindin 2 | NUCB2 | 429 | 3 | 4 | 4 | 3 | 4 | 4 | 12501000 | 13831000 | 12193000 |
| Alpha-1-acid glycoprotein | ORM1 | 202 | 3 | 3 | 3 | 3 | 3 | 1 | 11862097 | 10466989 | 11151872 |
| Lipocln_cytosolic_FA-bd_dom domain-containing protein | PAEP | 178 | 13 | 14 | 13 | 1 | 2 | 1 | 5553200000 | 5227800000 | 5803800000 |
| Pericentrin | PCNT | 3085 | 4 | 5 | 5 | 4 | 5 | 5 | 0 | 578435700 | 533400000 |
| Profilin-1 | PFN1 | 140 | 2 | 2 | 1 | 2 | 2 | 1 | 0 | 14106536 | 14831339 |
| Profilin-2 | PFN2 | 140 | 5 | 5 | 5 | 5 | 1 | 1 | 0 | 1731018 | 16774183 |
| PIGR protein | PIGR | 757 | 7 | 8 | 8 | 7 | 8 | 8 | 170090000 | 195310000 | 205460000 |
| Peptidyl-prolyl cis-trans isomerase NIMA-interacting 4 | PIN4 | 131 | 3 | 3 | 3 | 3 | 3 | 3 | 29633000 | 26744000 | 19254000 |
| 1-phosphatidylinositol 4,5-bisphosphate phosphodiesterase gamma | PLCG2 | 1262 | 5 | 5 | 5 | 2 | 2 | 2 | 2 | 14688000 | 10688000 |
| Perilipin-2 | PLIN2 | 447 | 4 | 4 | 3 | 4 | 4 | 3 | 46059000 | 48453000 | 53509000 |
| Perilipin-3 | PLIN3 | 438 | 0 | 3 | 0 | 0 | 3 | 0 | 3674700 | 3307200 | 2828000 |
| Serine/threonine-protein kinase PLK | PLK3 | 647 | 3 | 3 | 3 | 1 | 2 | 1 | 5662071 | 0 | 669540 |
| Polyamine modulated factor 1 binding protein 1 | PMFBP1 | 997 | 2 | 2 | 2 | 2 | 2 | 2 | 0 | 8728280 | 8300749 |
| Peptidyl-prolyl cis-trans isomerase B | PPIB | 216 | 0 | 3 | 0 | 0 | 3 | 0 | 4264100 | 4744900 | 5677400 |
| Peroxiredoxin-4 | PRDX4 | 260 | 2 | 2 | 2 | 2 | 2 | 2 | 3370100 | 0 | 3572600 |
| Serine protease 8 | PRSS8 | 343 | 2 | 2 | 1 | 2 | 2 | 1 | 14112000 | 8447000 | 13316000 |
| Glutamine-rich protein 1 | QRICH1 | 779 | 3 | 3 | 3 | 2 | 2 | 2 | 15899000 | 0 | 16855000 |
| Ras-related protein Rab-18 | RAB18 | 206 | 1 | 3 | 0 | 1 | 3 | 0 | 0 | 2291189 | 3347681 |
| RAS guanyl releasing protein 1 | RASGRP1 | 797 | 3 | 3 | 3 | 3 | 3 | 3 | 12866619 | 12616887 | 11705975 |
| Ras association domain-containing protein 3 | RASSF3 | 235 | 4 | 4 | 4 | 2 | 2 | 2 | 0 | 3844215 | 3031230 |
| 40S ribosomal protein SA | RPSA | 295 | 5 | 4 | 4 | 2 | 2 | 2 | 6229598 | 7951267 | 7646660 |
| Serum amyloid A protein | SAA3 | 131 | 2 | 2 | 2 | 2 | 2 | 1 | 97874000 | 0 | 103760000 |
| 45 kDa calcium-binding protein | SDF4 | 355 | 2 | 2 | 2 | 2 | 2 | 2 | 4760300 | 4693200 | 5978700 |
| Alpha-1-antiproteinase | SERPINA1 | 416 | 1 | 3 | 0 | 1 | 3 | 0 | 14112000 | 11394000 | 11462000 |
| Serpin A3-1 | SERPINA3-1 | 400 | 6 | 6 | 6 | 2 | 2 | 6 | 155410000 | 167960000 | 190030000 |
| Serpin A3-2 | SERPINA3-2 | 411 | 1 | 6 | 0 | 0 | 2 | 0 | 26951000 | 52022000 | 46221000 |
| Antithrombin-III | SERPINC1 | 530 | 2 | 2 | 2 | 2 | 2 | 2 | 9724000 | 10371000 | 15600000 |
| Pigment epithelium-derived factor | SERPINF1 | 416 | 2 | 2 | 2 | 2 | 2 | 2 | 13751910 | 13400483 | 14124735 |
| Alpha-2-antiplasmin | SERPINF2 | 492 | 15 | 14 | 15 | 12 | 12 | 12 | 0 | 7441195 | 7542634 |
| Nucleotide exchange factor SIL1 | SIL1 | 462 | 8 | 8 | 8 | 6 | 6 | 6 | 8300749 | 8008316 | 8589771 |
| Sodium/nucleoside cotransporter | SLC28A3 | 697 | 7 | 6 | 6 | 2 | 2 | 2 | 0 | 5662071 | 5750116 |
| Sodium-dependent phosphate transport protein 2B | SLC34A2 | 693 | 11 | 11 | 11 | 10 | 10 | 10 | 0 | 1412474 | 1274100 |
| Osteopontin | SPP1 | 278 | 8 | 8 | 8 | 6 | 6 | 6 | 4261759 | 4916100 | 4950760 |
| Stromal antigen 1 | STAG1 | 998 | 6 | 6 | 6 | 6 | 6 | 6 | 0 | 5506833 | 5953900 |
| Stromal antigen 2 | STAG2 | 1268 | 2 | 2 | 2 | 2 | 2 | 2 | 1195658 | 1130363 | 1158466 |
| Spermatid perinuclear RNA-binding protein | STRBP | 657 | 2 | 2 | 2 | 1 | 2 | 1 | 0 | 5574254 | 5717626 |
| Tet methylcytosine dioxygenase 3 | TET3 | 1699 | 4 | 4 | 4 | 3 | 3 | 3 | 74349000 | 142050000 | 120200000 |
| Serotransferrin | TF | 704 | 6 | 6 | 6 | 6 | 6 | 6 | 108070000 | 138220000 | 94446000 |
| Thrombospondin-1 | THBS1 | 1170 | 8 | 9 | 8 | 8 | 9 | 8 | 164650000 | 158400000 | 176470000 |
| Transmembrane protein 237 | TMEM237 | 400 | 3 | 3 | 3 | 2 | 3 | 2 | 0 | 1160406 | 1138200 |
| Tubulin tyrosine ligase like 6 | TTLL6 | 742 | 21 | 21 | 21 | 19 | 20 | 19 | 0 | 9272784 | 8459027 |
| Ubiquitin-60S ribosomal protein L40 | UBA52 | 128 | 2 | 2 | 2 | 2 | 2 | 2 | 23050000 | 15801000 | 27022000 |
| U3 small nucleolar RNA-associated protein 15 homolog | UTP15 | 519 | 2 | 2 | 1 | 2 | 2 | 1 | 0 | 14212035 | 13356902 |
| Xanthine dehydrogenase/oxidase | XDH | 1330 | 15 | 15 | 15 | 14 | 14 | 15 | 241620000 | 234990000 | 277670000 |
| 14-3-3 protein eta | YWHAH | 220 | 8 | 8 | 8 | 1 | 3 | 3 | 7542140 | 9542140 | 7942140 |
| Z-DNA binding protein 1 | ZBP1 | 401 | 6 | 6 | 6 | 6 | 6 | 6 | 2839505 | 3286594 | 3436909 |
| Zinc finger protein 777 | ZNF777 | 794 | 2 | 2 | 2 | 2 | 2 | 2 | 0 | 55006833 | 50884000 |
| BM |  |  |  |  |  |  |  |  |  |  |  |
| [F-actin]-monooxygenase | MICAL2 | 1101 | 5 | 6 | 5 | 5 | 6 | 5 | 103541 | 103918 | 0 |
| 14-3-3 protein theta | YWHAQ | 127 | 9 | 8 | 9 | 7 | 6 | 7 | 1974300 | 0 | 2093000 |
| 60S ribosomal protein L8 | RPL8 | 257 | 2 | 2 | 2 | 2 | 2 | 2 | 0 | 13449000 | 13813318 |
| Actin, alpha skeletal muscle | ACTA1 | 377 | 8 | 8 | 8 | 4 | 4 | 8 | 152650000 | 189980000 | 162960000 |
| Actin, aortic smooth muscle | ACTA2 | 377 | 2 | 2 | 2 | 2 | 2 | 2 | 18883000 | 13670000 | 13612000 |
| Actin, cytoplasmic 1 | ACTB | 375 | 5 | 5 | 1 | 5 | 5 | 1 | 34887000 | 0 | 36984000 |
| Actin, cytoplasmic 2 | ACTG1 | 375 | 5 | 3 | 4 | 5 | 3 | 4 | 7347200 | 5099800 | 5132400 |
| Actin, gamma-enteric smooth muscle | ACTG2 | 376 | 6 | 5 | 5 | 6 | 5 | 5 | 27387000 | 26016000 | 22300000 |
| Actin-related protein 2 | ACTR2 | 394 | 5 | 4 | 5 | 5 | 4 | 5 | 1327043 | 1251327 | 1311700 |
| Acyl-CoA synthetase long chain family member 1 | ACSL1 | 660 | 2 | 2 | 2 | 2 | 2 | 2 | 7347400 | 6755800 | 6716100 |
| Adenosine deaminase RNA specific B1 | ADARB1 | 721 | 21 | 22 | 22 | 21 | 22 | 22 | 4891699 | 4880067 | 4318800 |
| Adenosine monophosphate deaminase 1 | AMPD1 | 72 | 6 | 6 | 5 | 6 | 6 | 5 | 0 | 198717 | 190850 |
| Alpha lactalbumin | alfaLA | 123 | 12 | 10 | 12 | 12 | 10 | 12 | 56259000 | 61189000 | 63992000 |
| Alpha/beta hydrolase domain-containing protein 17C | ABHD17C | 329 | 2 | 2 | 2 | 2 | 2 | 2 | 7577593 | 7617536 | 7150400 |
| Alpha-1-antiproteinase | SERPINA1 | 416 | 3 | 3 | 3 | 3 | 3 | 3 | 47641000 | 61345000 | 50911000 |
| Alpha-1B-glycoprotein | A1BG | 503 | 4 | 4 | 4 | 4 | 4 | 4 | 83959000 | 63359000 | 90029000 |
| Alpha-2-antiplasmin | SERPINF2 | 492 | 20 | 20 | 20 | 17 | 17 | 0 | 7367300 | 0 | 7810200 |
| Alpha-2-HS-glycoprotein | AHSG | 359 | 21 | 22 | 22 | 2 | 2 | 1 | 0 | 3700634 | 3745700 |
| Alpha-2-macroglobulin | A2M | 1198 | 2 | 2 | 2 | 2 | 2 | 2 | 31531000 | 26676000 | 33754000 |
| Alpha-actinin-2 | ACTN2 | 894 | 4 | 4 | 4 | 4 | 4 | 4 | 882866 | 826324 | 899190 |
| Alpha-S1-casein | CSN1S1 | 214 | 10 | 10 | 10 | 2 | 2 | 10 | 10993000000 | 12107000000 | 12065000000 |
| Alpha-S2-casein [Cleaved into: Casocidin-1 | CSN1S2 | 222 | 2 | 2 | 2 | 2 | 2 | 2 | 205920000 | 117090000 | 126990000 |
| Annexin A1 | ANXA1 | 346 | 7 | 7 | 7 | 7 | 7 | 7 | 6333929 | 6101100 | 6589056 |
| Annexin A5 | ANXA5 | 321 | 6 | 5 | 5 | 6 | 5 | 5 | 0 | 4386372 | 3047000 |
| Antithrombin-III | SERPINC1 | 429 | 14 | 14 | 13 | 14 | 14 | 13 | 5807300 | 5235404 | 6156500 |
| APC regulator of WNT signaling pathway 2 | APC2 | 2253 | 7 | 7 | 8 | 7 | 7 | 8 | 6720800 | 7092663 | 7124800 |
| Apolipoprotein A-I preproprotein | APOA1 | 265 | 7 | 7 | 7 | 7 | 7 | 7 | 132010000 | 112970000 | 143820000 |
| Apolipoprotein A-II | APOA2 | 100 | 4 | 2 | 3 | 1 | 1 | 3 | 2342700 | 0 | 2483500 |
| Apolipoprotein A-IV | APOA4 | 378 | 2 | 2 | 2 | 2 | 2 | 2 | 10011000 | 9615200 | 10072000 |
| Apolipoprotein E | APOE | 237 | 6 | 6 | 6 | 0 | 2 | 2 | 1187531 | 1014401 | 1371100 |
| Arm_2 domain-containing protein | ARMCX4 | 2317 | 23 | 23 | 23 | 23 | 23 | 23 | 128703 | 140631 | 119188 |
| ATOH1 protein | ATOH1 | 352 | 7 | 7 | 7 | 7 | 7 | 7 | 24846463 | 24183953 | 25219000 |
| ATP binding cassette subfamily B member 9 | ABCB9 | 727 | 18 | 20 | 19 | 18 | 20 | 19 | 117605 | 110553 | 100062 |
| ATP binding cassette subfamily C member 12 | ABCC12 | 1355 | 20 | 20 | 20 | 13 | 13 | 13 | 551750 | 534271 | 474473 |
| ATP synthase subunit alpha, mitochondrial | ATP5F1A | 553 | 6 | 6 | 6 | 2 | 2 | 2 | 11401739 | 1273900 | 1168300 |
| ATP-binding cassette sub-family G member 2 | ABCG2 | 655 | 23 | 23 | 23 | 23 | 23 | 23 | 5101133 | 6012488 | 5567600 |
| B30.2/SPRY domain-containing protein | LOC786706 | 316 | 4 | 3 | 3 | 3 | 2 | 2 | 14886074 | 1579064 | 1585000 |
| Beta lactoglobulin D | bLG | 57 | 4 | 5 | 4 | 4 | 5 | 4 | 4786666 | 4905581 | 4431100 |
| Beta-1,4-galactosyltransferase 1 | B4GALT1 | 402 | 4 | 4 | 4 | 4 | 4 | 4 | 74612000 | 71240000 | 79274000 |
| Beta-2-glycoprotein 1 | APOH | 345 | 2 | 2 | 2 | 1 | 2 | 1 | 9257200 | 9602958 | 9813700 |
| Beta-2-microglobulin | B2M | 93 | 2 | 1 | 2 | 2 | 1 | 1 | 5367200 | 5820111 | 5689900 |
| Beta-casein | CSN2 | 77 | 3 | 3 | 3 | 3 | 3 | 3 | 1017500000 | 379840000 | 1082100000 |
| Beta-enolase | ENO3 | 412 | 2 | 1 | 1 | 2 | 1 | 1 | 8734200 | 0 | 9259200 |
| Beta-lactoglobulin | LGB | 47 | 2 | 2 | 1 | 0 | 2 | 1 | 22519000000 | 25284000000 | 23501000000 |
| Biglycan | BGN | 369 | 6 | 6 | 6 | 6 | 6 | 6 | 3699500 | 3921359 | 3921900 |
| BRCA1 associated RING domain 1 | BARD1 | 773 | 5 | 5 | 5 | 5 | 5 | 5 | 126330000 | 128500020 | 133920000 |
| BTB domain containing 7 | BTBD7 | 1125 | 10 | 11 | 10 | 10 | 11 | 10 | 119835 | 170376 | 153422 |
| Butyrophilin subfamily 1 member A1 | BTN1A1 | 526 | 11 | 10 | 11 | 11 | 10 | 11 | 320600000 | 299950000 | 350380000 |
| Calcium-transporting ATPase | ATP2B3 | 941 | 4 | 4 | 4 | 4 | 4 | 4 | 705100 | 775307 | 0 |
| Calponin-homology | LRCH2 | 738 | 20 | 20 | 19 | 20 | 20 | 19 | 14993649 | 14424695 | 14916000 |
| Carboxypeptidase Q | CPQ | 452 | 2 | 2 | 2 | 2 | 2 | 2 | 11947000 | 10227000 | 12903000 |
| Caspase recruitment domain protein 9 | CARD9 | 534 | 3 | 3 | 3 | 3 | 3 | 3 | 0 | 164658 | 174281 |
| Caspase-4 | CASP4 | 377 | 6 | 6 | 6 | 6 | 6 | 6 | 4826573 | 5225729 | 4120100 |
| Cathelicidin-1 | CATHL1A | 155 | 4 | 4 | 4 | 4 | 4 | 4 | 0 | 1199576 | 1199300 |
| CD5 molecule like | CD5L | 451 | 2 | 2 | 2 | 2 | 2 | 2 | 4284021 | 4143218 | 4636800 |
| Centrosomal protein 104 | CEP104 | 925 | 1 | 1 | 2 | 1 | 1 | 2 | 389690 | 303557 | 381360 |
| Chitinase-3-like protein 1 | CHI3L1 | 383 | 6 | 6 | 6 | 6 | 6 | 6 | 3621000 | 0 | 3838700 |
| Cholinergic receptor nicotinic alpha 6 subunit | CHRNA6 | 494 | 3 | 3 | 3 | 3 | 3 | 3 | 17359482 | 19425176 | 18654000 |
| Chromodomain helicase DNA binding protein 3 | CHD3 | 1972 | 2 | 2 | 2 | 2 | 2 | 2 | 1989787 | 1979415 | 1950700 |
| CKLF-like MARVEL transmembrane domain containing 7 | CMTM7 | 174 | 4 | 4 | 4 | 4 | 4 | 4 | 3315418 | 3355352 | 3429300 |
| Clustered mitochondria protein homolog | CLUH | 1349 | 1 | 1 | 2 | 1 | 1 | 2 | 4163700 | 4495135 | 4414000 |
| Clusterin | CLU | 439 | 9 | 9 | 9 | 9 | 9 | 9 | 212830000 | 211510000 | 232510000 |
| Coiled-coil domain containing 112 | CCDC112 | 523 | 5 | 6 | 6 | 5 | 6 | 6 | 15441000 | 0 | 16369000 |
| Coiled-coil domain containing 18 | CCDC18 | 1456 | 2 | 1 | 2 | 2 | 1 | 2 | 10062000 | 13482644 | 12254445 |
| Complement component 3 | C3 | 1661 | 23 | 22 | 23 | 1 | 1 | 22 | 581380000 | 564160000 | 643670000 |
| Complement factor B | CFB | 725 | 1 | 1 | 2 | 1 | 1 | 2 | 8541800 | 0 | 9055300 |
| Corneodesmosin | CDSN | 559 | 17 | 16 | 16 | 17 | 16 | 16 | 3529682 | 3826482 | 3690200 |
| Creatine kinase M-type | CKM | 381 | 4 | 4 | 4 | 4 | 4 | 4 | 20952000 | 27019000 | 23491000 |
| C-type lectin domain-containing protein | LOC101123029 | 212 | 2 | 1 | 1 | 2 | 1 | 1 | 4355700 | 4450026 | 0 |
| CUB and Sushi multiple domains 2 | CSMD2 | 3299 | 8 | 8 | 7 | 8 | 8 | 7 | 107680 | 132209 | 124950 |
| Homeobox protein cut-like | CUX1 | 1534 | 3 | 3 | 3 | 3 | 3 | 3 | 0 | 5411168 | 5578800 |
| Cystatin-C | CST3 | 148 | 2 | 1 | 2 | 2 | 1 | 2 | 4051500 | 4539824 | 4295000 |
| Cysteine-rich DPF motif domain-containing protein 1 | CDPF1 | 121 | 20 | 20 | 20 | 2 | 2 | 2 | 10757000 | 10619976 | 10527000 |
| Cytosolic Fe-S cluster assembly factor NARFL | NARFL | 476 | 3 | 2 | 3 | 3 | 2 | 3 | 116813 | 110135 | 168100 |
| Desmoglein-1 | DSG1 | 1043 | 2 | 3 | 3 | 2 | 3 | 3 | 0 | 2055872 | 2065200 |
| Desmoplakin | DSP | 2124 | 10 | 11 | 11 | 4 | 4 | 4 | 2850262 | 2849423 | 2847400 |
| Dipeptidyl peptidase like 10 | DPP10 | 675 | 5 | 6 | 6 | 5 | 6 | 6 | 266940000 | 211737550 | 282980000 |
| Disco interacting protein 2 homolog B | DIP2B | 1575 | 2 | 1 | 1 | 2 | 1 | 1 | 108922220 | 115111190 | 112790000 |
| DNA damage induced apoptosis suppressor | DDIAS | 918 | 3 | 2 | 3 | 3 | 2 | 3 | 142852 | 120926 | 145080 |
| DNA excision repair protein ERCC-6-like 2 | ERCC6L2 | 1558 | 4 | 4 | 4 | 4 | 4 | 4 | 0 | 7659700 | 8547400 |
| Enoyl-CoA hydratase domain containing 3 | ECHDC3 | 277 | 4 | 4 | 3 | 4 | 4 | 3 | 3444775 | 296523 | 345940 |
| Ezrin | EZR | 581 | 2 | 2 | 2 | 2 | 2 | 2 | 10444000 | 10975000 | 11337000 |
| FAD-binding PCMH-type domain-containing protein | AOX4 | 1327 | 1 | 2 | 2 | 1 | 2 | 2 | 0 | 2858228 | 2943500 |
| FAM75 domain-containing protein | C8H9orf131 | 1214 | 5 | 5 | 5 | 5 | 5 | 5 | 18499973 | 19892000 | 18325548 |
| Fatty acid synthase | FASN | 2470 | 3 | 3 | 3 | 3 | 3 | 3 | 14054000 | 12282000 | 12948000 |
| Fatty acid-binding protein, heart | FABP3 | 98 | 3 | 3 | 3 | 3 | 3 | 3 | 29344000 | 21806000 | 29695000 |
| FGB protein | FGB | 330 | 2 | 2 | 2 | 2 | 2 | 2 | 35644000 | 32098000 | 39019000 |
| Fibrinogen alpha chain [Cleaved into: Fibrinopeptide A; Fibrinogen alpha chain] | FGA | 615 | 9 | 8 | 9 | 9 | 8 | 9 | 13943000 | 0 | 14781000 |
| Fibrinogen gamma-B chain | FGG | 420 | 2 | 2 | 2 | 2 | 2 | 2 | 27195000 | 22832000 | 28558000 |
| Fibronectin | FN1 | 320 | 2 | 1 | 2 | 2 | 1 | 2 | 8201400 | 0 | 8694400 |
| Fibrous sheath interacting protein 2 | FSIP2 | 6749 | 3 | 3 | 3 | 3 | 3 | 3 | 0 | 11989909 | 11573000 |
| Folate receptor alpha | FOLR1 | 241 | 1 | 2 | 1 | 1 | 2 | 2 | 81389000 | 85835201 | 86282000 |
| Fructose-bisphosphate aldolase | ALDOC | 364 | 3 | 4 | 3 | 0 | 1 | 3 | 5146300 | 0 | 5455700 |
| G_PROTEIN_RECEP_F1_2 domain-containing protein | OR10G2 | 318 | 10 | 9 | 8 | 10 | 9 | 8 | 138135 | 129272 | 149459 |
| Gelsolin | GSN | 731 | 3 | 3 | 3 | 3 | 3 | 3 | 17870000 | 21849000 | 27851000 |
| GLOBIN domain-containing protein | HBA1 | 136 | 4 | 4 | 4 | 4 | 4 | 4 | 7880600 | 0 | 8354400 |
| Glucosidase 2 subunit beta | PRKCSH | 533 | 4 | 3 | 3 | 4 | 3 | 3 | 2261903 | 2614430 | 2974800 |
| Glutamine-rich protein 1 | QRICH1 | 779 | 2 | 2 | 2 | 2 | 2 | 2 | 17253205 | 0 | 18542752 |
| Glyceraldehyde-3-phosphate dehydrogenase | GAPDH | 227 | 3 | 3 | 3 | 3 | 3 | 3 | 14048000 | 12976000 | 15401000 |
| Glycoprotein 2 | GP2 | 525 | 2 | 2 | 2 | 2 | 2 | 2 | 15297000 | 19346000 | 19851000 |
| Glycosylation-dependent cell adhesion molecule 1 | GLYCAM1 | 153 | 2 | 2 | 2 | 2 | 2 | 2 | 9493100 | 6092700 | 6675800 |
| Growth factor receptor-bound protein 14 | GRB14 | 540 | 3 | 2 | 2 | 3 | 2 | 2 | 0 | 12679511 | 2639400 |
| Growth/differentiation factor 8 | MSTN | 124 | 4 | 3 | 4 | 4 | 3 | 3 | 134830000 | 147350000 | 143230000 |
| Guanine nucleotide-binding protein G | GNAS | 394 | 3 | 3 | 3 | 3 | 3 | 3 | 0 | 4709335 | 4596300 |
| Heat shock protein 90 beta | hsp90 | 234 | 2 | 2 | 2 | 2 | 2 | 2 | 338047 | 225951 | 253907 |
| Hemoglobin fetal subunit beta | HBB | 145 | 21 | 22 | 23 | 21 | 22 | 23 | 0 | 10487093 | 9354700 |
| Hemopexin | HPX | 425 | 3 | 3 | 4 | 3 | 3 | 4 | 17995000 | 0 | 19077000 |
| Heterogeneous nuclear ribonucleoprotein K | HNRNPK | 437 | 1 | 2 | 2 | 1 | 2 | 2 | 0 | 994561 | 903040 |
| HHIP like 2 | HHIPL2 | 766 | 1 | 2 | 1 | 1 | 2 | 1 | 13561 | 12697 | 12473 |
| Histone acetyltransferase | KAT2B | 799 | 8 | 8 | 7 | 5 | 5 | 5 | 0 | 117016 | 123730 |
| Histone cluster 1 H1 family member c | HIST1H1C | 227 | 13 | 14 | 14 | 13 | 14 | 14 | 4379651 | 5638706 | 5760000 |
| Histone H2A | H2AFV | 119 | 2 | 2 | 2 | 2 | 2 | 2 | 91174000 | 99206000 | 93695000 |
| Histone H3.3 | H3F3A | 136 | 2 | 2 | 1 | 2 | 2 | 1 | 23698000 | 17682000 | 0 |
| Histone H4 | HIS4 | 136 | 3 | 3 | 3 | 3 | 3 | 3 | 182910000 | 191970000 | 197490000 |
| HOXB3 protein | HOXB3 | 426 | 15 | 14 | 13 | 15 | 14 | 13 | 115319 | 109046 | 100985 |
| IARS protein | IARS | 1262 | 10 | 8 | 10 | 10 | 8 | 10 | 407330 | 0 | 398620 |
| IGK protein | IGK | 240 | 3 | 3 | 3 | 3 | 3 | 3 | 77489000 | 73059000 | 87042000 |
| IGL@ protein | IGL@ | 235 | 3 | 2 | 1 | 0 | 2 | 1 | 17377000 | 0 | 18422000 |
| Immunoglobulin J chain | JCHAIN | 157 | 6 | 7 | 7 | 6 | 7 | 7 | 0 | 6316864 | 5895900 |
| Junction plakoglobin | JUP | 741 | 5 | 5 | 6 | 5 | 5 | 6 | 109773 | 110810 | 121066 |
| Katanin p60 ATPase-containing subunit A1 | KATNAL1 | 364 | 8 | 7 | 7 | 8 | 7 | 7 | 0 | 1343006 | 138550 |
| K-casein | CSN3 | 132 | 2 | 2 | 2 | 2 | 2 | 2 | 158170000 | 191040000 | 162950000 |
| Keratin 24 | KRT24 | 525 | 2 | 2 | 2 | 2 | 2 | 2 | 8269300 | 10401000 | 5811400 |
| Keratin 3 | KRT3 | 616 | 4 | 4 | 3 | 2 | 2 | 0 | 1694302 | 1785475 | 1906700 |
| Keratin 42 | KRT42 | 453 | 4 | 4 | 4 | 1 | 4 | 4 | 0 | 6162539 | 5939300 |
| Keratin 77 | KRT77 | 548 | 4 | 3 | 3 | 3 | 1 | 3 | 12610409 | 1130563 | 1193400 |
| Keratin, type I cytoskeletal 10 | KRT10 | 584 | 19 | 19 | 18 | 14 | 14 | 13 | 307070000 | 316020000 | 305380000 |
| Keratin, type I cytoskeletal 13 | Krt13 | 437 | 8 | 8 | 6 | 7 | 7 | 5 | 248470000 | 252150000 | 245490000 |
| Keratin, type I cytoskeletal 14 | KRT14 | 472 | 5 | 5 | 5 | 2 | 2 | 2 | 4278300 | 4379500 | 4553300 |
| Keratin, type I cytoskeletal 16 | KRT16 | 465 | 15 | 17 | 17 | 14 | 16 | 16 | 319180000 | 249440000 | 221830000 |
| Keratin, type I cytoskeletal 18 | KRT18 | 423 | 17 | 16 | 17 | 16 | 16 | 16 | 675290000 | 596410000 | 771230000 |
| Keratin, type I cytoskeletal 28 | KRT28 | 464 | 8 | 8 | 9 | 5 | 5 | 6 | 129300000 | 129210000 | 125520000 |
| Keratin, type I cytoskeletal 9 | KRT9 | 623 | 6 | 6 | 7 | 6 | 6 | 7 | 842860000 | 681680000 | 713930000 |
| Keratin, type II cytoskeletal 1 | KRT1 | 644 | 23 | 23 | 22 | 19 | 19 | 18 | 1687700000 | 1641300000 | 1670000000 |
| Keratin, type II cytoskeletal 2 epidermal | KRT2 | 639 | 3 | 3 | 3 | 3 | 3 | 3 | 9693700 | 10583000 | 9214300 |
| Keratin, type II cytoskeletal 5 | KRT5 | 594 | 6 | 6 | 7 | 6 | 6 | 7 | 58921000 | 46576000 | 56341000 |
| Keratin, type II cytoskeletal 6A | KRT6A | 571 | 9 | 6 | 6 | 3 | 2 | 2 | 15933000 | 17201000 | 15603000 |
| Keratin, type II cytoskeletal 6B | KRT6B | 564 | 2 | 3 | 2 | 0 | 3 | 1 | 0 | 3525066 | 4437500 |
| Keratin, type II cytoskeletal 6C | KRT6C | 564 | 7 | 7 | 6 | 4 | 4 | 4 | 19366000 | 21751000 | 26127000 |
| Keratin, type II cytoskeletal 7 | KRT7 | 457 | 14 | 15 | 14 | 10 | 11 | 15 | 1151900000 | 1372600000 | 1051500000 |
| Keratin, type II cytoskeletal 71 | Krt71 | 524 | 11 | 10 | 9 | 6 | 5 | 8 | 243510000 | 219320000 | 263620000 |
| Keratin, type II cytoskeletal 73 | Krt73 | 539 | 12 | 12 | 10 | 7 | 7 | 7 | 103450000 | 100630000 | 105630000 |
| Keratin, type II cytoskeletal 78 | KRT78 | 520 | 10 | 10 | 10 | 8 | 8 | 8 | 0 | 1891158 | 1954400 |
| Keratin, type II cytoskeletal 79 | Krt79 | 531 | 16 | 16 | 16 | 10 | 10 | 10 | 133570000 | 138020000 | 137480000 |
| KH-type splicing regulatory protein | KHSRP | 708 | 1 | 1 | 1 | 1 | 1 | 1 | 0 | 2448259 | 2482600 |
| KIAA0100 | KIAA0100 | 2177 | 9 | 11 | 12 | 8 | 10 | 11 | 0 | 19357817 | 19000000 |
| KIAA1217 | KIAA1217 | 1869 | 9 | 8 | 9 | 9 | 8 | 9 | 150141 | 130469 | 120844 |
| Kinase suppressor of ras 2 | KSR2 | 950 | 3 | 3 | 3 | 3 | 3 | 3 | 6732500 | 0 | 7137300 |
| KRT15 protein | KRT15 | 453 | 13 | 14 | 12 | 9 | 11 | 14 | 337690000 | 320780000 | 353070000 |
| Lactadherin | MFGE8 | 427 | 18 | 18 | 18 | 18 | 18 | 18 | 1665300000 | 2123600000 | 1599000000 |
| Lactoferrin | LTF | 681 | 11 | 11 | 11 | 11 | 11 | 11 | 168980000 | 259090000 | 174810000 |
| Lactoperoxidase | LPO | 712 | 6 | 6 | 6 | 6 | 6 | 6 | 90837000 | 90096000 | 92677000 |
| Leucine-rich alpha-2-glycoprotein 1 | LRG1 | 346 | 2 | 2 | 2 | 2 | 2 | 2 | 14527000 | 15941000 | 15376000 |
| Lipocalin 2 | LCN2 | 200 | 2 | 2 | 2 | 2 | 2 | 2 | 23982000 | 0 | 25424000 |
| Lipocln_cytosolic_FA-bd_dom domain-containing protein | PAEP | 245 | 1 | 2 | 2 | 1 | 2 | 2 | 1462500 | 1455713 | 1431200 |
| Lipopolysaccharide-binding protein | LBP | 476 | 2 | 2 | 2 | 2 | 2 | 2 | 11360000 | 13709000 | 11816000 |
| Lipoprotein lipase | LPL | 478 | 1 | 2 | 2 | 1 | 2 | 2 | 222834 | 103721 | 142513 |
| Low affinity immunoglobulin gamma Fc region receptor II-a | FCGR2A | 271 | 3 | 4 | 4 | 3 | 4 | 4 | 14472000 | 14168984 | 15342000 |
| Lys-63-specific deubiquitinase BRCC36 | BRCC3 | 316 | 4 | 4 | 3 | 4 | 4 | 3 | 940176 | 946438 | 911890 |
| Lysyl oxidase like 3 | LOXL3 | 752 | 13 | 13 | 13 | 13 | 13 | 13 | 142650 | 162774 | 113356 |
| Melanotransferrin | MELTF | 739 | 2 | 2 | 2 | 0 | 0 | 2 | 10611000 | 0 | 11249000 |
| Metalloproteinase inhibitor 4 | TIMP4 | 224 | 2 | 1 | 2 | 2 | 1 | 2 | 6580056 | 5221947 | 6654500 |
| Microtubule crosslinking factor 1 | MTCL1 | 1837 | 7 | 7 | 7 | 7 | 1 | 1 | 423959 | 377685 | 330670 |
| Mitochondrial poly | MTPAP | 583 | 16 | 21 | 19 | 16 | 21 | 19 | 3229282 | 2926865 | 3514700 |
| Monocyte differentiation antigen CD14 | CD14 | 373 | 2 | 1 | 2 | 2 | 1 | 1 | 6349200 | 0 | 6730900 |
| MPO protein | MPO | 719 | 5 | 5 | 4 | 1 | 5 | 4 | 1896666 | 19191580 | 1971600 |
| Myoglobin | MB | 154 | 3 | 3 | 3 | 0 | 3 | 3 | 9279000 | 10416000 | 9715600 |
| Myosin light chain 1/3, skeletal muscle isoform | MYL1 | 192 | 2 | 1 | 1 | 2 | 1 | 1 | 4173846 | 4855668 | 4127100 |
| Myosin-1 | MYH1 | 1919 | 11 | 9 | 1 | 9 | 9 | 1 | 6444400 | 6675200 | 7030600 |
| Myosin-2 | MYH2 | 1940 | 12 | 10 | 12 | 2 | 2 | 0 | 55537000 | 72817000 | 57610000 |
| Myosin-7 | MYH7 | 1935 | 5 | 3 | 2 | 3 | 3 | 1 | 1312700 | 1464144 | 1391600 |
| Neuronal guanine nucleotide exchange factor | NGEF | 616 | 5 | 5 | 0 | 5 | 5 | 0 | 0 | 108120 | 107035 |
| NLR family pyrin domain containing 12 | NLRP12 | 1054 | 2 | 2 | 1 | 2 | 2 | 1 | 171091 | 178469 | 113379 |
| Nucleobindin 2 | NUCB2 | 415 | 4 | 5 | 5 | 4 | 5 | 5 | 6058800 | 0 | 6423000 |
| Nucleobindin-1 | NUCB1 | 457 | 1 | 5 | 5 | 1 | 5 | 5 | 73133000 | 64241000 | 81685000 |
| Nucleoside diphosphate kinase B | NME2 | 114 | 4 | 3 | 1 | 4 | 3 | 1 | 0 | 3698000 | 3117114 |
| Olfactory receptor | OR6S1 | 331 | 4 | 3 | 4 | 4 | 3 | 4 | 342460 | 269493 | 335140 |
| Paternally-expressed gene 3 protein | PEG3 | 2387 | 13 | 11 | 13 | 13 | 11 | 13 | 2763300 | 2319713 | 2356276 |
| Peptidase S1 domain-containing protein | LOC615237 | 233 | 1 | 10 | 11 | 1 | 10 | 11 | 0 | 19512949 | 18794000 |
| Peptidoglycan recognition protein 1 | PGLYRP1 | 190 | 3 | 4 | 0 | 3 | 4 | 0 | 92537000 | 0 | 98101000 |
| Peptidyl-prolyl cis-trans isomerase B | PPIB | 216 | 3 | 3 | 3 | 3 | 3 | 3 | 19292000 | 26082000 | 20079000 |
| Pericentrin | PCNT | 3085 | 3 | 0 | 2 | 2 | 0 | 1 | 0 | 101882250 | 106010000 |
| Perilipin-2 | PLIN2 | 450 | 4 | 4 | 4 | 4 | 4 | 4 | 87041000 | 95407000 | 95193000 |
| Perilipin-3 | PLIN3 | 438 | 5 | 5 | 4 | 5 | 5 | 4 | 4801300 | 51044800 | 5089900 |
| Peroxiredoxin-4 | PRDX4 | 260 | 2 | 2 | 2 | 2 | 2 | 1 | 3370100 | 31615143 | 3572600 |
| Peroxiredoxin-6 | PRDX6 | 224 | 11 | 11 | 12 | 2 | 2 | 2 | 0 | 1478598 | 1888000 |
| Phosphoglycerate kinase | PGK1 | 405 | 15 | 15 | 15 | 13 | 13 | 0 | 4270800 | 0 | 4527600 |
| Pigment epithelium-derived factor | SERPINF1 | 416 | 3 | 3 | 3 | 3 | 3 | 3 | 4505900 | 0 | 4776700 |
| Platelet glycoprotein 4 | CD36 | 472 | 1 | 9 | 9 | 1 | 8 | 8 | 28809000 | 30358984 | 30540000 |
| Polyamine modulated factor 1 binding protein 1 | PMFBP1 | 997 | 2 | 2 | 2 | 2 | 2 | 2 | 38343790 | 38100840 | 32257000 |
| Polymeric immunoglobulin receptor | PIGR | 757 | 8 | 0 | 8 | 8 | 0 | 8 | 192520000 | 176790000 | 202220000 |
| Profilin-1 | PFN1 | 140 | 3 | 3 | 3 | 3 | 3 | 3 | 4600000 | 4318590 | 4876500 |
| Protein SOGA3 | SOGA3 | 950 | 2 | 1 | 2 | 2 | 1 | 2 | 147974060 | 132620000 | 145149180 |
| Pyruvate kinase | PKM | 531 | 3 | 2 | 3 | 3 | 2 | 3 | 6586500 | 8859000 | 7029700 |
| RAB2A, member RAS oncogene family | RAB2A | 189 | 3 | 3 | 3 | 1 | 3 | 1 | 0 | 9691539 | 9574100 |
| Ras association domain-containing protein 3 | RASSF3 | 235 | 2 | 2 | 2 | 2 | 0 | 0 | 125714 | 121460 | 143136 |
| RAS guanyl releasing protein 1 | RASGRP1 | 797 | 2 | 2 | 1 | 2 | 2 | 1 | 819550000 | 886738901 | 868820000 |
| RAS protein activator like-3 | RASAL3 | 1012 | 1 | 2 | 2 | 1 | 2 | 2 | 120044 | 109596 | 100334 |
| Ras-related protein Rab-11A | RAB11A | 216 | 7 | 8 | 1 | 7 | 8 | 1 | 6220207 | 5684228 | 5528400 |
| Ras-related protein Rab-18 | RAB18 | 206 | 2 | 2 | 2 | 2 | 2 | 2 | 12869000 | 9743300 | 10307000 |
| Ras-related protein Rab-1B | RAB1B | 171 | 6 | 6 | 0 | 3 | 3 | 0 | 9745952 | 9072173 | 9302000 |
| Receptor protein-tyrosine kinase | EGFR | 1179 | 1 | 3 | 3 | 1 | 3 | 3 | 4368400 | 4071276 | 4631000 |
| Rho GTPase activating protein 31 | ARHGAP31 | 1312 | 11 | 8 | 10 | 8 | 8 | 1 | 0 | 4610694 | 4909700 |
| 60S ribosomal protein L27a | RPL27A | 148 | 25 | 25 | 1 | 19 | 19 | 1 | 2957664 | 2580666 | 2247600 |
| Serotransferrin | TF | 704 | 4 | 4 | 4 | 4 | 4 | 4 | 118680000 | 89633000 | 124520000 |
| Serotransferrin-like | LOC525947 | 622 | 2 | 2 | 2 | 2 | 2 | 2 | 32690000 | 30562000 | 33951000 |
| Serpin A3-1 | SERPINA3-1 | 400 | 6 | 6 | 6 | 2 | 2 | 6 | 155410000 | 167960000 | 190030000 |
| Serpin A3-6 | SERPINA3-6 | 414 | 5 | 5 | 1 | 5 | 5 | 1 | 0 | 187933 | 186064 |
| Serpin A3-8 | SERPINA3-8 | 418 | 2 | 1 | 1 | 2 | 0 | 0 | 124127 | 134608 | 103618 |
| Serum albumin | ALB | 607 | 18 | 17 | 0 | 18 | 17 | 0 | 1518800000 | 1574100000 | 1765400000 |
| Serum amyloid A protein | SAA3 | 112 | 2 | 1 | 2 | 2 | 1 | 2 | 97874000 | 0 | 103760000 |
| Signal peptide, CUB domain and EGF like domain containing 3 | SCUBE3 | 993 | 19 | 0 | 19 | 14 | 0 | 14 | 18498869 | 18660218 | 18421000 |
| Sodium/nucleoside cotransporter | SLC28A3 | 697 | 8 | 6 | 1 | 6 | 6 | 1 | 58428186 | 55002407 | 55916000 |
| Sodium-dependent phosphate transport protein 2B | SLC34A2 | 693 | 3 | 3 | 3 | 3 | 3 | 3 | 23278000 | 21159000 | 21855000 |
| Sorting nexin 16 | SNX16 | 343 | 18 | 16 | 17 | 13 | 12 | 12 | 17950405 | 17349492 | 17187000 |
| Spermatid perinuclear RNA-binding protein | STRBP | 648 | 3 | 3 | 3 | 3 | 3 | 3 | 6337400 | 0 | 6718400 |
| PHB domain-containing protein | STOM | 284 | 3 | 3 | 3 | 3 | 3 | 3 | 14353000 | 14432000 | 16717000 |
| Stromal antigen 1 | STAG1 | 998 | 4 | 4 | 4 | 4 | 4 | 4 | 25721000 | 0 | 27267000 |
| Synaptobrevin homolog YKT6 | YKT6 | 198 | 2 | 2 | 1 | 2 | 1 | 0 | 1829469 | 2192957 | 2064200 |
| TAF11 RNA polymerase II, TATA box binding protein (TBP)-associated factor, 28kDa | TAF11 | 210 | 2 | 2 | 2 | 2 | 2 | 2 | 112409 | 109581 | 106390 |
| TANK binding kinase 1 | TBK1 | 729 | 2 | 2 | 2 | 2 | 2 | 2 | 0 | 294058 | 375204 |
| Tektin-4 | TEKT4 | 447 | 5 | 5 | 5 | 1 | 2 | 1 | 732651423 | 659200901 | 639520000 |
| Lanosterol synthase | LSS | 732 | 2 | 1 | 1 | 2 | 0 | 0 | 12324332 | 12412436 | 12554000 |
| Tet methylcytosine dioxygenase 3 | TET3 | 1699 | 17 | 18 | 0 | 4 | 4 | 0 | 296150000 | 0 | 313950000 |
| Thrombospondin-1 | THBS1 | 1170 | 8 | 9 | 8 | 8 | 9 | 9 | 164650000 | 158400000 | 176470000 |
| TNF receptor-associated factor 6 | TRAF6 | 542 | 2 | 2 | 1 | 2 | 2 | 1 | 194285 | 147311 | 185131 |
| Toll-like receptor 9 | TLR9 | 1029 | 0 | 5 | 5 | 0 | 5 | 5 | 14724077 | 14884959 | 15400000 |
| Transcription factor AP-2 delta | TFAP2D | 452 | 4 | 4 | 4 | 3 | 3 | 3 | 12874962 | 10402000 | 14249000 |
| Transient receptor potential cation channel subfamily A member 1 | TRPA1 | 1119 | 4 | 0 | 4 | 4 | 0 | 4 | 11454523 | 11538923 | 10837000 |
| Transient receptor potential cation channel subfamily M member 5 | TRPM5 | 1170 | 3 | 1 | 2 | 2 | 2 | 3 | 0 | 83770 | 85438 |
| Trinucleotide repeat containing 18 | TNRC18 | 2908 | 5 | 4 | 5 | 5 | 4 | 5 | 0 | 4717612 | 4317600 |
| Tropomyosin alpha-3 chain | TPM3 | 284 | 2 | 2 | 0 | 2 | 2 | 0 | 5135071 | 4997100 | 5359900 |
| Troponin C type 2 | TNNC2 | 161 | 2 | 0 | 2 | 2 | 0 | 2 | 0 | 512740 | 434119 |
| Tyrosine-protein kinase | HCK | 558 | 2 | 2 | 2 | 2 | 2 | 2 | 112524958 | 133396000 | 138580000 |
| Ubiquitin-60S ribosomal protein L40 | UBA52 | 128 | 2 | 2 | 0 | 2 | 2 | 0 | 4445134 | 5971522 | 5979800 |
| Ubiquitin-like domain-containing protein | LOC101902760 | 77 | 2 | 2 | 2 | 2 | 2 | 2 | 15338000 | 23405000 | 15918000 |
| UMP-CMP kinase | CMPK1 | 196 | 3 | 3 | 3 | 3 | 3 | 1 | 0 | 1422702 | 1310900 |
| Urocortin | UCN | 129 | 5 | 5 | 5 | 5 | 0 | 5 | 408709 | 586440 | 649635 |
| Vesicle amine transport 1 | VAT1 | 402 | 3 | 3 | 3 | 0 | 1 | 3 | 1846061 | 2514142 | 2525500 |
| Vitamin D-binding protein | GC | 465 | 4 | 3 | 4 | 4 | 3 | 3 | 139290000 | 185130000 | 121210000 |
| Vitamin K-dependent gamma-carboxylase | GGCX | 758 | 12 | 13 | 12 | 0 | 11 | 10 | 233942 | 218350 | 237214 |
| von Willebrand factor A domain containing 3B | VWA3B | 1217 | 5 | 5 | 5 | 4 | 4 | 4 | 363880000 | 0 | 385760000 |
| Xanthine dehydrogenase/oxidase [Includes: Xanthine dehydrogenase | XDH | 1332 | 15 | 15 | 15 | 14 | 14 | 0 | 241620000 | 234990000 | 277670000 |
| Z-DNA binding protein 1 | ZBP1 | 401 | 4 | 4 | 4 | 2 | 0 | 1 | 31296000 | 33127705 | 33178000 |
| Zinc finger protein 500 | ZNF500 | 245 | 9 | 9 | 9 | 0 | 7 | 7 | 147632 | 125744 | 140469 |
| Zinc finger protein 777 | ZNF777 | 794 | 3 | 3 | 2 | 3 | 3 | 2 | 75881782 | 78902661 | 78168000 |
| Zinc-alpha-2-glycoprotein | AZGP1 | 299 | 4 | 4 | 4 | 0 | 4 | 4 | 55014000 | 52204000 | 59946000 |
| Toll like receptor-2 protein | TLR2 | 784 | 3 | 3 | 3 | 2 | 2 | 2 | 5250900 | 5549700 | 5752900 |

1. The peptides, expressed as the number of unique peptide for protein qualitative analysis.
2. Unique peptides, expressed as a peptide in a protein that exists only in the proteome of interest, although the peptide may appear multiple times in the same protein.
3. LFQ intensity, expressed as the relative abundance of protein obtained by label-free quantitative analysis, which is the correction of the original intensity among samples to eliminate the errors caused by pretreatment, sample injection and instrument.

**Table S2** Identified unique MFGM proteins compared with the previous reports.

| Protein name | Gene name | Status | Organism | Length | Biological function |
| --- | --- | --- | --- | --- | --- |
| MHC class I antigen | BOLA | unreviewed | Bos taurus (Bovine) | 360 | antigen processing and presentation [GO:0019882]; immune response [GO:0006955] |
| BPI fold containing family B member 6 | BPIFB6 | unreviewed | Bos taurus (Bovine) | 453 | lipid binding [GO:0008289] |
| Complement C4 | C4 | reviewed | Bos taurus (Bovine) | 920 | complement activation [GO:0006956]; complement activation, classical pathway [GO:0006958] |
| Cleft lip and palate transmembrane protein 1 homolog | CLPTM1 | reviewed | Bos taurus (Bovine) | 670 | cell differentiation [GO:0030154]; multicellular organism development [GO:0007275] |
| Cystatin E/M | CST6 | unreviewed | Bos taurus (Bovine) | 149 | epidermis development [GO:0008544]; cysteine-type endopeptidase inhibitor activity [GO:0004869] |
| Elongation factor 1-alpha 1 | EEF1A1 | reviewed | Bos taurus (Bovine) | 462 | GTPase activity [GO:0003924]; GTP binding [GO:0005525]; translation elongation factor activity [GO:0003746] |
| EF-hand calcium binding domain 11 | EFCAB11 | unreviewed | Bos taurus (Bovine) | 205 | calcium ion binding [GO:0005509]; enzyme regulator activity [GO:0030234] |
| Alpha-enolase | ENO1 | reviewed | Bos taurus (Bovine) | 434 | magnesium ion binding [GO:0000287]; phosphopyruvate hydratase activity [GO:0004634] |
| EPH receptor A3 | EPHA3 | unreviewed | Bos taurus (Bovine) | 984 | ATP binding [GO:0005524]; GPI-linked ephrin receptor activity [GO:0005004] |
| Histone H2B | H2B | unreviewed | Bos taurus (Bovine) | 126 | DNA binding [GO:0003677]; protein heterodimerization activity [GO:0046982] |
| Heart fatty acid-binding protein | H-FABP | unreviewed | Bos taurus (Bovine) | 133 | lipid binding [GO:0008289] |
| IGFBP7 protein | IGFBP7 | unreviewed | Bos taurus (Bovine) | 282 | insulin-like growth factor binding [GO:0005520] |
| Inter-alpha-trypsin inhibitor heavy chain H4 | ITIH4 | reviewed | Bos taurus (Bovine) | 916 | serine-type endopeptidase inhibitor activity [GO:0004867] |
| KAT8 regulatory NSL complex subunit 1 | KANSL1 | unreviewed | Bos taurus (Bovine) | 1103 | histone acetyltransferase activity (H4-K16 specific) [GO:0046972]; histone acetyltransferase activity (H4-K5 specific) [GO:0043995] |
| Kit ligand | KITLG | reviewed | Bos taurus (Bovine) | 274 | cytokine activity [GO:0005125]; growth factor activity [GO:0008083]; stem cell factor receptor binding [GO:0005173] |
| Alpha-lactalbumin | LALBA | reviewed | Bos taurus (Bovine) | 142 | calcium ion binding [GO:0005509]; identical protein binding [GO:0042802]; lactose synthase activity [GO:0004461] |
| L-lactate dehydrogenase A chain | LDHA | reviewed | Bos taurus (Bovine) | 332 | L-lactate dehydrogenase activity [GO:0004459] |
| U6 snRNA-associated Sm-like protein LSm4 | LSM4 | reviewed | Bos taurus (Bovine) | 139 | RNA binding [GO:0003723]; U6 snRNA binding [GO:0017070] |
| Myosin heavy chain 6 | MYH6 | unreviewed | Bos taurus (Bovine) | 1938 | actin-dependent ATPase activity [GO:0030898]; actin filament binding [GO:0051015] |
| Myosin heavy chain 7B | MYH7B | unreviewed | Bos taurus (Bovine) | 1928 | actin filament binding [GO:0051015]; ATP binding [GO:0005524]; motor activity [GO:0003774] |
| Myosin heavy chain 8 | MYH8 | unreviewed | Bos taurus (Bovine) | 1937 | actin filament binding [GO:0051015]; ATP binding [GO:0005524]; motor activity [GO:0003774] |
| Alpha-1-acid glycoprotein | ORM1 | reviewed | Bos taurus (Bovine) | 202 | acute-phase response [GO:0006953]; regulation of immune system process [GO:0002682] |
| Profilin-2 | PFN2 | reviewed | Bos taurus (Bovine) | 140 | actin binding [GO:0003779]; actin monomer binding [GO:0003785] |
| Peptidyl-prolyl cis-trans isomerase NIMA-interacting 4 | PIN4 | reviewed | Bos taurus (Bovine) | 131 | DNA binding [GO:0003677]; peptidyl-prolyl cis-trans isomerase activity [GO:0003755] |
| 1-phosphatidylinositol 4,5-bisphosphate phosphodiesterase gamma | PLCG2 | unreviewed | Bos taurus (Bovine) | 1262 | phosphatidylinositol phospholipase C activity [GO:0004435] |
| Serine/threonine-protein kinase PLK | PLK3 | unreviewed | Bos taurus (Bovine) | 647 | ATP binding [GO:0005524]; p53 binding [GO:0002039]; protein serine/threonine kinase activity [GO:0004674] |
| Serine protease 8 | PRSS8 | unreviewed | Bos taurus (Bovine) | 343 | serine-type endopeptidase activity [GO:0004252]; sodium channel regulator activity [GO:0017080] |
| 40S ribosomal protein SA | RPSA | reviewed | Bos taurus (Bovine) | 295 | laminin binding [GO:0043236]; laminin receptor activity [GO:0005055]; structural constituent of ribosome [GO:0003735] |
| 45 kDa calcium-binding protein | SDF4 | reviewed | Bos taurus (Bovine) | 355 | calcium ion binding [GO:0005509] |
| Serpin A3-2 | SERPINA3-2 | reviewed | Bos taurus (Bovine) | 411 | serine-type endopeptidase inhibitor activity [GO:0004867] |
| Osteopontin | SPP1 | reviewed | Bos taurus (Bovine) | 278 | cytokine activity [GO:0005125]; extracellular matrix binding [GO:0050840] |
| Stromal antigen 2 | STAG2 | unreviewed | Bos taurus (Bovine) | 1268 | chromatin binding [GO:0003682] |
| Transmembrane protein 237 | TMEM237 | reviewed | Bos taurus (Bovine) | 400 | cilium assembly [GO:0060271]; regulation of Wnt signaling pathway [GO:0030111] |
| Tubulin tyrosine ligase like 6 | TTLL6 | unreviewed | Bos taurus (Bovine) | 742 | ATP binding [GO:0005524]; protein-glutamic acid ligase activity [GO:0070739] |
| U3 small nucleolar RNA-associated protein 15 homolog | UTP15 | reviewed | Bos taurus (Bovine) | 519 | positive regulation of rRNA processing [GO:2000234]; positive regulation of transcription by RNA polymerase I [GO:0045943] |
| [F-actin]-monooxygenase | MICAL2 | reviewed | Bos taurus (Bovine) | 1101 | actin binding [GO:0003779]; FAD binding [GO:0071949] |
| Actin, aortic smooth muscle | ACTA2 | reviewed | Bos taurus (Bovine) | 377 | ATP binding [GO:0005524]; protein kinase binding [GO:0019901] |
| Actin, gamma-enteric smooth muscle | ACTG2 | reviewed | Bos taurus (Bovine) | 376 | ATP binding [GO:0005524] |
| Actin-related protein 2 | ACTR2 | reviewed | Bos taurus (Bovine) | 394 | actin binding [GO:0003779]; ATP binding [GO:0005524] |
| Adenosine deaminase RNA specific B1 | ADARB1 | unreviewed | Bos taurus (Bovine) | 721 | double-stranded RNA adenosine deaminase activity [GO:0003726]; double-stranded RNA binding [GO:0003725] |
| Adenosine monophosphate deaminase 1 (Fragment) | AMPD1 | unreviewed | Bos taurus (Bovine) | 72 | AMP deaminase activity [GO:0003876] |
| Alpha/beta hydrolase domain-containing protein 17C | ABHD17C | reviewed | Bos taurus (Bovine) | 329 | palmitoyl-(protein) hydrolase activity [GO:0008474] |
| Alpha-actinin-2 | ACTN2 | reviewed | Bos taurus (Bovine) | 894 | actin binding [GO:0003779]; calcium ion binding [GO:0005509] |
| Annexin A1 | ANXA1 | reviewed | Bos taurus (Bovine) | 346 | calcium-dependent phospholipid binding [GO:0005544] |
| Annexin A5 | ANXA5 | reviewed | Bos taurus (Bovine) | 321 | calcium-dependent phospholipid binding [GO:0005544]; calcium ion binding [GO:0005509] |
| ATOH1 protein | ATOH1 | unreviewed | Bos taurus (Bovine) | 352 | chromatin DNA binding [GO:0031490]; DNA-binding transcription activator activity, RNA polymerase II-specific [GO:0001228] |
| ATP synthase subunit alpha, mitochondrial | ATP5F1A | reviewed | Bos taurus (Bovine) | 553 | ADP binding [GO:0043531]; ATP binding [GO:0005524]; proton-transporting ATP synthase activity, rotational mechanism [GO:0046933] |
| Beta-2-glycoprotein 1 | APOH | reviewed | Bos taurus (Bovine) | 345 | heparin binding [GO:0008201] |
| Caspase recruitment domain protein 9 (Fragment) | CARD9 | unreviewed | Bos taurus (Bovine) | 534 | regulation of apoptotic process [GO:0042981] |
| Caspase-4 | CASP4 | reviewed | Bos taurus (Bovine) | 377 | cysteine-type endopeptidase activity [GO:0004197]; cysteine-type endopeptidase activity involved in apoptotic signaling pathway [GO:0097199] |
| Chitinase-3-like protein 1 | CHI3L1 | reviewed | Bos taurus (Bovine) | 383 | carbohydrate binding [GO:0030246]; chitinase activity [GO:0004568]; chitin binding [GO:0008061] |
| Cholinergic receptor nicotinic alpha 6 subunit | CHRNA6 | unreviewed | Bos taurus (Bovine) | 494 | acetylcholine-gated cation-selective channel activity [GO:0022848]; transmembrane signaling receptor activity [GO:0004888] |
| Chromodomain helicase DNA binding protein 3 | CHD3 | unreviewed | Bos taurus (Bovine) | 1972 | ATP binding [GO:0005524]; metal ion binding [GO:0046872] |
| Corneodesmosin | CDSN | unreviewed | Bos taurus (Bovine) | 559 | protein homodimerization activity [GO:0042803] |
| Homeobox protein cut-like | CUX1 | unreviewed | Bos taurus (Bovine) | 1534 | RNA polymerase II regulatory region sequence-specific DNA binding [GO:0000977] |
| Desmoglein-1 | DSG1 | reviewed | Bos taurus (Bovine) | 1043 | calcium ion binding [GO:0005509]; gamma-catenin binding [GO:0045295] |
| Disco interacting protein 2 homolog B | DIP2B | unreviewed | Bos taurus (Bovine) | 1575 | catalytic activity [GO:0003824] |
| DNA damage induced apoptosis suppressor | DDIAS | unreviewed | Bos taurus (Bovine) | 918 | mitotic cell cycle arrest [GO:0071850]; regulation of DNA stability [GO:0097752] |
| DNA excision repair protein ERCC-6-like 2 | ERCC6L2 | reviewed | Bos taurus (Bovine) | 1558 | ATP binding [GO:0005524]; DNA binding [GO:0003677]; helicase activity [GO:0004386] |
| FAD-binding PCMH-type domain-containing protein | AOX4 | unreviewed | Bos taurus (Bovine) | 1327 | 2 iron, 2 sulfur cluster binding [GO:0051537]; electron transfer activity [GO:0009055] |
| Fibrous sheath interacting protein 2 | FSIP2 | unreviewed | Bos taurus (Bovine) | 6749 | protein localization to cilium [GO:0061512]; sperm axoneme assembly [GO:0007288] |
| G_PROTEIN_RECEP_F1_2 domain-containing protein | OR10G2 | unreviewed | Bos taurus (Bovine) | 318 | G protein-coupled receptor activity [GO:0004930]; olfactory receptor activity [GO:0004984] |
| Glucosidase 2 subunit beta | PRKCSH | reviewed | Bos taurus (Bovine) | 533 | calcium ion binding [GO:0005509]; hydrolase activity [GO:0016787] |
| Growth factor receptor-bound protein 14 | GRB14 | reviewed | Bos taurus (Bovine) | 540 | SH3/SH2 adaptor activity [GO:0005070] |
| Guanine nucleotide-binding protein G(s) subunit alpha isoforms short | GNAS | reviewed | Bos taurus (Bovine) | 394 | adenylate cyclase activator activity [GO:0010856]; beta-2 adrenergic receptor binding [GO:0031698] |
| Histone acetyltransferase | KAT2B | unreviewed | Bos taurus (Bovine) | 799 | histone acetyltransferase activity [GO:0004402] |
| Histone cluster 1 H1 family member c | HIST1H1C | unreviewed | Bos taurus (Bovine) | 227 | chromatin DNA binding [GO:0031490] |
| HOXB3 protein | HOXB3 | unreviewed | Bos taurus (Bovine) | 426 | DNA-binding transcription activator activity, RNA polymerase II-specific [GO:0001228] |
| Katanin p60 ATPase-containing subunit A1 | KATNAL1 | unreviewed | Bos taurus (Bovine) | 364 | ATP binding [GO:0005524]; isomerase activity [GO:0016853]; microtubule binding [GO:0008017] |
| KH-type splicing regulatory protein | KHSRP | unreviewed | Bos taurus (Bovine) | 708 | RNA binding [GO:0003723] |
| KIAA1217 | KIAA1217 | unreviewed | Bos taurus (Bovine) | 1869 | embryonic skeletal system development [GO:0048706] |
| Lys-63-specific deubiquitinase BRCC36 | BRCC3 | reviewed | Bos taurus (Bovine) | 316 | enzyme regulator activity [GO:0030234]; metal ion binding [GO:0046872] |
| Metalloproteinase inhibitor 4 | TIMP4 | reviewed | Bos taurus (Bovine) | 224 | metal ion binding [GO:0046872]; metalloendopeptidase inhibitor activity [GO:0008191]; protease binding [GO:0002020] |
| Microtubule crosslinking factor 1 | MTCL1 | unreviewed | Bos taurus (Bovine) | 1837 | microtubule binding [GO:0008017] |
| Neuronal guanine nucleotide exchange factor | NGEF | unreviewed | Bos taurus (Bovine) | 616 | guanyl-nucleotide exchange factor activity [GO:0005085] |
| Olfactory receptor | OR6S1 | unreviewed | Bos taurus (Bovine) | 331 | G protein-coupled receptor activity [GO:0004930]; olfactory receptor activity [GO:0004984] |
| Paternally-expressed gene 3 protein | PEG3 | reviewed | Bos taurus (Bovine) | 2387 | DNA-binding transcription factor activity [GO:0003700]; metal ion binding [GO:0046872] |
| Peptidase S1 domain-containing protein | LOC615237 | unreviewed | Bos taurus (Bovine) | 233 | serine-type endopeptidase activity [GO:0004252] |
| Peptidoglycan recognition protein 1 | PGLYRP1 | reviewed | Bos taurus (Bovine) | 190 | N-acetylmuramoyl-L-alanine amidase activity [GO:0008745]; peptidoglycan binding [GO:0042834] |
| Pyruvate kinase | PKM | unreviewed | Bos taurus (Bovine) | 531 | kinase activity [GO:0016301]; magnesium ion binding [GO:0000287] |
| Ras protein activator like-3 | RASAL3 | reviewed | Bos taurus (Bovine) | 1012 | GTPase activator activity [GO:0005096] |
| Ras-related protein Rab-11A | RAB11A | reviewed | Bos taurus (Bovine) | 216 | GTPase activity [GO:0003924]; GTP binding [GO:0005525] |
| 60S ribosomal protein L27a | RPL27A | reviewed | Bos taurus (Bovine) | 148 | structural constituent of ribosome [GO:0003735] |
| Serpin A3-6 | SERPINA3-6 | reviewed | Bos taurus (Bovine) | 414 | serine-type endopeptidase inhibitor activity [GO:0004867] |
| Serpin A3-8 | SERPINA3-8 | reviewed | Bos taurus (Bovine) | 418 | serine-type endopeptidase inhibitor activity [GO:0004867] |
| Signal peptide, CUB domain and EGF like domain containing 3 | SCUBE3 | unreviewed | Bos taurus (Bovine) | 993 | calcium ion binding [GO:0005509] |
| Sorting nexin 16 | SNX16 | unreviewed | Bos taurus (Bovine) | 343 | identical protein binding [GO:0042802]; phosphatidylinositol binding [GO:0035091] |
| STOM protein | STOM | unreviewed | Bos taurus (Bovine) | 284 | protein homodimerization activity [GO:0042803]; RNA polymerase binding [GO:0070063] |
| TAF11 RNA polymerase II, TATA box binding protein (TBP)-associated factor, 28kDa | TAF11 | unreviewed | Bos taurus (Bovine) | 210 | protein heterodimerization activity [GO:0046982]; transcription coactivator activity [GO:0003713] |
| TANK binding kinase 1 | TBK1 | unreviewed | Bos taurus (Bovine) | 729 | ATP binding [GO:0005524]; identical protein binding [GO:0042802]; nucleic acid binding [GO:0003676] |
| Tektin-4 | TEKT4 | reviewed | Bos taurus (Bovine) | 447 | cilium assembly [GO:0060271]; cilium movement involved in cell motility [GO:0060294]; regulation of brood size [GO:0060378] |
| Lanosterol synthase | LSS | reviewed | Bos taurus (Bovine) | 732 | beta-amyrin synthase activity [GO:0042300]; lanosterol synthase activity [GO:0000250] |
| TNF receptor-associated factor 6 | TRAF6 | reviewed | Bos taurus (Bovine) | 542 | histone deacetylase binding [GO:0042826]; identical protein binding [GO:0042802] |
| Toll-like receptor 9 | TLR9 | reviewed | Bos taurus (Bovine) | 1029 | pattern recognition receptor activity [GO:0038187]; protein homodimerization activity [GO:0042803] |
| Transcription factor AP-2 delta | TFAP2D | unreviewed | Bos taurus (Bovine) | 452 | DNA-binding transcription factor activity, RNA polymerase II-specific [GO:0000981]; RNA polymerase II regulatory region sequence-specific DNA binding [GO:0000977] |
| Transient receptor potential cation channel subfamily A member 1 | TRPA1 | unreviewed | Bos taurus (Bovine) | 1119 | calcium-release channel activity [GO:0015278]; identical protein binding [GO:0042802]; temperature-gated cation channel activity [GO:0097604] |
| Transient receptor potential cation channel subfamily M member 5 | TRPM5 | unreviewed | Bos taurus (Bovine) | 1170 | calcium activated cation channel activity [GO:0005227] |
| Trinucleotide repeat containing 18 | TNRC18 | unreviewed | Bos taurus (Bovine) | 2908 | chromatin binding [GO:0003682] |
| Troponin C type 2 | TNNC2 | unreviewed | Bos taurus (Bovine) | 161 | calcium ion binding [GO:0005509] |
| Urocortin | UCN | unreviewed | Bos taurus (Bovine) | 129 | hormone activity [GO:0005179] |
| Zinc finger protein 500 | ZNF500 | unreviewed | Bos taurus (Bovine) | 245 | DNA-binding transcription factor activity [GO:0003700] |
| Toll-like receptor 2 | TLR2 | reviewed | Bos taurus (Bovine) | 784 | NAD(P)+ nucleosidase activity [GO:0050135]; NAD+ nucleotidase, cyclic ADP-ribose generating [GO:0061809] |
| 1-acyl-sn-glycerol-3-phosphate acyltransferase | AGPAT1 | unreviewed | Capra hircus (Goat) | 287 | endoplasmic reticulum [GO:0005783]; integral component of membrane [GO:0016021] |
| Annexin A11 | ANXA11 | unreviewed | Capra hircus (Goat) | 503 | azurophil granule [GO:0042582]; cytosol [GO:0005829]; midbody [GO:0030496]; nuclear envelope [GO:0005635] |
| Rho-GAP domain-containing protein | ARHGAP31 | unreviewed | Capra hircus (Goat) | 1440 | lamellipodium [GO:0030027]; GTPase activator activity [GO:0005096]; SH3 domain binding [GO:0017124] |
| Adipose triglyceride lipase | ATGL | unreviewed | Capra hircus (Goat) | 486 | triglyceride lipase activity [GO:0004806]; lipid catabolic process [GO:0016042] |
| Cation-transporting ATPase | ATP13A4 | unreviewed | Capra hircus (Goat) | 1197 | integral component of membrane [GO:0016021]; ATP binding [GO:0005524] |
| ATP synthase subunit beta | ATP5F1B | unreviewed | Capra hircus (Goat) | 528 | cell surface [GO:0009986]; mitochondrial nucleoid [GO:0042645] |
| Beta-2-microglobulin | B2M | unreviewed | Capra hircus (Goat) | 118 | extracellular region [GO:0005576]; MHC class I protein complex [GO:0042612] |
| Biglycan | BGN | unreviewed | Capra hircus (Goat) | 369 | cell surface [GO:0009986]; extracellular region [GO:0005576]; transport vesicle [GO:0030133] |
| GLTSCR1 domain-containing protein | BICRA | unreviewed | Capra hircus (Goat) | 1467 | SWI/SNF complex [GO:0016514]; transcription coactivator activity [GO:0003713] |
| Bactericidal permeability-increasing protein | BPI | unreviewed | Capra hircus (Goat) | 462 | cytoplasm [GO:0005737]; extracellular space [GO:0005615]; membrane [GO:0016020] |
| Carbamoyl-phosphate synthetase 2, aspartate transcarbamylase, and dihydroorotase | CAD | unreviewed | Capra hircus (Goat) | 2162 | cytosol [GO:0005829]; nuclear matrix [GO:0016363]; nucleoplasm [GO:0005654] |
| T-complex protein 1 subunit delta | CCT4 | unreviewed | Capra hircus (Goat) | 502 | cytoplasm [GO:0005737]; ATP binding [GO:0005524] |
| Monocyte differentiation antigen CD14 | CD14 | unreviewed | Capra hircus (Goat) | 371 | anchored component of external side of plasma membrane [GO:0031362] |
| Leukocyte surface antigen CD47 | CD47 | unreviewed | Capra hircus (Goat) | 267 | integral component of membrane [GO:0016021]; plasma membrane [GO:0005886] |
| Saposin B-type domain-containing protein | CNPY2 | unreviewed | Capra hircus (Goat) | 182 | negative regulation of gene expression [GO:0010629] |
| Catechol O-methyltransferase | COMT | unreviewed | Capra hircus (Goat) | 272 | integral component of membrane [GO:0016021]; intracellular membrane-bounded organelle [GO:0043231] |
| Coatomer subunit alpha | COPA | unreviewed | Capra hircus (Goat) | 1224 | COPI vesicle coat [GO:0030126]; extracellular space [GO:0005615] |
| Pentaxin | CRP | unreviewed | Capra hircus (Goat) | 221 | extracellular region [GO:0005576]; metal ion binding [GO:0046872] |
| Zeta-crystallin | CRYZ | unreviewed | Capra hircus (Goat) | 330 | cytosol [GO:0005829]; identical protein binding [GO:0042802] |
| Dynein axonemal heavy chain 7 | DNAH7 | unreviewed | Capra hircus (Goat) | 4016 | cilium [GO:0005929]; cytoplasm [GO:0005737] |
| SH3 domain-containing protein | DSP | unreviewed | Capra hircus (Goat) | 2748 | basolateral plasma membrane [GO:0016323]; cornified envelope [GO:0001533] |
| S1 motif domain-containing protein | EIF2S1 | unreviewed | Capra hircus (Goat) | 315 | cytoplasmic stress granule [GO:0010494]; translation initiation factor activity [GO:0003743] |
| Eukaryotic translation initiation factor 2 subunit 3 structural gene Y-linked | Eif2s3y | unreviewed | Capra hircus (Goat) | 470 | GTP binding [GO:0005525]; GTPase activity [GO:0003924] |
| Eukaryotic translation initiation factor 3 subunit A | EIF3A | unreviewed | Capra hircus (Goat) | 1332 | eukaryotic 43S preinitiation complex [GO:0016282]; eukaryotic 48S preinitiation complex [GO:0033290] |
| Eukaryotic translation initiation factor 3 subunit M | EIF3M | unreviewed | Capra hircus (Goat) | 374 | eukaryotic 43S preinitiation complex [GO:0016282]; eukaryotic 48S preinitiation complex [GO:0033290] |
| Eukaryotic translation initiation factor 5A | EIF5A2 | unreviewed | Capra hircus (Goat) | 142 | endoplasmic reticulum membrane [GO:0005789]; nuclear pore [GO:0005643] |
| Endoplasmic reticulum protein 29 | ERP29 | unreviewed | Capra hircus (Goat) | 259 | endoplasmic reticulum lumen [GO:0005788]; melanosome [GO:0042470] |
| Folate receptor alpha | FOLR1 | unreviewed | Capra hircus (Goat) | 251 | folic acid binding [GO:0005542]; folic acid receptor activity [GO:0061714] |
| NAD | GALE | unreviewed | Capra hircus (Goat) | 348 | protein homodimerization activity [GO:0042803]; UDP-glucose 4-epimerase activity [GO:0003978] |
| Gelsolin | GSN | unreviewed | Capra hircus (Goat) | 780 | actin filament binding [GO:0051015]; calcium ion binding [GO:0005509] |
| Hemoglobin subunit beta-C | HBBC | reviewed | Capra hircus (Goat) | 141 | hemoglobin complex [GO:0005833]; heme binding [GO:0020037] |
| Hemopexin | HPX | unreviewed | Capra hircus (Goat) | 459 | extracellular space [GO:0005615]; heme transmembrane transporter activity [GO:0015232] |
| Stress-70 protein, mitochondrial | HSPA9 | unreviewed | Capra hircus (Goat) | 679 | mitochondrial nucleoid [GO:0042645]; ATP binding [GO:0005524] |
| VWFA domain-containing protein | ITGAM | unreviewed | Capra hircus (Goat) | 1152 | cell surface [GO:0009986]; extracellular space [GO:0005615] |
| BTB domain-containing protein | KCNS1 | unreviewed | Capra hircus (Goat) | 524 | perinuclear region of cytoplasm [GO:0048471]; voltage-gated potassium channel complex [GO:0008076] |
| LIM and SH3 domain protein 1 | LASP1 | unreviewed | Capra hircus (Goat) | 260 | cell cortex [GO:0005938]; cytoskeleton [GO:0005856] |
| L-lactate dehydrogenase | LDHB | unreviewed | Capra hircus (Goat) | 334 | cytoplasm [GO:0005737]; L-lactate dehydrogenase activity [GO:0004459] |
| PLAT domain-containing protein | LIPG | unreviewed | Capra hircus (Goat) | 500 | cell surface [GO:0009986]; chylomicron [GO:0042627] |
| Methyltransf_11 domain-containing protein | LOC102168424 | unreviewed | Capra hircus (Goat) | 244 | integral component of membrane [GO:0016021] |
| Tubulin alpha chain | LOC102178426 | unreviewed | Capra hircus (Goat) | 433 | cytoplasm [GO:0005737]; microtubule [GO:0005874] |
| LRAT domain-containing protein | LRATD2 | unreviewed | Capra hircus (Goat) | 288 | cytoplasm [GO:0005737]; plasma membrane [GO:0005886] |
| Leucine rich repeat containing 24 | LRRC24 | unreviewed | Capra hircus (Goat) | 440 | integral component of membrane [GO:0016021] |
| T-lymphocyte surface antigen Ly-9 | LY9 | unreviewed | Capra hircus (Goat) | 338 | integral component of membrane [GO:0016021] |
| Methionine--tRNA ligase, cytoplasmic | MARS1 | unreviewed | Capra hircus (Goat) | 893 | aminoacyl-tRNA synthetase multienzyme complex [GO:0017101]; cytosol [GO:0005829] |
| Myeloperoxidase | MPO | unreviewed | Capra hircus (Goat) | 719 | heme binding [GO:0020037]; peroxidase activity [GO:0004601] |
| Perilipin-2 | PLIN2 | unreviewed | Capra hircus (Goat) | 378 | cytosol [GO:0005829]; lipid droplet [GO:0005811] |
| Perilipin-3 | PLIN3 | unreviewed | Capra hircus (Goat) | 427 | cytosol [GO:0005829]; lipid droplet [GO:0005811] |
| Polyamine modulated factor 1 binding protein 1 | PMFBP1 | unreviewed | Capra hircus (Goat) | 869 | cytoplasm [GO:0005737]; sperm connecting piece [GO:0097224] |
| AAA domain-containing protein | PSMC3 | unreviewed | Capra hircus (Goat) | 442 | nucleus [GO:0005634]; P-body [GO:0000932] |
| PCI domain-containing protein | PSMD6 | unreviewed | Capra hircus (Goat) | 389 | proteasome complex [GO:0000502]; enzyme regulator activity [GO:0030234] |
| RAP2B protein | RAP2B | unreviewed | Capra hircus (Goat) | 183 | bicellular tight junction [GO:0005923]; cell-cell contact zone [GO:0044291] |
| Retinol binding protein 1 | RBP1 | unreviewed | Capra hircus (Goat) | 135 | cytosol [GO:0005829]; lipid droplet [GO:0005811] |
| Ribonuclease/angiogenin inhibitor 1 | RNH1 | unreviewed | Capra hircus (Goat) | 456 | angiogenin-PRI complex [GO:0032311]; cytosol [GO:0005829] |
| Ribosomal_L16 domain-containing protein | RPL10L | unreviewed | Capra hircus (Goat) | 214 | cytosol [GO:0005829]; endoplasmic reticulum [GO:0005783] |
| 60S ribosomal protein L7a | RPL7A | unreviewed | Capra hircus (Goat) | 246 | cytosolic large ribosomal subunit [GO:0022625]; RNA binding [GO:0003723] |
| Dolichyl-diphosphooligosaccharide--protein glycosyltransferase subunit 1 | RPN1 | unreviewed | Capra hircus (Goat) | 585 | integral component of membrane [GO:0016021]; oligosaccharyltransferase complex [GO:0008250] |
| 40S ribosomal protein S12 | RPS12 | unreviewed | Capra hircus (Goat) | 126 | ribosome [GO:0005840]; structural constituent of ribosome [GO:0003735] |
| Ribosomal_S13_N domain-containing protein | RPS13 | unreviewed | Capra hircus (Goat) | 151 | ribosome [GO:0005840]; structural constituent of ribosome [GO:0003735] |
| Ribosomal protein S17 | RPS17 | unreviewed | Capra hircus (Goat) | 135 | ribosome [GO:0005840]; structural constituent of ribosome [GO:0003735] |
| 40S ribosomal protein S24 | RPS24 | unreviewed | Capra hircus (Goat) | 131 | ribosome [GO:0005840]; structural constituent of ribosome [GO:0003735] |
| Ribosomal_S7 domain-containing protein | RPS5 | unreviewed | Capra hircus (Goat) | 204 | cytosolic small ribosomal subunit [GO:0022627]; mRNA binding [GO:0003729] |
| 40S ribosomal protein S7 | RPS7 | unreviewed | Capra hircus (Goat) | 174 | centrosome [GO:0005813]; cytosolic small ribosomal subunit [GO:0022627] |
| Reticulon | RTN4 | unreviewed | Capra hircus (Goat) | 198 | endoplasmic reticulum membrane [GO:0005789]; integral component of membrane [GO:0016021] |
| Protein S100-A12 | S100A12 | unreviewed | Capra hircus (Goat) | 92 | cytoplasm [GO:0005737]; nucleus [GO:0005634] |
| Ig-like domain-containing protein | SCN2B | unreviewed | Capra hircus (Goat) | 265 | voltage-gated sodium channel complex [GO:0001518]; sodium channel regulator activity [GO:0017080] |
| Selenoprotein F | SELENOF | unreviewed | Capra hircus (Goat) | 124 | endoplasmic reticulum lumen [GO:0005788] |
| PX domain-containing protein | SNX30 | unreviewed | Capra hircus (Goat) | 437 | phosphatidylinositol binding [GO:0035091]; protein transport [GO:0015031] |
| Signal sequence receptor subunit 1 | SSR1 | unreviewed | Capra hircus (Goat) | 280 | endoplasmic reticulum membrane [GO:0005789]; integral component of membrane [GO:0016021] |
| T-complex protein 1 subunit alpha | TCP1 | unreviewed | Capra hircus (Goat) | 556 | acrosomal vesicle [GO:0001669]; cell body [GO:0044297] |
| Thrombospondin-1 | THBS1 | unreviewed | Capra hircus (Goat) | 1126 | extracellular space [GO:0005615]; calcium ion binding [GO:0005509] |
| Tetraspanin-1 | TSPAN1 | unreviewed | Capra hircus (Goat) | 236 | cell junction [GO:0030054]; integral component of membrane [GO:0016021] |
| Ubiquitin carboxyl-terminal hydrolase | USP10 | unreviewed | Capra hircus (Goat) | 782 | cytoplasm [GO:0005737]; thiol-dependent ubiquitinyl hydrolase activity [GO:0036459] |
| Vimentin | VIM | unreviewed | Capra hircus (Goat) | 388 | cell leading edge [GO:0031252]; cytosol [GO:0005829] |
| UBA domain-containing protein | VPS13D | unreviewed | Capra hircus (Goat) | 4364 | mitochondrion organization [GO:0007005]; positive regulation of mitophagy [GO:1901526] |
| Vps53_N domain-containing protein | VPS53 | unreviewed | Capra hircus (Goat) | 832 | cytosol [GO:0005829]; EARP complex [GO:1990745] |
| WRN RecQ like helicase | WRN | unreviewed | Capra hircus (Goat) | 1382 | centrosome [GO:0005813]; chromosome, telomeric region [GO:0000781] |
| Acyl-CoA synthetase short-chain family member 3 | ACSS3 | unreviewed | Capra hircus (Goat) | 678 | mitochondrial matrix [GO:0005759]; acetate-CoA ligase activity [GO:0003987] |
| Aldehyde dehydrogenase family 3 member B2 | ALDH3B2 | unreviewed | Capra hircus (Goat) | 466 | oxidoreductase activity, acting on the aldehyde or oxo group of donors, NAD or NADP as acceptor [GO:0016620] |
| Fructose-bisphosphate aldolase | ALDOC | unreviewed | Capra hircus (Goat) | 364 | cytoskeletal protein binding [GO:0008092]; fructose-bisphosphate aldolase activity [GO:0004332] |
| Annexin A3 | ANXA3 | unreviewed | Capra hircus (Goat) | 319 | calcium ion binding [GO:0005509]; calcium-dependent phospholipid binding [GO:0005544] |
| AP complex subunit sigma | AP1S2 | unreviewed | Capra hircus (Goat) | 157 | membrane coat [GO:0030117]; intracellular protein transport [GO:0006886] |
| Actin-related protein 2/3 complex subunit 5 | ARPC5 | unreviewed | Capra hircus (Goat) | 151 | Arp2/3 protein complex [GO:0005885]; cytoplasm [GO:0005737] |
| V-type proton ATPase subunit B | ATP6V1B1 | unreviewed | Capra hircus (Goat) | 402 | apical plasma membrane [GO:0016324]; basolateral plasma membrane [GO:0016323] |
| Bac7.5 protein | bac7.5 | unreviewed | Capra hircus (Goat) | 190 | extracellular region [GO:0005576]; defense response to bacterium [GO:0042742] |
| Cathelicidin-2 | CATHL2 | reviewed | Capra hircus (Goat) | 176 | extracellular region [GO:0005576]; defense response to bacterium [GO:0042742] |
| SAP domain-containing protein | CCAR1 | unreviewed | Capra hircus (Goat) | 1138 | cytoplasm [GO:0005737]; cell cycle [GO:0007049] |
| DUF4200 domain-containing protein | CCDC42 | unreviewed | Capra hircus (Goat) | 316 | spermatid development [GO:0007286] |
| CD81 antigen | CD81 | unreviewed | Capra hircus (Goat) | 236 | basal plasma membrane [GO:0009925]; extracellular exosome [GO:0070062] |
| Creatine kinase M-type | CKM | unreviewed | Capra hircus (Goat) | 381 | extracellular space [GO:0005615]; ATP binding [GO:0005524] |
| Cleavage stimulation factor subunit 2 | CSTF2 | unreviewed | Capra hircus (Goat) | 190 | mRNA cleavage and polyadenylation specificity factor complex [GO:0005847] |
| Cytochrome b-245 light chain | CYBA | unreviewed | Capra hircus (Goat) | 67 | endosome [GO:0005768]; NADPH oxidase complex [GO:0043020] |
| Eukaryotic initiation factor 4A-II | EIF4A2 | unreviewed | Capra hircus (Goat) | 363 | perinuclear region of cytoplasm [GO:0048471] |
| Neutrophil elastase | ELANE | unreviewed | Capra hircus (Goat) | 267 | cell surface [GO:0009986]; extracellular space [GO:0005615] |
| FERM domain-containing protein | EZR | unreviewed | Capra hircus (Goat) | 551 | actin filament [GO:0005884]; brush border [GO:0005903] |
| Gal_mutarotas_2 domain-containing protein | GANAB | unreviewed | Capra hircus (Goat) | 869 | glucosidase II complex [GO:0017177]; carbohydrate binding [GO:0030246] |
| SCP domain-containing protein | GLIPR1L1 | unreviewed | Capra hircus (Goat) | 284 | extracellular region [GO:0005576] |
| GLTP domain-containing protein | GLTP | unreviewed | Capra hircus (Goat) | 209 | cytoplasm [GO:0005737]; lipid transfer activity [GO:0120013] |
| INT_SG_DDX_CT_C domain-containing protein | INTS6 | unreviewed | Capra hircus (Goat) | 828 | actin cytoskeleton [GO:0015629]; integrator complex [GO:0032039] |
| Inos-1-P_synth domain-containing protein | ISYNA1 | unreviewed | Capra hircus (Goat) | 547 | inositol-3-phosphate synthase activity [GO:0004512]; inositol biosynthetic process [GO:0006021] |
| Integrin beta | ITGB2 | reviewed | Capra hircus (Goat) | 750 | integrin complex [GO:0008305]; membrane [GO:0016020] |
| Kinesin-like protein | KIF3A | unreviewed | Capra hircus (Goat) | 858 | microtubule [GO:0005874]; ATP binding [GO:0005524] |
| ATP synthase subunit alpha | LOC102183240 | unreviewed | Capra hircus (Goat) | 547 | plasma membrane [GO:0005886] |
| Myoglobin | MB | reviewed | Capra hircus (Goat) | 154 | heme binding [GO:0020037]; metal ion binding [GO:0046872] |
| Mediator of RNA polymerase II transcription subunit 4 | MED4 | unreviewed | Capra hircus (Goat) | 197 | mediator complex [GO:0016592]; transcription coregulator activity [GO:0003712] |
| Methyltransferase like 9 | METTL9 | unreviewed | Capra hircus (Goat) | 256 | methyltransferase activity [GO:0008168]; methylation [GO:0032259] |
| WRNPLPNID domain-containing protein | PPP4R1 | unreviewed | Capra hircus (Goat) | 960 | protein phosphatase 4 complex [GO:0030289] |
| Myeloblastin | PRTN3 | unreviewed | Capra hircus (Goat) | 253 | azurophil granule lumen [GO:0035578]; cytosol [GO:0005829] |
| Resistin | RETN | unreviewed | Capra hircus (Goat) | 109 | extracellular region [GO:0005576]; hormone activity [GO:0005179] |
| Dolichyl-diphosphooligosaccharide--protein glycosyltransferase subunit 2 | RPN2 | unreviewed | Capra hircus (Goat) | 509 | integral component of membrane [GO:0016021] |
| SERPIN domain-containing protein | SERPINC1 | unreviewed | Capra hircus (Goat) | 462 | extracellular space [GO:0005615]; heparin binding [GO:0008201] |
| Solute carrier family 2, facilitated glucose transporter member 3 | SLC2A3 | unreviewed | Capra hircus (Goat) | 494 | integral component of membrane [GO:0016021] |
| Solute carrier family 31 member 1 | SLC31A1 | unreviewed | Capra hircus (Goat) | 189 | integral component of membrane [GO:0016021]; late endosome [GO:0005770] |
| Toll like receptor-2 protein | TLR-2 | unreviewed | Capra hircus (Goat) | 784 | integral component of membrane [GO:0016021]; membrane raft [GO:0045121] |
| Urocortin-3 | UCN3 | unreviewed | Capra hircus (Goat) | 29 | extracellular space [GO:0005615]; corticotropin-releasing hormone receptor 2 binding [GO:0051431] |

1. * Corresponding author. Phone: +86-029-86168583, Fax: +86-029-86168583.

   *E-mail address:* jiawei@sust.edu. (W. Jia) [↑](#footnote-ref-2)
